# Efficacy and safety of huanglian wendan decoction as an adjuvant therapy for metabolic syndrome: a systematic review and meta-analysis

**DOI:** 10.3389/fphar.2026.1762428

**Published:** 2026-06-16

**Authors:** Tianpei Jiang, Jiantong Dong, Yaxin Zhu, Jinna Nie

**Affiliations:** Basic Medical College, Changchun University of Chinese Medicine, Changchun, Jilin, China

**Keywords:** Huanglian Wendan Decoction, meta analysis, metabolic syndrome, systematic review, traditional Chinese medicine

## Abstract

**Background:**

Metabolic syndrome (MetS) is a prevalent metabolic disorder with a rising global prevalence, serving as a risk factor for cardiovascular disease and type 2 diabetes. Huanglian Wendan Decoction, a commonly used formula in traditional Chinese medicine, has increasingly been used to treat MetS. This study aims to evaluate the efficacy and safety of Huanglian Wendan Decoction as an adjunctive therapy for MetS.

**Objective:**

This meta-analysis aims to systematically evaluate the efficacy and safety of Huanglian Wendan Decoction in treating metabolic syndrome, thereby providing new approaches and insights for its management.

**Methods:**

Literature searches were conducted using English-language databases (PubMed, Web of Science, Embase, and the Cochrane Library) and Chinese databases (China National Knowledge Infrastructure Wanfang, the VIP Chinese Science Journal Database, and the Chinese Biomedical Literature Database). The search period extended through 1 October 2025. Data were analyzed using RevMan 5.3 and Stata 17.0. The study protocol is registered in PROSPERO (registration number: CRD420251151304).

**Results:**

A total of 17 randomized controlled trials, involving 1,549 patients, were ultimately included. Huanglian Wendan Decoction combined with conventional treatment for MetS significantly improved waist circumference (WC) (MD = −1.82 cm, 95% CI:−2.53 to −1.12, P < 0.00001), body mass index (BMI) (MD = −0.76 kg/m2, 95% CI: −0.99 to −0.54, P < 0.00001), systolic blood pressure (SBP) (MD = −6.62 mmHg, 95% CI: −7.58 to −5.66, P < 0.00001), diastolic blood pressure (DBP) (MD = −4.88 mmHg, 95% CI: −5.69 to −4.08, P < 0.00001), fasting plasma glucose (FPG) (MD = −0.71 mmol/L, 95% CI: −0.81 to −0.61, P < 0.00001), 2-h postprandial glucose (2 hPG) (MD = −0.71 mmol/L, 95% CI: −0.83 to −0.58, P < 0.00001), glycated haemoglobin (HbA1c) (MD = −0.47%, 95% CI: −0.68 to −0.26, P < 0.00001), triglycerides (TG) (MD = −0.38 mmol/L, 95% CI: −0.44 to −0.33, P < 0.00001), low-density lipoprotein cholesterol (LDL-C) (MD = −0.57 mmol/L, 95% CI: −0.73 to −0.42, P < 0.00001), and homeostasis model assessment of insulin resistance (HOMA-IR) (MD = −0.93, 95% CI: −1.09 to −0.78, P < 0.00001). However, conventional therapy demonstrated greater improvement in high-density lipoprotein cholesterol (HDL-C) (MD = 0.16 mmol/L, 95% CI: 0.14 to 0.18, P < 0.00001). Nevertheless, the safety of Huanglian Wendan Decoction combined with conventional therapy for MetS remains inconclusive, as the safety indicators showed no statistically significant differences (OR = 0.24, 95% CI: 0.03 to 2.26, P = 0.21).

**Conclusion:**

According to the results of the meta-analysis, Huanglian Wendan Decoction combined with standard treatment for metabolic syndrome (MetS) offers greater benefits than standard treatment alone; it can control blood pressure, blood lipid levels, and blood glucose, improve waist circumference (WC) and body mass index (BMI), and reduce insulin resistance. Huanglian Wendan Decoction is relatively safe. However, due to the limited number and quality of the included studies, the data from this study remain uncertain; further large-scale, double-blind, randomised controlled trials are required to validate these findings.

**Systematic Review Registration:**

https://www.crd.york.ac.uk/PROSPERO/view/CRD420251151304. Identifier CRD420251151304.

## 1 Introduction

Metabolic syndrome (MetS) is a clinical syndrome characterised by insulin resistance, visceral obesity, atherogenic dyslipidaemia, and endothelial dysfunction (Huang, 2009), encompassing obesity, hypertension, hyperglycaemia, and dyslipidaemia (Samson, 2014). MetS represents a risk factor for cardiovascular diseases such as heart disease and stroke, as well as type 2 diabetes mellitus (Eckel et al., 2005; Towfighi and Ovbiagele, 2008). Compared to non-MetS individuals, those with MetS exhibit a significantly increased risk of developing both cardiovascular disease and type 2 diabetes (Gonzalez-Chávez et al., 2018). In recent years, with rising income levels and changing lifestyles, the global prevalence of MetS has fluctuated between 12.5% and 31.4%, showing an upward trend (Noubiap et al., 2022). For example, the prevalence of MetS in China rose from 13.7% in 2000–01 (Gu et al., 2005) to 31.1% in 2015–17 (Yao et al., 2021), whilst in the United States, the prevalence of MetS rose from 37.6% in 2011–12%–41.8% in 2017–18 (Liang et al., 2023). MetS has progressively emerged as one of the most severe and prevalent non-communicable health threats. Current modern medical treatment prioritises lifestyle interventions such as weight reduction and dietary control, followed by pharmacotherapy primarily involving antihypertensive, antidiabetic, and lipid-lowering medications. Although studies indicate that lifestyle interventions can effectively improve MetS (Myers et al., 2019; Dayi and Ozgoren, 2022), relying solely on patient lifestyle changes is highly challenging. Moreover, no specific pharmaceutical treatment exists for MetS; management typically involves symptomatic approaches targeting individual manifestations, with limited therapeutic efficacy (Giugliano et al., 2008). Consequently, there is an urgent need for safer and more effective therapeutic strategies to address MetS.

Traditional Chinese medicine offers distinct advantages in treating MetS, as it employs a holistic approach to health by formulating customized herbal prescriptions tailored to each patient’s condition. In Traditional Chinese Medicine, Metabolic Syndrome (MetS) can be classified under categories such as “xiao ke” (thirst and wasting) and “fei man” (obesity). Although its clinical manifestations are diverse, the primary pathogenesis involves spleen deficiency with excessive dampness and the accumulation of phlegm-heat. Therefore, the HLWDD formula, which has the effects of clearing heat, drying dampness, regulating qi, and resolving phlegm, is selected. Huanglian Wendan Decoction (HDWDD) first appeared in the Qing dynasty text Six Causes and Their Differentiation (1868), derived from the Song dynasty work Three Causes and Their Ultimate Unity: Treatise on Diseases and Prescriptions (1,174), which added Coptis rhizome to the original Wendan Decoction. Its composition comprises Coptidis rhizome, Pinelliae rhizoma, Bambusae caulis in taenias, Aurantii fructus immaturus, Citri reticulatae pericarpium, Glycyrrhizae radix et rhizoma, Poria cocos, Zingiberis rhizoma recens, and Jujubae fructus. According to Traditional Chinese Medicine theory, in the HLWDD formula, Coptidis rhizome clears heat and dries dampness; Pinelliae Rhizoma dries dampness, resolves phlegm, descends rebellious qi, and harmonises the stomach; Bambusae Caulis in Taenias clears heat and resolves phlegm; and Aurantii Fructus Immaturus regulates qi and reduces inflammation - as the saying goes, “when qi flows smoothly, phlegm dissipates”. Citri Reticulatae Pericarpium regulates qi and strengthens the spleen, while also drying dampness and resolving phlegm; Poria cocos strengthens the spleen and drains dampness; Zingiberis Rhizoma Recens harmonises the stomach and resolves phlegm; Jujubae Fructus tonifies the middle and boosts qi; and Glycyrrhizae Radix et Rhizoma harmonises the actions of the other herbs. When these nine Chinese medicinal herbs are used together, they achieve the effects of clearing heat, drying dampness, regulating qi, and resolving phlegm. Modern pharmacological studies indicate that the active compounds responsible for the therapeutic effects of Coptidis rhizome and Pinelliae rhizoma in HLWDD against MS include quercetin, baicalin, β-sitosterol, stigmasterol, and other bioactive components (Wang et al., 2022). The extract from Bambusae Caulis in Taenias - pentacyclic triterpenoids (formulation code EZR 2002) - exhibits significant lipid-lowering and antihypertensive effects (Huang, 2015). Animal studies indicate that HDWDD can alleviate insulin resistance and regulate glucose and lipid metabolism (Liu et al., 2021; Pang and Li, 2019; Deng, 2012), and prevent endothelial dysfunction in MetS target organs (Guan and Wang, 2015). Additionally, HDWDD has demonstrated efficacy in treating diabetes complicated by cerebral infarction (Wang et al., 2015) and chronic renal failure (Li et al., 2019). Although clinical randomised controlled trials combining HLWDD with conventional MetS treatments have yielded considerable results in recent years, a systematic review and meta-analysis remain lacking. This study therefore comprehensively and systematically reviewed RCTs examining the use of HLWDD as an adjunct to standard treatment for MetS, to assess the efficacy and safety of HLWDD as an adjunct to standard treatment for MetS, thereby providing an effective and safe treatment option for clinical practice.

## 2 Methods and materials

### 2.1 Protocol registration database and search strategies

This systematic review and meta-analysis adheres to the guidelines of the Preferred Reporting Items for Systematic Reviews and Meta-Analyses (PRISMA) (Moher et al., 2009; Page et al., 2021). The meta-analysis was registered on the PROSPERO platform (CRD420251151304).

### 2.2 Search strategy

Searches were conducted in English-language databases (PubMed, Web of Science, Embase, Cochrane Library) and Chinese-language databases (Chinese National Knowledge Infrastructure, WanFang, Chinese Scientific Journal Database, and Chinese BioMedical Literature Database) from their inception to 1 October 2025. Search terms included Huanglian Wendan Decoction, Wendan Decoction, metabolic syndrome, and randomised controlled trials, with adjustments made according to each database’s characteristics. No restrictions were applied regarding country, language, or publication date (Supplementary Material: Search strategies).

### 2.3 Inclusion and exclusion criteria

#### 2.3.1 Inclusion criteria

(1) Randomised controlled trial of HLWDD as an adjunct to conventional treatment for MetS; (2) Subjects meeting international diagnostic criteria for MetS, with no restrictions on ethnicity, nationality, language, age, or disease duration. (3) Treatment group: HDWDD or modified HLWDD as an adjunct to conventional therapy, with no restrictions on herbal medicine dosage. Control group: conventional therapy alone. Conventional treatment comprises lifestyle interventions (exercise-induced weight loss, dietary management) and symptomatic supportive pharmacotherapy (antihypertensives, antidiabetics, or lipid-lowering agents), with no restrictions on drug type or formulation. Conventional treatment in the treatment group is identical to that in the control group. (4) Outcome measures: 1) Primary outcome measures: Obesity: waist circumference (WC), body mass index (BMI); Blood pressure: systolic blood pressure (SBP), diastolic blood pressure (DBP); Blood glucose: fasting plasma glucose (FPG), 2-h postprandial glucose (2 hPG), glycated haemoglobin (HbA1c); Lipids: Low-density lipoprotein cholesterol (LDL-C), Triglycerides (TG), High-density lipoprotein cholesterol (HDL-C). 2) Secondary outcome measures: Homeostasis model assessment of insulin resistance (HOMA-IR), Safety indicators.

#### 2.3.2 Exclusion criteria

(1) Non-randomised controlled trials (RCTs), including literature reviews, animal studies, and case reports. (2) Literature with missing or unclear data. (3) Studies involving patients who had undergone surgery, acupuncture, combined acupuncture and medication, or acupoint plaster application. (4) Treatment groups not utilising HLWDD in conjunction with conventional therapy. (5) Literature lacking primary or secondary outcome measures.

### 2.4 Data extraction

Two researchers (T.P.J and J.T.D) independently conducted literature searches and screening according to the inclusion and exclusion criteria. After removing duplicate records, preliminary screening was performed based on titles and abstracts, followed by a secondary screening through full-text review. Where discrepancies arose, consensus was reached through discussion between the two researchers (T.P.J and J.T.D). Persistent disagreements were resolved by a third researcher (Y.X.Z), who extracted data for assessment and review. Key data extracted from the literature included: first author, publication year, country, sample size, age, intervention measures, treatment duration, and outcome measures.

### 2.5 Literature quality assessment

Two researchers, T.P.J and J.T.D, independently assessed the risk of bias using the Cochrane risk of bias tool. The assessment encompassed random sequence generation, allocation concealment, blinding, incomplete outcome data, selective reporting, and other sources of bias.

### 2.6 Statistical analysis

The sample size of this study was obtained by direct pooled statistical analysis of the case numbers from the included literature, with no additional sample size estimation performed. Statistical analyses were conducted using RevMan 5.3 software (Cochrane Collaboration, Oxford, United Kingdom) and Stata 17.0 software (StataCorp, College Station, USA). For continuous variables, the mean difference (MD) with a 95% confidence interval (CI) was used, as measurement methods and units were identical. For dichotomous variables, the odds ratio (OR) with a 95% CI was employed due to low event rates. Heterogeneity was assessed using χ^2^ tests and I^2^ tests. If p > 0.1 and I^2^ < 50%, homogeneity was assumed, warranting a fixed-effects model. Conversely, p ≤ 0.1 and I^2^ ≥ 50% indicated heterogeneity, necessitating a random-effects model. If there is significant heterogeneity between studies, cross-validation sensitivity analyses will be conducted using RevMan 5.3 and Stata 17.0 software. The aim is to assess the robustness and reliability of the pooled results, minimise calculation bias arising from reliance on a single statistical software package, and enhance the credibility of the meta-analysis conclusions. Subgroup analyses or meta-regression analyses were performed based on duration, age, random sequence generation, and number to explore potential sources of heterogeneity. If ≥ 10 studies included the observed outcome measure, a funnel plot and Egger’s test are employed to assess the potential risk of bias. A p < 0.05 indicates possible publication bias, requiring trimming to verify; conversely, no publication bias is present.

## 3 Results

### 3.1 Search results

**FIGURE 1:**
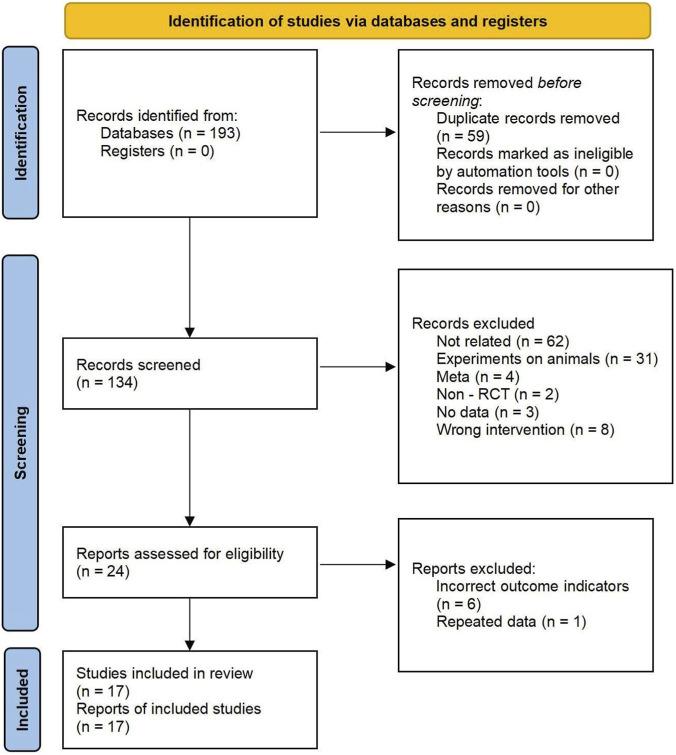
Filtering flowchart. PRISMA 2020 flow diagram for new systematic reviews which included searches of databases and registers only. Source: Page MJ, et al. BMJ 2021;372:n71. doi: 10.1136/bmj.n71. This work is licensed under CC BY 4.0. To view a copy of this license, visit https://creativecommons.org/licenses/by/4.0/.

A total of 193 articles were retrieved from Chinese and English databases, with 59 duplicate publications excluded. Review of titles and abstracts led to the exclusion of 110 articles. After full-text screening, a further seven publications were excluded: six due to erroneous outcome measures and one containing duplicated data. Ultimately, 17 studies meeting the inclusion criteria were incorporated into the meta-analysis, as detailed in Figure 1.

### 3.2 Research characteristics

A total of 17 studies were ultimately included (see Table 1; Supplementary Material S1), involving 1,503 patients with metabolic syndrome. The treatment group comprised 755 cases, while the control group comprised 748 cases. The treatment group received HDWDD or modified HLWDD in addition to conventional drug therapy, with the specific herbal metabolites of HDWDD detailed in Table 2. The control group received standard drug therapy alone.

**TABLE 1:** Characteristics of the included studies.

First author (year)	Randomization	Country	Number (T/C)	Patient age (years) (T)	Patient age (years) (C)	Interventions (T)	Interventions (C)	Duration	Outcome
Bai (2015)	Random	China	30/30	57.15 ± 10.33	55.63 ± 10.24	C + HLWDD	Lifestyle intervention + Conventional hypoglycemic drugs, lipid-regulating drugs, and antihypertensive drugs	12 weeks	①②③④⑤⑥⑧⑨⑩⑫
Ding (2025)	Random number table	China	75/75	56.57 ± 2.12	56.92 ± 2.05	C + modified HLWDD	Lifestyle intervention + metformin hydrochloride tablets po 500 mg tid	28 days = 4 weeks	⑤⑦⑧⑨⑩
Dong et al. (2025)	Random number table	China	90/88	58.91 ± 8.79	61.46 ± 7.62	C + modified HLWDD	Lifestyle intervention + Atorvastatin po 10 mg qd + valsartan capsules po 80 mg qd + metformin hydrochloride tablets po 0.5 g tid	3 months = 12 weeks	①②③④⑤⑥⑦⑧⑨⑩⑪⑫
Han et al. (2025)	Random number table	China	41/41	60.65 ± 11.54	55.43 ± 9.84	C + modified HLWDD	Lifestyle intervention + atorvastatin po 20 mg qd + valsartan capsules po 80 mg qd + metformin po 0.5 g tid	8 weeks	①③④⑤⑥⑦⑧⑨⑩⑪⑫
Jiang et al. (2018)	Random number table	China	24/24	50.17 ± 2.65	51.49 ± 3.14	C + modified HLWDD	Lifestyle intervention + metformin hydrochloride tablets po 500 mg tid	3 weeks	②⑤⑥⑦⑧⑨⑩⑪⑫
Jin (2021)	Random	China	31/31	49.58 ± 9.70	50.61 ± 6.17	C + modified HLWDD	Lifestyle intervention + conventional hypoglycemic drugs, lipid-regulating drugs, and antihypertensive drugs	12 weeks	①②③④⑤⑥⑦⑧⑨⑩⑪ ⑫
Liu et al. (2016)	Random number table	China	42/42	43.67 ± 15.09	44.23 ± 14.71	C + modified HLWDD	Lifestyle intervention + conventional hypoglycemic drugs, lipid-regulating drugs, and antihypertensive drugs	4 weeks	①②③④⑤⑥⑧⑨⑩⑪
Ma (2020)	Random number table	China	100/100	42.47 ± 10.62	42.28 ± 10.56	C + modified HLWDD	Lifestyle intervention + Metformin po 2pieces Bid + glipizide tablets po 2pieces bid	4 weeks	⑥⑦⑨⑩
Meng (2012)	Random	China	30/30	49.13 ± 7.09	49.37 ± 7.52	C + modified HLWDD	Lifestyle intervention + Conventional hypoglycemic drugs, lipid-regulating drugs, and antihypertensive drugs	4 weeks	①②③④⑤⑥⑧⑨⑩⑫
Sui and Liu (2015)	Random	China	20/19	43.2 ± 8.1	45.1 ± 7.3	C + HLWDD	Lifestyle intervention + Fosinopril + Metformin + atorvastatin	4 weeks	②③⑤⑧⑨⑩
Wang and Song (2012)	Random	China	60/60	42 ± 5	41 ± 7	C + modified HLWDD	Lifestyle intervention + Enalapril + simvastatin tablets	3 months = 12 weeks	③④⑤⑥⑧⑨⑩⑫
Wang et al. (2022)	Random number table	China	34/34	52.3 ± 11.8	52.4 ± 12.0	C + modified HLWDD	Lifestyle intervention + Atorvastatin po 10 mg qd + valsartan capsules po 80 mg qd + metformin po 0.5 g tid	8 weeks	②③④⑤⑥⑧⑨⑩⑪⑫
Wang (2023)	Random number table	China	34/34	51.71 ± 11.10	50.15 ± 9.92	C + modified HLWDD	Lifestyle intervention + Atorvastatin po 20 qd + valsartan capsules po 80 mg qd + metformin po 0.5 g tid	4 weeks	①②③④⑤⑥⑧⑨⑩⑪⑫
Xian and Chen (2021)	Unclear	China	50/50	50.39 ± 2.26	50.36 ± 2.20	C + modified HLWDD	Atorvastatin po 25 mg qd	4 weeks	⑤⑥⑦⑨
Xu (2008)	Unclear	China	30/26	38 (1–55)	34 (25–60)	C + modified HLWDD	Lifestyle intervention + Metformin + angiotensin-converting enzyme inhibitors + fenofibrate capsules	1 month = 4 weeks	⑤⑧⑩
Xu (2009)	Random	China	30/30	49.27 ± 8.47	49.43 ± 7.47	C + HLWDD	Lifestyle intervention + atorvastatin po 10 mg qd + perindopril tablets po 4 mg qd + metformin po 0.25 g tid	4 weeks	②③④⑤⑥⑧⑨⑫
Yang et al. (2025)	Random number table	China	34/34	52.4 ± 11.6	54.5 ± 13.0	C + modified HLWDD	Lifestyle intervention + Atorvastatin po 10 mg qd + valsartan capsules po 80 mg qd + metformin po 0.5 g tid	6 weeks	②③④⑤⑧⑨⑩⑪⑫

T: treatment group, C: control group, HLWDD: huanglian wendan decoction, po: oral administration, qd: Once a day, bid: Twice a day, tid: Three times a day, ①: WC, ②: BMI, ③: SBP, ④: DBP, ⑤: FPG, ⑥: 2 hPG, ⑦: HbA1c, ⑧: TG, ⑨: LDL-C, ⑩: HDL-C, ⑪: HOMA-IR, ⑫: Safety indicators.

**TABLE 2:** HLWDD’s herbal composition.

Scientific name	Family	Species name	Part(s) of drug used
Coptis chinensis franch	Ranunculaceae	Coptidis rhizome	Dried rhizome
Pinellia ternata (thunb.) makino	Araceae	Pinelliae rhizoma	Dried tuber
Bambusa tuldoides munro	Poaceae	Bambusae caulis in taenias	Dried middle shavings of stem
Citrus × aurantium L	Rutaceae	Aurantii fructus immaturus	Dried young fruit
Citrus reticulata blanco	Rutaceae	Citri reticulatae pericarpium	Dried pericarp of the ripe fruit
Glycyrrhiza glabra L	Rutaceae	Glycyrrhizae radix et rhizoma	Dried pericarp of the ripe fruit
Poria cocos (schw.) wolf	Polyporaceae	Poria cocos	Dried sclerotium
Zingiber officinale roscoe	Zingiberaceae	Zingiberis rhizoma recens	Dried rhizome
Ziziphus jujuba mill	Rhamnaceae	Jujubae fructus	Dried ripe fruit

### 3.3 Assessment of the risk of bias

A systematic assessment of the quality of 17 randomised controlled trials (RCTs) was conducted using the quality assessment tools recommended by the Cochrane Manual. Regarding random sequence generation, nine studies (Ding, 2025; Dong et al., 2025; Han et al., 2025; Jiang et al., 2018; Liu et al., 2016; Ma, 2020; Wang et al., 2022; Wang, 2023; Yang et al., 2025) employed random number tables and were rated as low risk; eight studies (Bai, 2015; Jin, 2021; Meng, 2012; Sui and Liu, 2015; Wang and Song, 2012; Xian and Chen, 2021; Xu, 2008; Xu, 2009) documented unclear randomisation methods and were rated as having unclear risk. One study (Dong et al., 2025) employed sealed envelopes, and one (Han et al., 2025) used sequential numbering; thus, their allocation procedures were rated as low risk. The remaining studies did not mention allocation procedures or methods of constraint and were therefore rated as having unclear risk.

**FIGURE 2:**
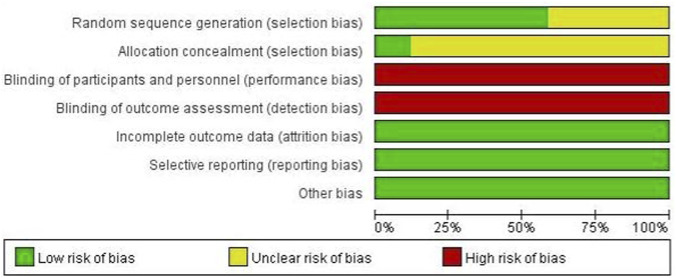
Risk of bias graph.

**FIGURE 3:**
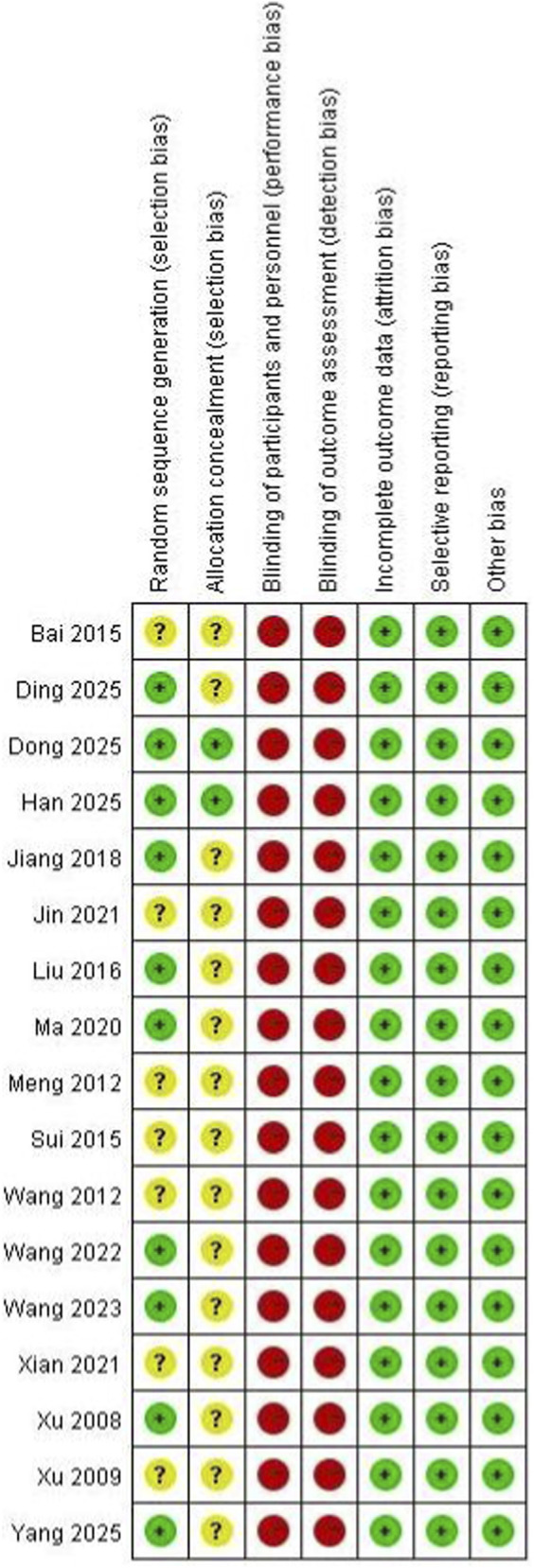
Risk of bias summary.

In blinding, 17 were not blinded to either the patients or the principal investigators and were therefore rated as high risk. Regarding completeness and selective reporting of outcome data, 17 studies showed no data deficiencies and no selective reporting of results, thus assessed as low risk. There were no other sources of bias in the included studies, so they were rated as low risk (Figures 2,3).

### 3.4 Meta-analysis results

#### 3.4.1 Primary outcome measures

##### 3.4.1.1 WC

**FIGURE 4:**
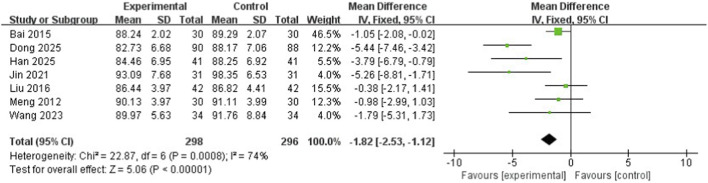
Forest plot of the WC.

Seven studies (Jin, 2021; Liu et al., 2016; Meng, 2012; Bai, 2015; Wang, 2023; Dong et al., 2025; Han et al., 2025) reported waist circumference (WC) data. Due to substantial heterogeneity (P = 0.0008, I^2^ = 74%), a random-effects model was employed. Results demonstrated a significant reduction in WC within the HLWDD + C treatment group (MD = −1.82 cm, 95% CI:−2.53 to −1.12, P < 0.00001) (Figure 4).

##### 3.4.1.2 BMI

**FIGURE 5:**
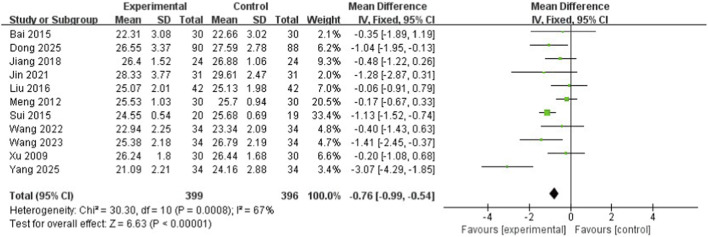
Forest plot of the BMI.

Eleven studies (Jin, 2021; Liu et al., 2016; Xu, 2009; Yang et al., 2025; Meng, 2012; Bai, 2015; Wang et al., 2022; Wang, 2023; Dong et al., 2025; Sui and Liu, 2015; Jiang et al., 2018) reported BMI. Significant heterogeneity existed across studies (P = 0.0008, I^2^ = 67%), analysed using a random-effects model. Results indicated a significant reduction in BMI within the HLWDD + C treatment group (MD = −0.76 kg/m2, 95% CI: −0.99 to −0.54, P < 0.00001) (Figure 5).

##### 3.4.1.3 SBP

**FIGURE 6:**
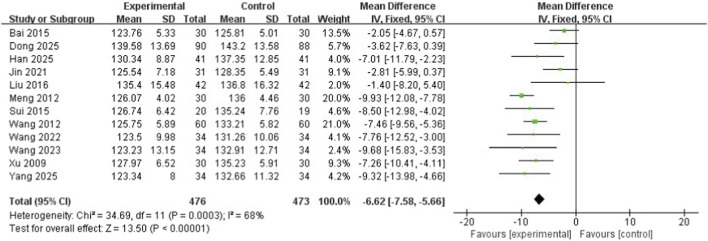
Forest plot of the SBP.

Twelve studies (Jin, 2021; Liu et al., 2016; Xu, 2009; Wang and Song, 2012; Yang et al., 2025; Meng, 2012; Bai, 2015; Wang et al., 2022; Wang, 2023; Dong et al., 2025; Sui and Liu, 2015; Han et al., 2025) addressed systolic blood pressure (SBP). Results indicated significant heterogeneity (P = 0.0003, I^2^ = 68%), necessitating a random-effects model. The meta-analysis demonstrated a significant reduction in SBP within the HLWDD + C treatment group (MD = −6.62 mmHg, 95% CI: −7.58 to −5.66, P < 0.00001) (Figure 6).

##### 3.4.1.4 DBP

**FIGURE 7:**
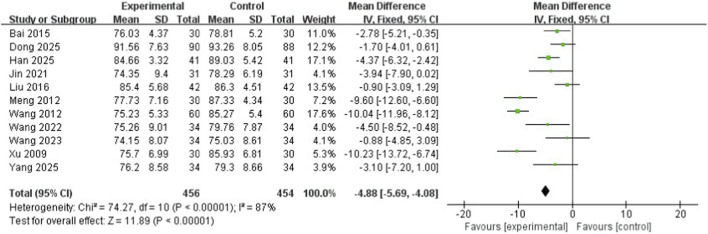
Forest plot of the DBP.

Eleven studies (Jin, 2021; Liu et al., 2016; Xu, 2009; Wang and Song, 2012; Yang et al., 2025; Meng, 2012; Bai, 2015; Wang et al., 2022; Wang, 2023; Dong et al., 2025; Han et al., 2025) addressed diastolic blood pressure (DBP). Significant heterogeneity existed among studies regarding DBP (P < 0.00001, I^2^ = 87%). Results demonstrated a statistically significant reduction in DBP within the HLWDD + C treatment group (MD = −4.88 mmHg, 95% CI: −5.69 to −4.08, P < 0.00001) (Figure 7).

##### 3.4.1.5 FPG

**FIGURE 8:**
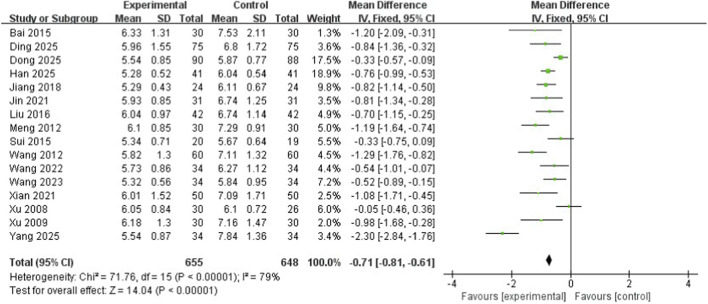
Forest plot of the FPG.

A total of 16 studies (Xian and Chen, 2021; Jin, 2021; Liu et al., 2016; Xu, 2008; Xu, 2009; Wang and Song, 2012; Yang et al., 2025; Meng, 2012; Bai, 2015; Wang et al., 2022; Wang, 2023; Dong et al., 2025; Sui and Liu, 2015; Han et al., 2025; Ding, 2025; Jiang et al., 2018) reported FPG. Heterogeneity testing revealed significant heterogeneity (P < 0.00001, I^2^ = 79%), necessitating the use of a random-effects model. Results demonstrated a significant reduction in FPG within the HLWDD + C treatment group (MD = −0.71 mmol/L, 95% CI: −0.81 to −0.61, P < 0.00001) (Figure 8).

##### 3.4.1.6 2 hPG

**FIGURE 9:**
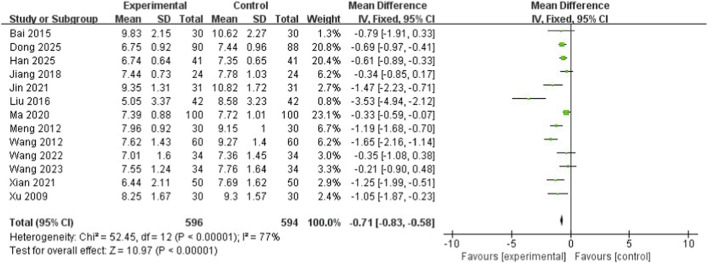
Forest plot of the 2 hPG

Thirteen studies (Xian and Chen, 2021; Jin, 2021; Liu et al., 2016; Xu, 2009; Wang and Song, 2012; Meng, 2012; Ma, 2020; Bai, 2015; Wang et al., 2022; Wang, 2023; Dong et al., 2025; Han et al., 2025; Jiang et al., 2018) concerning 2-h postprandial glucose (2 hPG). Significant heterogeneity existed among studies regarding 2 hPG (P < 0.00001, I^2^ = 77%). Results indicated a significant reduction in 2 hPG in the HLWDD + C treatment group (MD = −0.71 mmol/L, 95% CI: −0.83 to −0.58, P < 0.00001) (Figure 9).

##### 3.4.1.7 HbA1c

**FIGURE 10:**
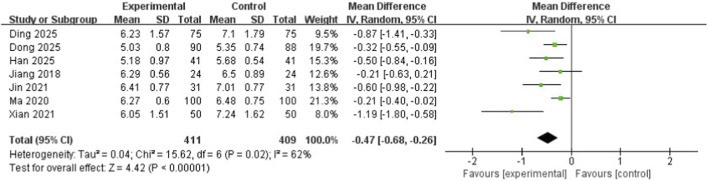
Forest plot of the HbA1c.

Seven studies (Xian and Chen, 2021; Jin, 2021; Ma, 2020; Dong et al., 2025; Han et al., 2025; Ding, 2025; Jiang et al., 2018) examined HbA1c. Significant heterogeneity existed between studies regarding HbA1c (P = 0.02, I^2^ = 62%). Results demonstrated a significant reduction in HbA1c within the HLWDD + C treatment group (MD = −0.47%, 95% CI: −0.68 to −0.26, P < 0.00001) (Figure 10).

##### 3.4.1.8 TG

**FIGURE 11:**
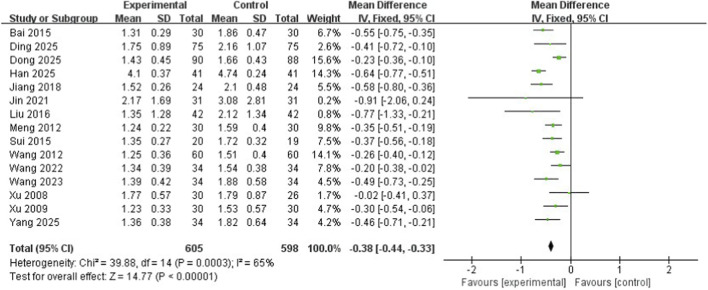
Forest plot of the TG.

Fifteen studies (Jin, 2021; Liu et al., 2016; Xu, 2008; Xu, 2009; Wang and Song, 2012; Yang et al., 2025; Meng, 2012; Bai, 2015; Wang et al., 2022; Wang, 2023; Dong et al., 2025; Sui and Liu, 2015; Han et al., 2025; Ding, 2025; Jiang et al., 2018) concerning TG. Significant heterogeneity existed among studies regarding TG (P = 0.0003, I^2^ = 65%). Results demonstrated a significant reduction in TG in the HLWDD + C treatment group (MD = −0.38 mmol/L, 95% CI: −0.44 to −0.33, P < 0.00001) (Figure 11).

##### 3.4.1.9 LDL-C

**FIGURE 12:**
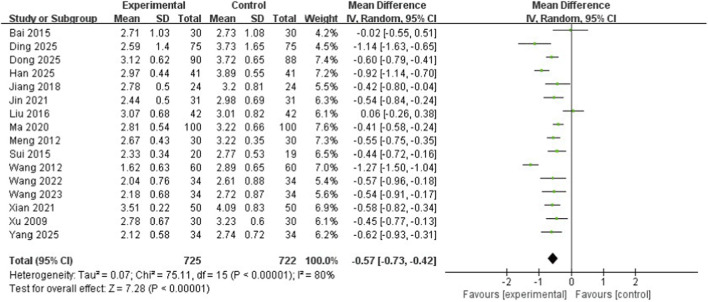
Forest plot of the LDL-C.

Sixteen studies (Xian and Chen, 2021; Jin, 2021; Liu et al., 2016; Xu, 2009; Wang and Song, 2012; Yang et al., 2025; Meng, 2012; Ma, 2020; Bai, 2015; Wang et al., 2022; Wang, 2023; Dong et al., 2025; Sui and Liu, 2015; Han et al., 2025; Ding, 2025; Jiang et al., 2018) concerning LDL-C. Significant heterogeneity existed among studies regarding LDL-C (P < 0.00001, I^2^ = 80%). Results demonstrated a significant reduction in LDL-C in the HLWDD + C treatment group (MD = −0.57 mmol/L, 95% CI: −0.73 to −0.42, P < 0.00001) (Figure 12).

##### 3.4.1.10 HDL-C

**FIGURE 13:**
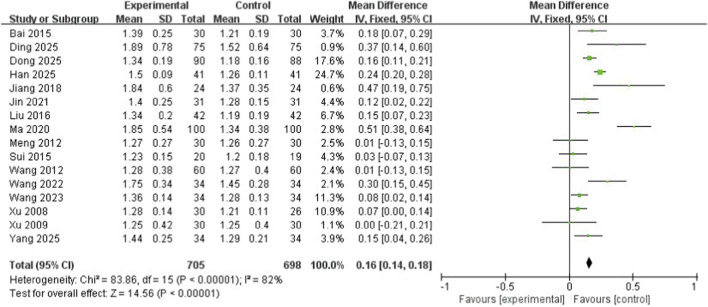
Forest plot of the HDL-C.

Sixteen studies (Jin, 2021; Liu et al., 2016; Xu, 2008; Xu, 2009; Wang and Song, 2012; Yang et al., 2025; Meng, 2012; Ma, 2020; Bai, 2015; Wang et al., 2022; Wang, 2023; Dong et al., 2025; Sui and Liu, 2015; Han et al., 2025; Ding, 2025; Jiang et al., 2018) concerning HDL-C. Significant heterogeneity existed among studies regarding HDL-C (P < 0.00001, I^2^ = 82%). Results indicated a significant reduction in HDL-C in the conventional treatment control group (MD = 0.16 mmol/L, 95% CI (0.14, 0.18), P < 0.00001) (Figure 13).

#### 3.4.2 Secondary outcome measures

##### 3.4.2.1 HOMA-IR

**FIGURE 14:**
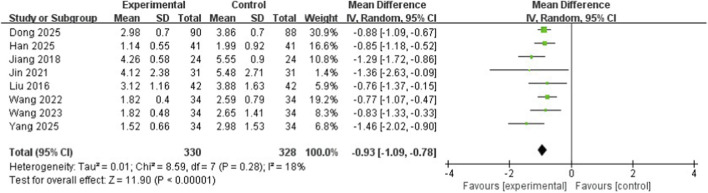
Forest plot of the HOMA-IR.

Eight studies (Jin, 2021; Liu et al., 2016; Yang et al., 2025; Wang et al., 2022; Wang, 2023; Dong et al., 2025; Han et al., 2025; Jiang et al., 2018) addressed HOMA-IR. No significant heterogeneity was observed between studies (P = 0.28, I^2^ = 18%). Results demonstrated a significant reduction in HOMA-IR within the HLWDD + C treatment group (MD = −0.93, 95% CI: −1.09 to −0.78, P < 0.00001) (Figure 14).

##### 3.4.2.2 Safety indicators

**FIGURE 15:**
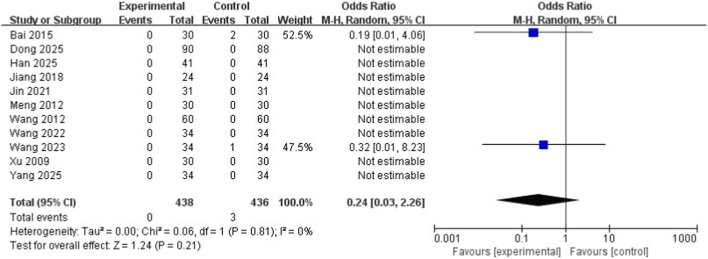
Forest plot of the Safety indicators.

Eleven studies addressed safety outcomes. Nine studies (Jin, 2021; Xu, 2009; Wang and Song, 2012; Yang et al., 2025; Meng, 2012; Wang et al., 2022; Dong et al., 2025; Han et al., 2025; Jiang et al., 2018) reported no adverse events. Bai 2015 documented two cases of mild abdominal distension following oral administration of Baidangping, which proved tolerable without requiring discontinuation of treatment. Wang 2023 noted one case of mild abdominal distension in the control group, which resolved spontaneously without specific intervention. No significant heterogeneity existed among studies regarding safety indicators (P = 0.81, I^2^ = 0%). Results showed no statistically significant difference (OR = 0.24, 95% CI: 0.03 to 2.26, P = 0.21) (Figure 15).

#### 3.4.3 Sensitivity analysis

Sensitivity analyses for WC, BMI, SBP, DBP, FPG, 2 hPG, HbA1c, TG, LDL-C, and HDL-C were conducted using RevMan 5.3 and Stata 17.0 software. Results indicated that WC demonstrated homogeneity after excluding Dong et al., 2025 (P = 0.12, I^2^ = 43%), with statistically significant differences (MD = −1.32, 95% CI (−2.07, −0.56), P = 0.0006), reflecting relatively stable outcomes. BMI demonstrated homogeneity after excluding Yang et al., 2025 (P = 0.07, I^2^ = 44%), with a statistically significant difference in results (MD = −0.68, 95% CI (−0.91, −0.45), P < 0.00001), indicating relatively stable findings; HbA1c showed homogeneity after excluding Xian and Chen, 2021 (P = 0.13, I^2^ = 42%), with statistically significant differences (MD = −0.39, 95% CI (−0.55, −0.22), P < 0.00001), and relatively stable results; TG showed homogeneity after excluding Han et al., 2025 (P = 0.03, I^2^ = 46%), with statistically significant differences (MD = −0.34, 95% CI (−0.40, −0.29), P < 0.00001), and relatively stable results. Results indicate that Dong et al., 2025, Yang et al., 2025, Xian and Chen, 2021; Han et al., 2025 may be the primary sources of statistical heterogeneity for WC, BMI, HbA1c, and TG, respectively. Sensitivity analyses for SBP, DBP, FPG, 2 hPG, LDL-C, and HDL-C demonstrated robustness. Further investigation of heterogeneity sources via subgroup analysis or meta-regression analysis remains warranted (Supplementary Material S2).

#### 3.4.4 Subgroup analysis

Subgroup analysis of SBP, DBP, FPG, 2 hPG, LDL-C, and HDL-C was conducted using Stata 17.0 software. Based on information from the included literature, we pre-specified factors potentially influencing heterogeneity and grouped studies according to the following four criteria: (1) Random sequence generation: Group 1 used random number tables; Group 2 employed randomisation; (2) Sample size: ≤40 subjects in Group 1, >40 subjects in Group 2 (where the treatment group has ≤40 subjects and the control group has >40 subjects, the treatment group size takes precedence); (3) Age ≤50 years in Group 1, >50 years in Group 2 (where the treatment group has ≤50 years and the control group has >50 years, the treatment group age takes precedence); (4) Duration ≤4 weeks: Group 1; >4 weeks: Group 2. When subgroup analysis yields p > 0.1 and I^2^ < 50% within each group, indicating significantly reduced heterogeneity for that factor, it is deemed a source of heterogeneity. Otherwise, the factor is not considered a source of heterogeneity.

Subgroup analysis results for SBP indicated that random sequence generation (Group 1: I^2^ = 26.4%, P = 0.236; Group 2: I^2^ = 82.0%, P = 0.000), number (Group 1: I^2^ = 76.3%, P = 0.000; Group 2: I^2^ = 39.6%, P = 0.174), age (Group 1: I^2^ = 69.8%, P = 0.005; Group 2: I^2^ = 60.3%, P = 0.027), and duration (Group 1: I^2^ = 39.3%, P = 0.159; Group 2: I^2^ = 65.1%, P = 0.009). Heterogeneity within groups did not decrease concurrently for these factors; thus, they cannot currently be considered sources of SBP heterogeneity. DBP subgroup analysis revealed that random sequence generation (Group 1: I^2^ = 35.1%, P = 0.173; Group 2: I^2^ = 85.9%, P = 0.000), number of subjects (Group 1: I^2^ = 77.0%, P = 0.000; Group 2: I^2^ = 93.7%, P = 0.000), and age (Group 1: I^2^ = 91.6%, P = 0.000; Group 2: I^2^ = 0.0%, P = 0.443), duration (Group 1: I^2^ = 91.4%, P = 0.000; Group 2: I^2^ = 0.0%, P = 0.772). Heterogeneity within groups did not decrease simultaneously for any combination of these four factors; thus, they cannot currently be considered sources of DBP heterogeneity. FPG subgroup analysis revealed that random sequence generation (Group 1: I^2^ = 84.5%, P = 0.000; Group 2: I^2^ = 73.5%, P = 0.000), number of subjects (Group 1: I^2^ = 83.3%, P = 0.000; Group 2: I^2^ = 70.9%, P = 0.004), age (Group 1: I^2^ = 75.2%, P = 0.000; Group 2: I^2^ = 83.2%, P = 0.000), and duration (Group 1: I^2^ = 61.5%, P = 0.008; Group 2: I^2^ = 88.0%, P = 0.000). Heterogeneity within groups did not decrease concurrently for any combination of these factors, thus ruling them out as sources of FPG heterogeneity. Subgroup analysis for 2 hPG revealed that random sequence generation (Group 1: I^2^ = 73.7%, P = 0.001; Group 2: I^2^ = 0.0%, P = 0.643), number of subjects (Group 1: I^2^ = 54.4%, P = 0.041; Group 2: I^2^ = 87.2%, P = 0.000), and age (Group 1: I^2^ = 88.4%, P = 0.000; Group 2: I^2^ = 3.1%, P = 0.402), duration (Group 1: I^2^ = 81.6%, P = 0.000; Group 2: I^2^ = 71.7%, P = 0.003). Heterogeneity within groups did not decrease simultaneously for any combination of these factors. Therefore, these four factors cannot currently be considered sources of heterogeneity in 2 hPG.LDL-C subgroup analysis results indicate that random sequence generation (Group 1: I^2^ = 76.7%, P = 0.000; Group 2: I^2^ = 84.6%, P = 0.000), number (Group 1: I^2^ = 0.0%, P = 0.800; Group 2: I^2^ = 90.8%, P = 0.000), age (Group 1: I^2^ = 89.2%, P = 0.000; Group 2: I^2^ = 54.9%, P = 0.023), and duration (Group 1: I^2^ = 59.5%, P = 0.011; Group 2: I^2^ = 82.9%, P = 0.000). Heterogeneity within groups did not decrease concurrently for any combination of these factors, thus ruling them out as sources of LDL-C heterogeneity. Subgroup analysis for HDL-C revealed random sequence generation (Group 1: I^2^ = 84.2%, P = 0.000; Group 2: I^2^ = 14.6%, P = 0.319), number of subjects (Group 1: I^2^ = 59.4%, P = 0.008; Group 2: I^2^ = 86.8%, P = 0.000), and age (Group 1: I^2^ = 84.9%, P = 0.000; Group 2: I^2^ = 73.6%, P = 0.000), duration (Group 1: I^2^ = 85.8%, P = 0.000; Group 2: I^2^ = 65.1%, P = 0.009). Heterogeneity within groups did not decrease simultaneously for any combination of these four factors; thus, they cannot currently be considered sources of HDL-C heterogeneity. Subgroup analyses for SBP, DBP, FPG, 2 hPG, LDL-C, and HDL-C failed to identify sources of heterogeneity. Further investigation via meta-regression analysis is required to determine these sources (Supplementary Material S2-S8).

#### 3.4.5 Meta regression analysis

Employing Stata 17.0 software, meta-regression analysis was conducted on SBP, DBP, FPG, 2 hPG, LDL-C, and HDL-C across four dimensions: random sequence generation, number, age, and duration. Where P < 0.05 indicated significant differences in meta-regression analysis results, the respective factor was deemed a source of heterogeneity.

Meta-regression analysis results for SBP indicated that random sequence generation (p = 0.065), number (p = 0.227), age (p = 0.180), and duration (p = 0.250) were not sources of SBP heterogeneity. Meta-regression analysis results for DBP indicated that random sequence generation (p = 0.005) significantly influenced DBP and may be a source of DBP heterogeneity. Number (p = 0.859), age (p = 0.050), and duration (p = 0.685) were not sources of DBP heterogeneity. Meta-regression analysis for FPG indicated that random sequence generation (p = 0.962), number (p = 0.910), age (p = 0.604), and duration (p = 0.278) were not sources of FPG heterogeneity. Meta-regression analysis for 2 hPG indicated that random sequence generation (p = 0.139), number (p = 0.216), age (p = 0.066), and duration (p = 0.556) were not sources of 2 hPG heterogeneity. Meta-regression analysis for LDL-C revealed that random sequence generation (p = 0.429), number (p = 0.412), age (p = 0.726), and duration (p = 0.233) were not sources of heterogeneity for 2 hPG. Meta-regression analysis for HDL-C indicated that random sequence generation (p = 0.004) and age (p = 0.034) significantly influenced DBP, potentially contributing to DBP heterogeneity. Number (p = 0.167) and duration (p = 0.211) were not sources of DBP heterogeneity (Supplementary Material S9-S24).

#### 3.4.6 Publication bias

Using Stata 17.0 software, conduct funnel plots and Egger’s tests for BMI, SBP, DBP, FPG, 2 hPG, TG, LDL-C, and HDL-C indicators with over ten studies each. Should the funnel plot exhibit symmetry or Egger’s test yield a P-value >0.05, this indicates no potential publication bias. Conversely, an asymmetric funnel plot coupled with a P-value <0.05 in Egger’s test suggests potential publication bias, necessitating further analysis using trim-and-trim methods.

**FIGURE 16:**
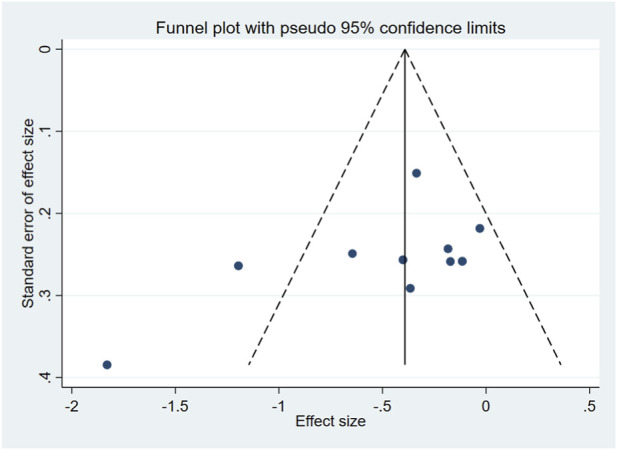
Publication bias funnel plot of BMI.

**FIGURE 17:**
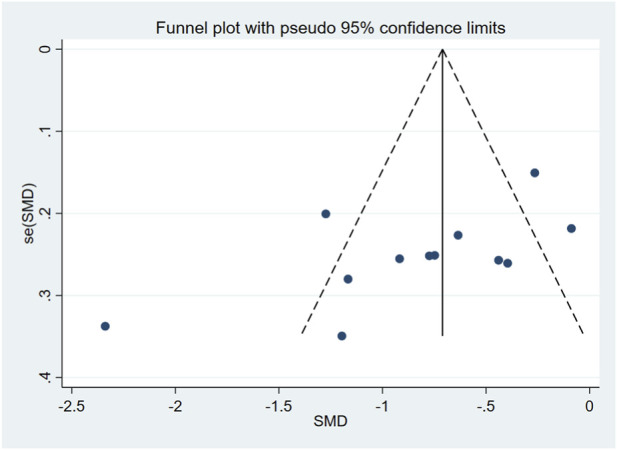
Publication bias funnel plot of SBP.

**FIGURE 18:**
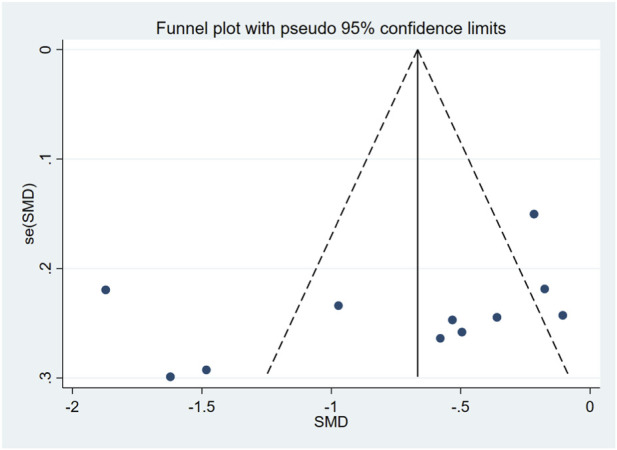
Publication bias funnel plot of DBP.

**FIGURE 19:**
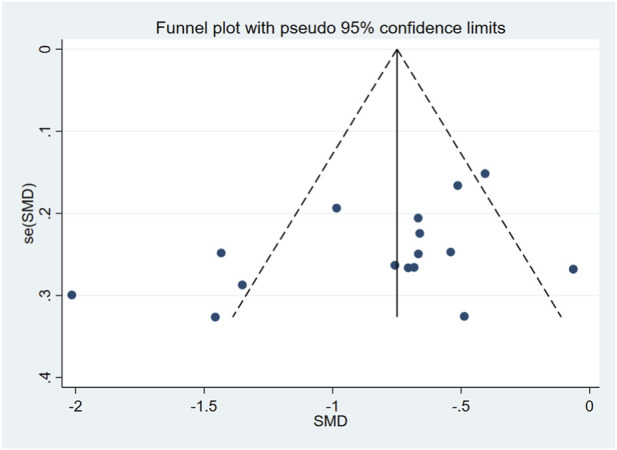
Publication bias funnel plot of FBG.

**FIGURE 20:**
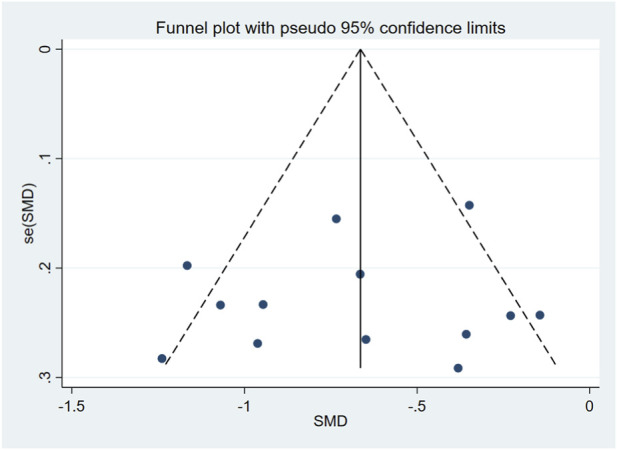
Publication bias funnel plot of 2 hPG

**FIGURE 21:**
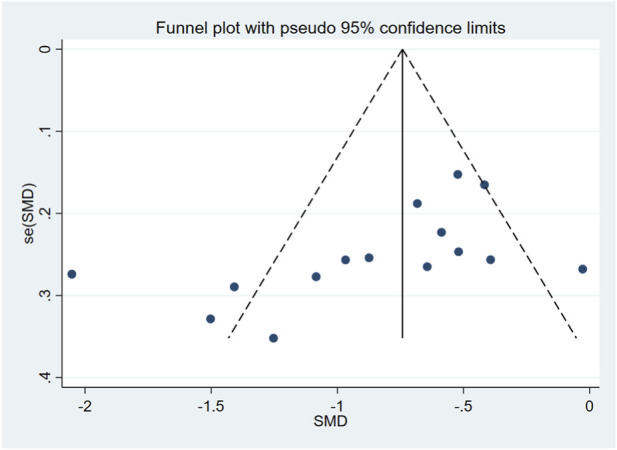
Publication bias funnel plot of TG.

**FIGURE 22:**
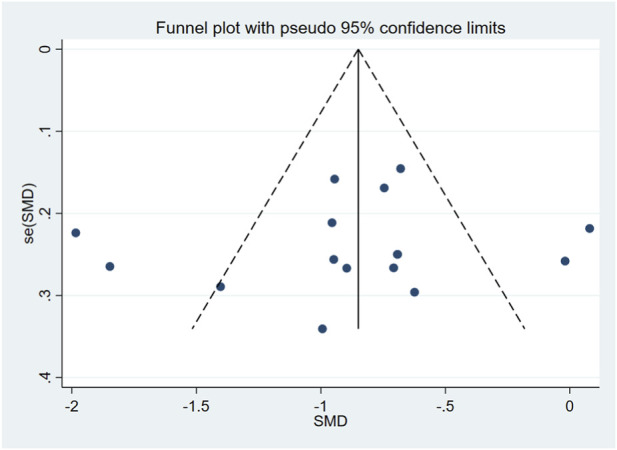
Publication bias funnel plot of LDL-C.

**FIGURE 23:**
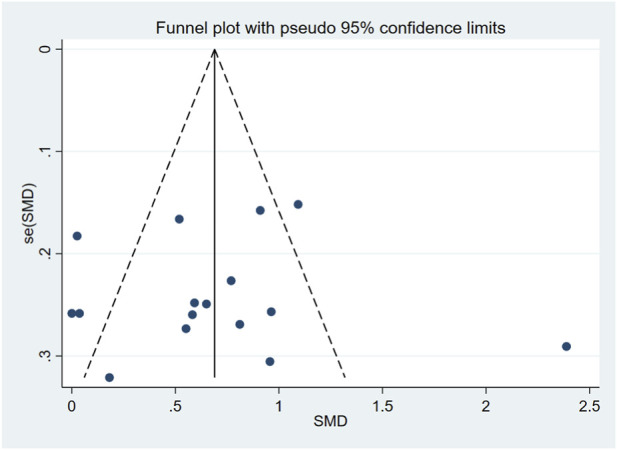
Publication bias funnel plot of HDL-C.

The results indicate that the funnel plot for BMI exhibits an asymmetric distribution (Figure 16), yet Egger’s test reveals no statistically significant difference (P = 0.164), suggesting no apparent publication bias for BMI. The funnel plot for SBP exhibited an asymmetric distribution (Figure 17), yet Egger’s test revealed no statistically significant difference (P = 0.041), suggesting potential publication bias for SBP. After applying the trimming method, no studies were added or removed, indicating that trimming failed to identify potential bias in SBP (Supplementary Material S27). The funnel plot for DBP exhibited asymmetry (Figure 18), yet Egger’s test revealed no statistical significance (P = 0.824), indicating no apparent publication bias for DBP. The funnel plot for FPG showed asymmetry (Figure 19), but Egger’s test indicated no statistical significance (P = 0.066), suggesting no evident publication bias for FPG. The funnel plot for 2 hPG exhibited an asymmetric distribution (Figure 20), yet Egger’s test revealed no statistically significant difference (P = 0.601), indicating no apparent publication bias for 2 hPG. The TG funnel plot exhibited an asymmetric distribution (Figure 21), yet Egger’s test revealed no statistically significant difference (P = 0.032), suggesting potential publication bias for TG. After applying the trimming method, no studies were added or removed, indicating the trimming method failed to identify potential bias in TG (Supplementary Material S27). The LDL-C funnel plot exhibited broadly symmetric distribution (Figure 22), consistent with Egger’s test results (P = 0.589), indicating no apparent publication bias for LDL-C. HDL-C exhibited an asymmetric distribution (Figure 23), yet Egger’s test revealed no statistically significant difference (P = 0.912), indicating no apparent publication bias for HDL-C (Supplementary Material S25,26).

## 4 Discussion

### 4.1 Key research findings

Metabolic syndrome (MetS) is a metabolic disorder arising from insulin resistance coupled with fluctuating fatty acid levels, typically manifesting as abdominal obesity, hypertension, hyperglycaemia, and dyslipidaemia (McCracken et al., 2018). HLWDD is a classical formula in traditional Chinese medicine. In recent years, an increasing number of studies have reported favourable outcomes from HLWDD as an adjunctive therapy for MetS. To date, only one systematic review on HLWDD for MetS treatment was published in 2013, which included merely three studies and lacked clear randomisation and allocation concealment protocols (Zhu et al., 2013). This study, therefore, represents the first systematic and comprehensive investigation into the efficacy and safety of HLWDD as an adjunct to conventional MetS treatment, providing evidence-based support for its application. As the course of the disease varies from patient to patient, practitioners of Traditional Chinese Medicine (TCM) analyse each case individually and adjust the HLWDD formula accordingly; this embodies the TCM principle of tailoring treatment to the individual. Whether using the standard HLWDD formula or its modified versions, the core herbal ingredients remain consistent with those recorded in ancient texts; therefore, both the standard HLWDD formula and its modified versions are included in our study. Through rigorous and comprehensive searching and screening, 17 studies were ultimately included and systematically evaluated using strict quality assessment tools. Nine studies explicitly reported random allocation schemes, and two clearly documented allocation concealment methods, indicating improved literature quality compared to previous studies. Meta-analysis of the 17 included studies revealed: 1) Compared with control groups, HLWDD may significantly improve waist circumference (WC), body mass index (BMI), systolic blood pressure (SBP), diastolic blood pressure (DBP), fasting plasma glucose (FPG), 2-h postprandial glucose (2 hPG), triglycerides (TG), high-density lipoprotein cholesterol (HDL-C), and insulin resistance index (HOMA-IR). 2) Compared with the HLWDD group, conventional therapy may effectively improve low-density lipoprotein cholesterol (LDL-C) levels. 3) The HLWDD group may demonstrate superior safety to control groups, though this difference was not statistically significant.

### 4.2 Intrinsic mechanism

HLWDD is prepared by decocting a variety of herbal medicines and offers the advantage of targeting multiple pathways. Modern pharmacological studies suggest that HLWDD may exert its effects by acting on targets such as interleukin-6 (IL-6), protein kinase B1 (AKT1), vascular endothelial growth factor A (VEGFA), matrix metalloproteinase-9 (MMP-9), as well as the P38 mitogen-activated protein kinase (P38MAPK), nuclear factor-κB, phosphoinositide 3-kinase/protein kinase B (PI3K-Akt), and Toll-like receptor 4 (TLR4) to intervene in the development of obstructive sleep apnoea syndrome complicated by MetS (Bao et al., 2021). Another study also indicated that Coptis and Pinellia can effectively target MetS by regulating molecular functions such as G protein-coupled receptor activity and neurotransmitter receptor activity, as well as through pathways including the tumour necrosis factor (TNF) signalling pathway, the oestrogen signalling pathway, and the interleukin-17 (IL-17) IL-17) signalling pathways and endocrine resistance, to effectively influence MetS (Wang et al., 2021). Coptidis rhizome demonstrates significant efficacy in alleviating insulin resistance, reducing blood glucose levels, and improving dyslipidaemia by lowering fasting blood glucose (FBG) and glycated serum protein (GSP) levels, inhibiting HOMA-IR, and decreasing triglycerides (TG), total cholesterol (TC), and low-density lipoprotein cholesterol (LDL-C) levels (Xie et al., 2026).

Synergistic administration of Coptidis rhizome with Glycyrrhizae Radix et Rhizoma enhances glucose uptake in adipocytes, markedly improving lipid profiles and insulin resistance (Zhang et al., 2024). Bambusae Caulis in Taenias effectively ameliorates metabolic dysfunction-associated fatty liver disease by inhibiting lipid biosynthesis and reducing lipid levels (Sun et al., 2025). The primary alkaloid p-synephrine in Aurantii Fructus Immaturus inhibits adipogenesis in 3T3-L1 cells, thereby preventing and treating MetS (Guo et al., 2019). Citri Reticulatae Pericarpium regulates *de novo* fatty acid synthesis, effectively treating obesity, hyperlipidaemia, and hyperglycaemia (Li et al., 2023). Poria cocos not only mitigates hepatic steatosis induced by obesity and high-fat diets but also effectively protects against intestinal and hepatic damage (Ye et al., 2022). Zingiberis Rhizoma Recens exhibits antioxidant and anti-inflammatory properties, preventing and treating cardiovascular and cerebrovascular diseases (Li et al., 2021). Jujubae Fructus possesses anti-obesity effects and protects the liver (Tahergorabi et al., 2015). Animal studies indicate HLWDD effectively treats MetS, consistent with our findings. When Coptidis rhizome is used in combination with Pinelliae rhizome, it can lower fasting blood glucose levels in rats and accelerate gastrointestinal emptying, thereby controlling hyperglycaemia and improving gastrointestinal function (Liang et al., 2021). HLWDD alleviates MetS by inhibiting M1 polarisation in mouse hepatic macrophages, promoting M2 polarisation, and reducing lipid metabolism (Su et al., 2025). Furthermore, HLWDD improves insulin resistance in rats by lowering inflammatory cytokine levels (Li et al., 2021). Another study also suggests that HLWDD may improve MetS by alleviating insulin resistance and inflammatory responses in rats, suppressing the expression of leptin and free fatty acids (FFA), and upregulating GLUT-4 levels (Pang and Li, 2019).

### 4.3 Research limitations

Despite employing rigorous analytical methods, this study retains certain limitations: (1) Among the 17 included studies, eight failed to explicitly describe randomisation methods, and 15 omitted mention of allocation concealment procedures, thereby introducing potential bias risks. This constitutes one of the primary potential sources of heterogeneity in DBP and HDL-C outcomes. (2) As no age restrictions were applied during inclusion, the studies encompassed a broad age range, with the mean minimum age being 38 years and the maximum 60.65 years. Given the physiological differences between patients across these age groups, age emerged as a major potential source of HDL-C heterogeneity. (3) Since all eligible literature originated from China, the study population comprised exclusively Chinese individuals. This presents limitations for generalising treatment protocols to other populations. (4) There is a lack of double-blind RCT studies; due to the principle of individualised treatment in traditional Chinese medicine, there are no standardised guidelines regarding the ingredients or dosages of Huanglian Wendan Decoction, which undermines the scientific validity of the results. (5) Among the 17 included studies, although no adverse reactions were reported for the combination of HLWDD and conventional treatment, the results regarding adverse reactions showed no statistical significance; therefore, the safety of HLWDD remains undetermined.

### 4.4 Research strengths and prospects

Compared with previous reviews (Zhu et al., 2013), this study first incorporates and analyzes the latest clinical trial research, ensuring our conclusions and outlook are grounded in the most cutting-edge knowledge within the field. Secondly, it employs a rigorous and standardised meta-analysis of the included studies, yielding more stringent and accurate research conclusions. Finally, this study systematically collected primary and secondary outcome measures concerning obesity, blood pressure, blood lipids, blood glucose, insulin resistance, and safety indicators, tailored to the characteristics of MetS. A multi-level, multi-dimensional assessment enabled a more comprehensive capture of HLWDD’s true clinical value.

Although the findings suggest HLWDD may represent a potential therapeutic approach for MetS, limitations exist that warrant refinement in future RCT studies. Firstly, RCT protocol design must be enhanced through rigorous adherence to correct randomisation methods, allocation concealment, and blinding protocols to elevate study quality. Secondly, stratified analysis by patient age should be implemented to account for age-related variations in disease progression. Finally, expanding the study cohort is essential to ensure the reliability and reproducibility of findings.

## 5 Conclusion

In summary, HLWDD combined with standard treatment offers greater benefits than standard treatment alone, resulting in significant improvements in WC, BMI, SBP, DBP, FPG, 2 hPG, TG, HDL-C, and HOMA-IR. Furthermore, no adverse reactions have been reported with HLWDD combined with standard treatment, indicating that it is relatively safe. This suggests that HLWDD adjunctive therapy may serve as an alternative treatment for MetS. However, given the limited quality of the included studies, the data remain subject to uncertainty. The results of this meta-analysis should therefore be interpreted with caution and cannot be regarded as definitive clinical evidence. Given the limited quality of the included studies, further rigorous, large-scale, multicentre double-blind randomised controlled trials are required to more fully validate this conclusion.

## Data Availability

The original contributions presented in the study are included in the article/Supplementary Material, further inquiries can be directed to the corresponding authors.
