# Supplementary material for: Efficacy and safety of huanglian wendan decoction as an adjuvant therapy for metabolic syndrome: a systematic review and meta-analysis
Source: Front Pharmacol. 2026 Jun 16;17:1762428. doi: 10.3389/fphar.2026.1762428 (PMC13314610; doi:10.3389/fphar.2026.1762428)

**Supplementary Material**

Supplement to: Efficacy and Safety of Huanglian Wendan Decoction as an Adjuvant Therapy for Metabolic Syndrome: A Systematic Review and Meta-Analysis

Table of Contents

[Supplementary Material 1](#_Toc1219)

[Search strategies 2](#_Toc1088)

[S1. Composition of HLWDD in the treatment group 8](#_Toc7930)

[S2. Sensitivity analysis of WC, BMI, SBP, DBP, FPG, 2hPG, HbA1c, TG, LDL-C, and HDL-C 10](#_Toc7209)

[S3. Subgroup analysis of SBP 13](#_Toc12730)

[S4. Subgroup analysis of DBP 16](#_Toc10781)

[S5. Subgroup analysis of FPG 18](#_Toc13405)

[S6. Subgroup analysis of 2hPG 20](#_Toc8021)

[S7. Subgroup analysis of LDL-C 22](#_Toc21877)

[S8. Subgroup analysis of HDL-C 24](#_Toc20839)

[S9. Meta-regression analysis of SBP 26](#_Toc14414)

[S20. Meta-regression analysis of DBP 27](#_Toc4694)

[S21. Meta-regression analysis of FPG 29](#_Toc15153)

[S22. Meta-regression analysis of 2hPG 31](#_Toc12856)

[S23. Meta-regression analysis of LDL-C 33](#_Toc29965)

[S24. Meta-regression analysis of HDL-C 35](#_Toc32450)

[S25. Publication bias funnel plot of BMI, SBP, DBP, FPG, 2hPG, TG, LDL-C, and HDL-C 37](#_Toc19769)

[S26. Egger’s test of BMI, SBP, DBP, FPG, 2hPG, TG, LDL-C, and HDL-C 41](#_Toc14460)

[S27. Trimming and filling method of SBP and TG 44](#_Toc26216)

**Search strategies**

***PubMed:***

("Metabolic Syndrome"[MeSH Terms] OR ("Metabolic Syndrome"[MeSH Terms] OR ("metabolic"[All Fields] AND "syndrome"[All Fields]) OR "Metabolic Syndrome"[All Fields] OR ("metabolic"[All Fields] AND "syndromes"[All Fields]) OR "metabolic syndromes"[All Fields] OR ("Metabolic Syndrome"[MeSH Terms] OR ("metabolic"[All Fields] AND "syndrome"[All Fields]) OR "Metabolic Syndrome"[All Fields] OR ("syndrome"[All Fields] AND "metabolic"[All Fields]) OR "syndrome metabolic"[All Fields]) OR ("Metabolic Syndrome"[MeSH Terms] OR ("metabolic"[All Fields] AND "syndrome"[All Fields]) OR "Metabolic Syndrome"[All Fields] OR ("syndromes"[All Fields] AND "metabolic"[All Fields]) OR "syndromes metabolic"[All Fields]) OR ("Metabolic Syndrome"[MeSH Terms] OR ("metabolic"[All Fields] AND "syndrome"[All Fields]) OR "Metabolic Syndrome"[All Fields] OR "reaven syndrome x"[All Fields]) OR ("Metabolic Syndrome"[MeSH Terms] OR ("metabolic"[All Fields] AND "syndrome"[All Fields]) OR "Metabolic Syndrome"[All Fields] OR "syndrome x reaven"[All Fields]) OR ("Metabolic Syndrome"[MeSH Terms] OR ("metabolic"[All Fields] AND "syndrome"[All Fields]) OR "Metabolic Syndrome"[All Fields] OR "metabolic syndrome x"[All Fields]) OR ("Metabolic Syndrome"[MeSH Terms] OR ("metabolic"[All Fields] AND "syndrome"[All Fields]) OR "Metabolic Syndrome"[All Fields] OR "insulin resistance syndrome x"[All Fields]) OR ("Metabolic Syndrome"[MeSH Terms] OR ("metabolic"[All Fields] AND "syndrome"[All Fields]) OR "Metabolic Syndrome"[All Fields] OR ("metabolic"[All Fields] AND "cardiovascular"[All Fields] AND "syndrome"[All Fields]) OR "metabolic cardiovascular syndrome"[All Fields]) OR ("Metabolic Syndrome"[MeSH Terms] OR ("metabolic"[All Fields] AND "syndrome"[All Fields]) OR "Metabolic Syndrome"[All Fields] OR ("cardiovascular"[All Fields] AND "syndrome"[All Fields] AND "metabolic"[All Fields]) OR "cardiovascular syndrome metabolic"[All Fields]) OR ("Metabolic Syndrome"[MeSH Terms] OR ("metabolic"[All Fields] AND "syndrome"[All Fields]) OR "Metabolic Syndrome"[All Fields] OR ("cardiovascular"[All Fields] AND "syndromes"[All Fields] AND "metabolic"[All Fields])) OR ("Metabolic Syndrome"[MeSH Terms] OR ("metabolic"[All Fields] AND "syndrome"[All Fields]) OR "Metabolic Syndrome"[All Fields] OR ("syndrome"[All Fields] AND "metabolic"[All Fields] AND "cardiovascular"[All Fields]) OR "syndrome metabolic cardiovascular"[All Fields]) OR ("Metabolic Syndrome"[MeSH Terms] OR ("metabolic"[All Fields] AND "syndrome"[All Fields]) OR "Metabolic Syndrome"[All Fields] OR "syndrome x insulin resistance"[All Fields]) OR ("Metabolic Syndrome"[MeSH Terms] OR ("metabolic"[All Fields] AND "syndrome"[All Fields]) OR "Metabolic Syndrome"[All Fields] OR "metabolic x syndrome"[All Fields]) OR ("Metabolic Syndrome"[MeSH Terms] OR ("metabolic"[All Fields] AND "syndrome"[All Fields]) OR "Metabolic Syndrome"[All Fields] OR "syndrome metabolic x"[All Fields]) OR ("Metabolic Syndrome"[MeSH Terms] OR ("metabolic"[All Fields] AND "syndrome"[All Fields]) OR "Metabolic Syndrome"[All Fields] OR "x syndrome metabolic"[All Fields]) OR ("Metabolic Syndrome"[MeSH Terms] OR ("metabolic"[All Fields] AND "syndrome"[All Fields]) OR "Metabolic Syndrome"[All Fields] OR "syndrome x metabolic"[All Fields]) OR ("Metabolic Syndrome"[MeSH Terms] OR ("metabolic"[All Fields] AND "syndrome"[All Fields]) OR "Metabolic Syndrome"[All Fields] OR "dysmetabolic syndrome x"[All Fields]) OR ("Metabolic Syndrome"[MeSH Terms] OR ("metabolic"[All Fields] AND "syndrome"[All Fields]) OR "Metabolic Syndrome"[All Fields]) OR ("Metabolic Syndrome"[MeSH Terms] OR ("metabolic"[All Fields] AND "syndrome"[All Fields]) OR "Metabolic Syndrome"[All Fields] OR ("cardiometabolic"[All Fields] AND "syndrome"[All Fields]) OR "cardiometabolic syndrome"[All Fields]) OR ("Metabolic Syndrome"[MeSH Terms] OR ("metabolic"[All Fields] AND "syndrome"[All Fields]) OR "Metabolic Syndrome"[All Fields] OR ("cardiometabolic"[All Fields] AND "syndromes"[All Fields]) OR "cardiometabolic syndromes"[All Fields]) OR ("Metabolic Syndrome"[MeSH Terms] OR ("metabolic"[All Fields] AND "syndrome"[All Fields]) OR "Metabolic Syndrome"[All Fields] OR ("syndrome"[All Fields] AND "cardiometabolic"[All Fields]) OR "syndrome cardiometabolic"[All Fields]) OR ("Metabolic Syndrome"[MeSH Terms] OR ("metabolic"[All Fields] AND "syndrome"[All Fields]) OR "Metabolic Syndrome"[All Fields] OR ("syndromes"[All Fields] AND "cardiometabolic"[All Fields])))) AND ("wendan"[Supplementary Concept] OR "wendan"[All Fields] OR "wen dan decoction"[All Fields] OR "wendan"[Supplementary Concept]) AND ("randomized controlled trial"[Publication Type] OR "randomized controlled trials as topic"[MeSH Terms] OR "randomized controlled trial"[All Fields] OR "randomised controlled trial"[All Fields] OR ("controlled clinical trial"[Publication Type] OR "controlled clinical trials as topic"[MeSH Terms] OR "controlled clinical trial"[All Fields] OR "Randomly"[All Fields] OR ("random allocation"[MeSH Terms] OR ("random"[All Fields] AND "allocation"[All Fields]) OR "random allocation"[All Fields] OR "randomization"[All Fields] OR "randomized"[All Fields] OR "random"[All Fields] OR "randomisation"[All Fields] OR "randomisations"[All Fields] OR "randomise"[All Fields] OR "randomised"[All Fields] OR "randomising"[All Fields] OR "randomizations"[All Fields] OR "randomize"[All Fields] OR "randomizes"[All Fields] OR "randomizing"[All Fields] OR "randomness"[All Fields] OR "randoms"[All Fields]) OR ("random allocation"[MeSH Terms] OR ("random"[All Fields] AND "allocation"[All Fields]) OR "random allocation"[All Fields] OR "randomization"[All Fields] OR "randomized"[All Fields] OR "random"[All Fields] OR "randomisation"[All Fields] OR "randomisations"[All Fields] OR "randomise"[All Fields] OR "randomised"[All Fields] OR "randomising"[All Fields] OR "randomizations"[All Fields] OR "randomize"[All Fields] OR "randomizes"[All Fields] OR "randomizing"[All Fields] OR "randomness"[All Fields] OR "randoms"[All Fields]) OR ("random allocation"[MeSH Terms] OR ("random"[All Fields] AND "allocation"[All Fields]) OR "random allocation"[All Fields]) OR ("clinical trials as topic"[MeSH Terms] OR ("clinical"[All Fields] AND "trials"[All Fields] AND "topic"[All Fields]) OR "clinical trials as topic"[All Fields] OR "trial"[All Fields] OR "trial s"[All Fields] OR "trialed"[All Fields] OR "trialing"[All Fields] OR "trials"[All Fields])))

***Web of Science:***

#1 Metabolic Syndrome (Topic) or Metabolic Syndromes (Topic) or Syndrome, Metabolic (Topic) or Syndromes, Metabolic (Topic) or Reaven Syndrome X (Topic) or Syndrome X, Reaven (Topic) or Metabolic Syndrome X (Topic) or Insulin Resistance Syndrome X (Topic) or Metabolic Cardiovascular Syndrome (Topic) or Cardiovascular Syndrome, Metabolic (Topic) or Cardiovascular Syndromes, Metabolic (Topic) or Syndrome, Metabolic Cardiovascular (Topic) or Syndrome X, Insulin Resistance (Topic) or Metabolic X Syndrome (Topic) or Syndrome, Metabolic X (Topic) or X Syndrome, Metabolic (Topic) or Syndrome X, Metabolic (Topic) or Dysmetabolic Syndrome X (Topic) or Syndrome X, Dysmetabolic (Topic) or Cardiometabolic Syndrome (Topic) or Cardiometabolic Syndromes (Topic) or Syndrome, Cardiometabolic (Topic) or Syndromes, Cardiometabolic (Topic)

#2 wendan (Topic) or wen-dan decoction (Topic)

#3 Randomized controlled trial (Topic) or Controlled clinical trial (Topic) or Randomly (Topic) or Randomized (Topic) or Randomised (Topic) or Random allocation (Topic) or Trials (Topic)

#4 #1 AND #2 AND #3

***Embase:***

#1 "Metabolic Syndrome":ab,ti or "Metabolic Syndromes":ab,ti or "Syndrome, Metabolic":ab,ti or "Syndromes, Metabolic":ab,ti or "Reaven Syndrome X":ab,ti or "Syndrome X, Reaven":ab,ti or "Metabolic Syndrome X":ab,ti or "Insulin Resistance Syndrome X":ab,ti or "Metabolic Cardiovascular Syndrome":ab,ti or "Cardiovascular Syndrome, Metabolic":ab,ti or "Cardiovascular Syndromes, Metabolic":ab,ti or "Syndrome, Metabolic Cardiovascular":ab,ti or "Syndrome X, Insulin Resistance":ab,ti or "Metabolic X Syndrome":ab,ti or "Syndrome, Metabolic X":ab,ti or "X Syndrome, Metabolic":ab,ti or "Syndrome X, Metabolic":ab,ti or "Dysmetabolic Syndrome X":ab,ti or "Syndrome X, Dysmetabolic":ab,ti or "Cardiometabolic Syndrome":ab,ti or "Cardiometabolic Syndromes":ab,ti or "Syndrome, Cardiometabolic":ab,ti or "Syndromes, Cardiometabolic"

#2 "wendan":ab,ti or "wen-dan decoction"

#3 "Randomized controlled trial":ab,ti or "Controlled clinical trial":ab,ti or "Randomly":ab,ti or "Randomized":ab,ti or "Randomised":ab,ti or "Random allocation":ab,ti or "Trials"

#4 #1 AND #2 AND #3

***Cochrane Central Register of Controlled Trials：***

#1 (Metabolic Syndrome):ti,ab,kw OR (Metabolic Syndromes):ti,ab,kw OR (Syndrome, Metabolic):ti,ab,kw OR (Syndromes, Metabolic):ti,ab,kw OR (Reaven Syndrome X):ti,ab,kw OR (Syndrome X, Reaven):ti,ab,kw OR (Metabolic Syndrome X):ti,ab,kw OR (Insulin Resistance Syndrome X):ti,ab,kw OR (Metabolic Cardiovascular Syndrome):ti,ab,kw OR (Cardiovascular Syndrome, Metabolic):ti,ab,kw OR (Cardiovascular Syndromes, Metabolic):ti,ab,kw OR (Syndrome, Metabolic Cardiovascular):ti,ab,kw OR (Syndrome X, Insulin Resistance):ti,ab,kw OR (Metabolic X Syndrome):ti,ab,kw OR (Syndrome, Metabolic X):ti,ab,kw OR (X Syndrome, Metabolic):ti,ab,kw OR (Syndrome X, Metabolic):ti,ab,kw OR (Dysmetabolic Syndrome X):ti,ab,kw OR (Syndrome X, Dysmetabolic):ti,ab,kw OR (Cardiometabolic Syndrome):ti,ab,kw OR (Cardiometabolic Syndromes):ti,ab,kw OR (Syndrome, Cardiometabolic):ti,ab,kw OR (Syndromes, Cardiometabolic):ti,ab,kw

#2 (wendan):ti,ab,kw OR (wen-dan decoction):ti,ab,kw

#3 (Randomized controlled trial):ti,ab,kw OR (Controlled clinical trial):ti,ab,kw OR (Randomly):ti,ab,kw OR (Randomized):ti,ab,kw OR (Randomised):ti,ab,kw OR (Random allocation):ti,ab,kw OR (Trials):ti,ab,kw

#4 #1 AND #2 AND #3

***Chinese National Knowledge Infrastructure (CNKI)：***

( SU=温胆 OR SU=温胆汤 OR SU=黄连温胆汤 OR SU=加味温胆汤) AND (SU=代谢综合征 OR SU=代谢综合征(ms) OR SU=代谢综合征(mets) OR SU=代谢综合症 OR SU=胰岛素抵抗综合症 OR SU=胰岛素抵抗综合征) AND (SU=随机对照实验 OR SU=随机 OR SU=对照 OR SU=临床 OR SU=疗效)

***Chinese BioMedical Literature Database：***

("温胆"[常用字段:智能] OR "温胆汤"[常用字段:智能] OR "黄连温胆汤"[常用字段:智能] OR "加味温胆汤"[常用字段:智能]) AND ("代谢综合征"[常用字段:智能] OR "代谢综合症"[常用字段:智能] OR "胰岛素抵抗综合症"[常用字段:智能] OR "胰岛素抵抗综合征"[常用字段:智能] ) AND ("随机对照试验"[常用字段:智能] OR "随机对照实验"[常用字段:智能] OR "随机对照研究"[摘要:智能])

***Chinese Scientific Journal Database (VIP):***

U=温胆汤 AND U=(代谢综合征+metabolic syndrom e+metabolic syndrome+metabolism syndrome+代谢综合症+胰岛素抵抗综合症+胰岛素抵抗综合征) AND U=(随机对照试验+randomized clinical trials+randomized controlled clinical trial+randomized controlled trial+randomized controlled trials+randomized experiment+rct+随机对照实验+随机对照研究)

***WanFang database：***

主题:(温胆 or 温胆汤 or 黄连温胆汤 or 加味温胆汤) and 主题:(代谢综合征 or 代谢综合征(ms) or 代谢综合征(mets) or 代谢综合症 or 胰岛素抵抗综合症 or 胰岛素抵抗综合征) and 主题:(随机对照实验 or 随机 or 对照 or 临床 or 疗效)

**S1. Composition of HLWDD in the treatment group**

| **Inclusion studies** | **Medicine** |
| --- | --- |
| Bai 2015 | Coptidis rhizome 15g, Pinelliae Rhizoma Praeparatum 12g, Citri Reticulatae Pericarpium 15g, Bambusae Caulis in Taenias 18g, Poria 30g, Aurantii Fructus Immaturus 18g, Glycyrrhizae Radix et Rhizoma 9g, Zingiberis Rhizoma Recens 5 slices, Jujubae Fructus 2 pieces. |
| Ding 2025 | Poria 20g, Citri Reticulatae Pericarpium 15g, Pinelliae Rhizoma 15g, Puerariae lobatae radix 15g, Herba eupatorii 10g, Bambusae Caulis in Taenias 10g, Coptidis rhizome 10g, Glycyrrhizae Radix et Rhizoma 6g. |
| Dong 2025 | Coptidis rhizome 15g, Pinelliae Rhizoma Praeparatum Cum Alumine 10g, Aurantii Fructus Immaturus 15g, Bambusae Caulis in Taenias 10g, Citri Reticulatae Pericarpium 15g, Poria 15g, Glycyrrhizae Radix et Rhizoma 10g, Eucommiae cortex 20g, Achyranthis bidentatae radix 20g, Puerariae lobatae radix 20g, Salviae Miltiorrhizae Radix et Rhizoma 10g, Herba eupatorii 15g.. |
| Han 2025 | Coptidis rhizome 10g, Pinelliae Rhizoma Praeparatum Cum Alumine 10g, Aurantii Fructus Immaturus 10g, Bambusae Caulis in Taenias 10g, Citri Reticulatae Pericarpium 20g, Poria 20g, Glycyrrhizae Radix et Rhizoma 10g, Gastrodiae Rhizoma 15g, Eucommiae cortex 20g, Achyranthis bidentatae radix 20g, Puerariae lobatae radix 20g, Salviae Miltiorrhizae Radix et Rhizoma 10g, Herba eupatorii 20g. |
| Jiang 2018 | Coptidis rhizome 10g, Poria 20g, Pinelliae Rhizoma 20g, Citri Reticulatae Pericarpium 20g, Bambusae Caulis in Taenias 10g, Herba eupatorii 10g, Puerariae lobatae radix 20g, Glycyrrhizae Radix et Rhizoma 10g. |
| Jin 2021 | Pseudostellariae Radix 25g, Coptidis rhizome 6g, Pinelliae Rhizoma 9g, Flos Mume 20g, Gastrodiae Rhizoma 15g, Puerariae lobatae radix 25g, Bambusae Caulis in Taenias 10g, Citri Reticulatae Pericarpium 12g, Atractylodis macrocephalae rhizoma15g, Persicae Semen 10g, Carthami Flos 15g. |
| Liu 2016 | Coptidis rhizome 10g, Pinelliae Rhizoma Praeparatum 20g, Citri Reticulatae Pericarpium 20g, Bambusae Caulis in Taenias 20g, Poria 20g, Puerariae lobatae radix 20g, Cassiae semen 30 g, Astragali Radix 30 g, Salviae Miltiorrhizae Radix et Rhizoma 20g, Glycyrrhizae Radix et Rhizoma 10g. |
| Ma 2020 | Coptidis rhizome 10g, Glycyrrhizae Radix et Rhizoma 10g, Pinelliae Rhizoma Praeparatum 20g, Citri Reticulatae Pericarpium 20g, Poria 20g, Puerariae lobatae radix 20g, Salviae Miltiorrhizae Radix et Rhizoma 20g, Bambusae Caulis in Taenias 20g, Cassiae semen 30g, Astragali Radix 30g. |
| Meng 2012 | Coptidis rhizome, Pinelliae Rhizoma, Citri Reticulatae Pericarpium, Bambusae Caulis in Taenias, Poria, Puerariae lobatae radix, Cassiae semen, Astragali Radix, Salviae Miltiorrhizae Radix et Rhizoma, Glycyrrhizae Radix et Rhizoma. |
| Sui 2015 | Coptidis rhizome 10g, Pinelliae Rhizoma 15g, Bambusae Caulis in Taenias 15g, Aurantii Fructus Immaturus 15g, Citri Reticulatae Pericarpium 15g, Poria 20g, Glycyrrhizae Radix et Rhizoma 10g. |
| Wang 2012 | Citri Reticulatae Pericarpium 10g, Pinelliae Rhizoma 12g, Poria 20g, Aurantii Fructus Immaturus 6g, Coptidis rhizome 9g, Bambusae Caulis in Taenias 9g, Glycyrrhizae Radix et Rhizoma 6g, Jujubae Fructus 6 pieces.For those with obvious deficiency of qi, along with: Astragali Radix 30g, Codonopsis radix 20g. The symptoms of blood stasis are obvious, along with: Salviae Miltiorrhizae Radix et Rhizoma 20g, Chuanxiong Rhizoma 20g. |
| Wang 2022 | Citri Reticulatae Pericarpium 20g, Coptidis rhizome 10g, Pinelliae Rhizoma 10g, Poria 20g, Bambusae Caulis in Taenias 10g, Aurantii Fructus Immaturus 10g, Glycyrrhizae Radix et Rhizoma 10g, Puerariae lobatae radix 20g, Angelicae Sinensis Radix 20g, Herba eupatorii 20g, Salviae Miltiorrhizae Radix et Rhizoma 10g. |
| Wang 2023 | Coptidis rhizome 10g, Pinelliae Rhizoma Praeparatum Cum Alumine 10g, Aurantii Fructus Immaturus 10g, Bambusae Caulis in Taenias 10g, Citri Reticulatae Pericarpium 15g, Poria 15g, Glycyrrhizae Radix et Rhizoma 10g, Gastrodiae Rhizoma 15g, Eucommiae cortex 20g, Achyranthis bidentatae radix 20g, Puerariae lobatae radix 15g, Salviae Miltiorrhizae Radix et Rhizoma 10g, Herba eupatorii 15g. |
| Xian 2021 | Coptidis rhizome 10g, Citri Reticulatae Pericarpium 10g, Bambusae Caulis in Taenias 20g, Poria 10g, Puerariae lobatae radix 10g, Astragali Radix 20g, Salviae Miltiorrhizae Radix et Rhizoma 20g, Glycyrrhizae Radix et Rhizoma 10g. |
| Xu 2008 | Coptidis rhizome 12g, Scutellariae Radix 9g, Pinelliae Rhizoma 9g, Citri Reticulatae Pericarpium 12g, Poria 12g, Glycyrrhizae Radix et Rhizoma 6g, Bambusae Caulis in Taenias 12g, Crataegi fructus 12g, Raphani Semen 12g, Chuanxiong Rhizoma 12g, Gastrodiae Rhizoma 12g, Trichosanthis Radix 15g. Feeling dizzy, nauseous, and also: Atractylodis macrocephalae rhizoma 12g. Shortness of breath and chest tightness, along with: Aconiti Lateralis Radix Praeparaia 6g, Codonopsis radix 15g. Abdominal distension and chest tightness, along with: Cyperi Rhizoma 12g. |
| Xu 2009 | Pinelliae Rhizoma, Citri Reticulatae Pericarpium, Poria, Glycyrrhizae Radix et Rhizoma, Aurantii Fructus Immaturus, Bambusae Caulis in Taenias, Coptidis rhizome, Jujubae Fructus. |
| Yang 2025 | Coptidis rhizome 10g, Pinelliae Rhizoma Praeparatum Cum Alumine 10g, Citri Reticulatae Pericarpium 20g, Bambusae Caulis in Taenias 20g, Poria 20g, Glycyrrhizae Radix et Rhizoma 10g, Atractylodis macrocephalae rhizoma 20g, Puerariae lobatae radix 20g, Salviae Miltiorrhizae Radix et Rhizoma 10g, Rhodiolae Crenulatae Radix et Rhizoma 20g. |

**S2. Sensitivity analysis of WC, BMI, SBP, DBP, FPG, 2hPG, HbA1c, TG, LDL-C, and HDL-C**

S2.1 WC


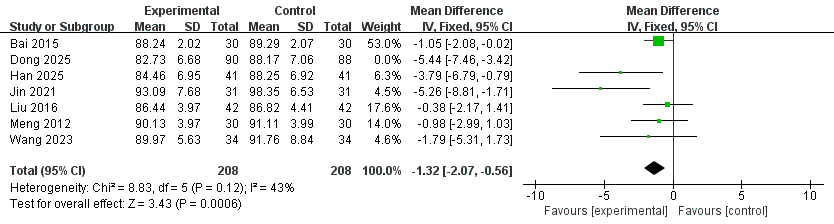


S2.2 BMI


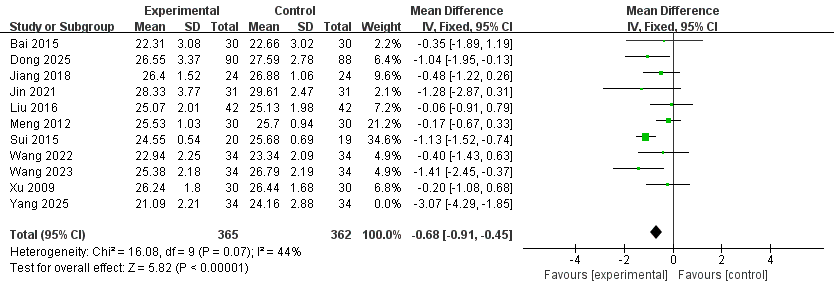


S2.3 SBP


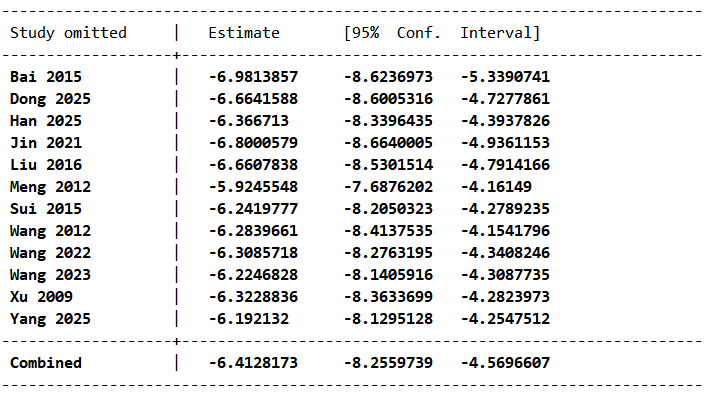


S2.4 DBP


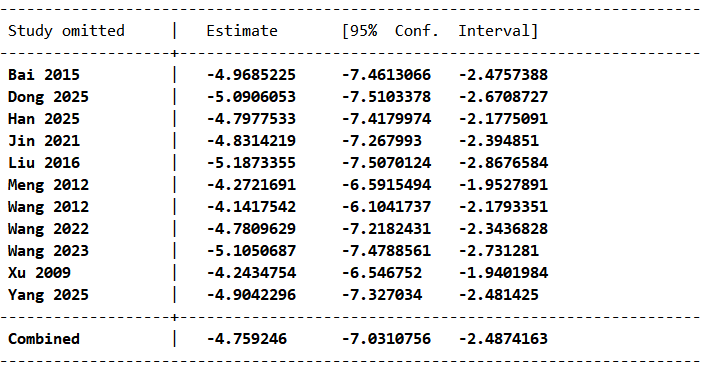


S2.5 FPG


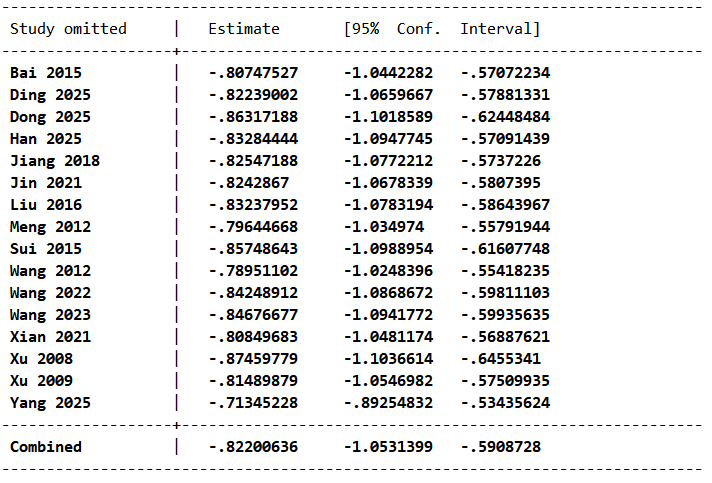


S2.6 2hPG


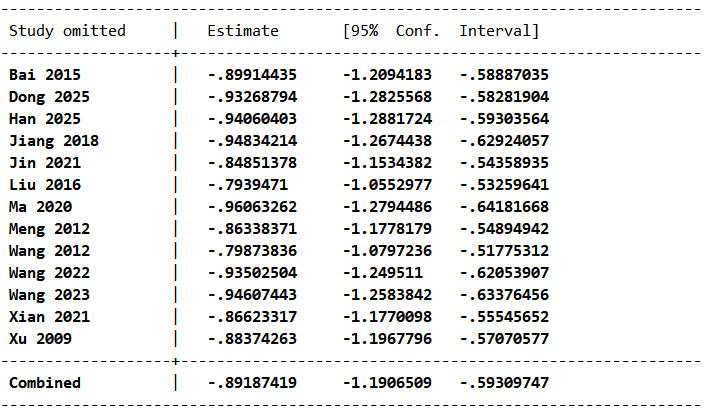


S2.7 HbA1c


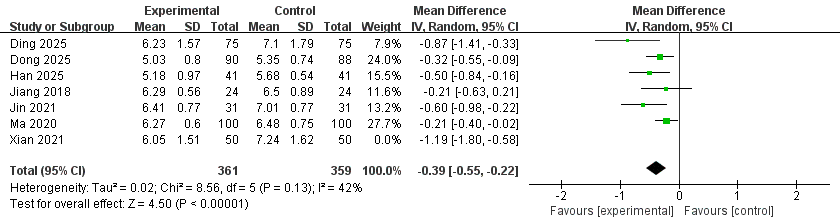


S2.8 TG


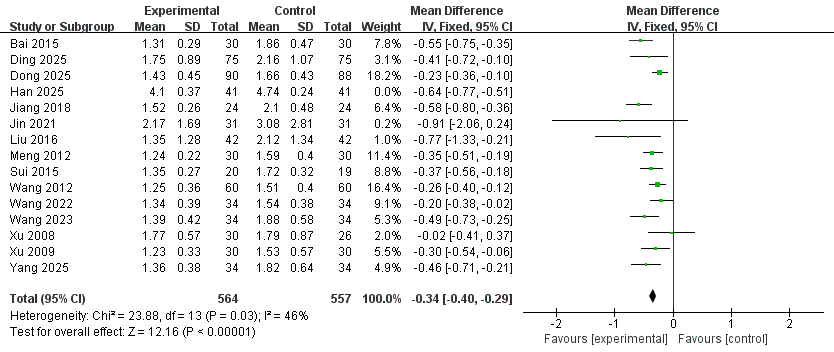


S2.9 LDL-C


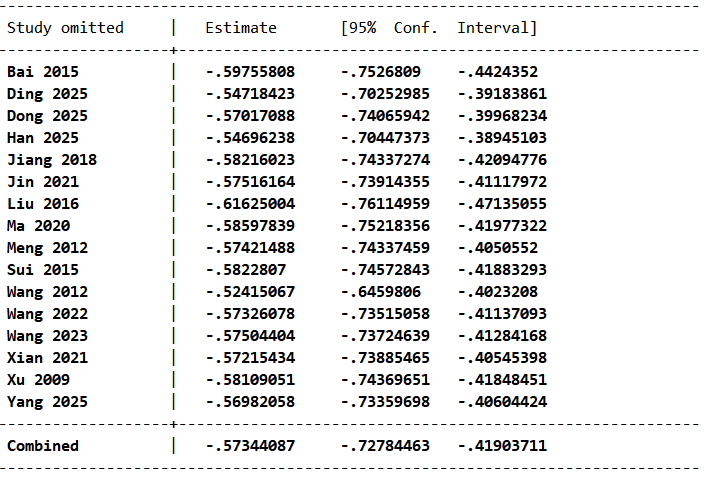


S2.10 HDL-C


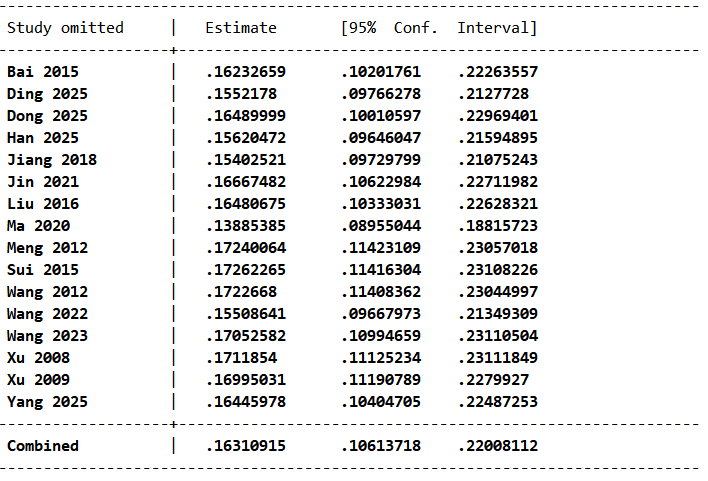


**S3. Subgroup analysis of SBP**

S3.1 Random sequence generation


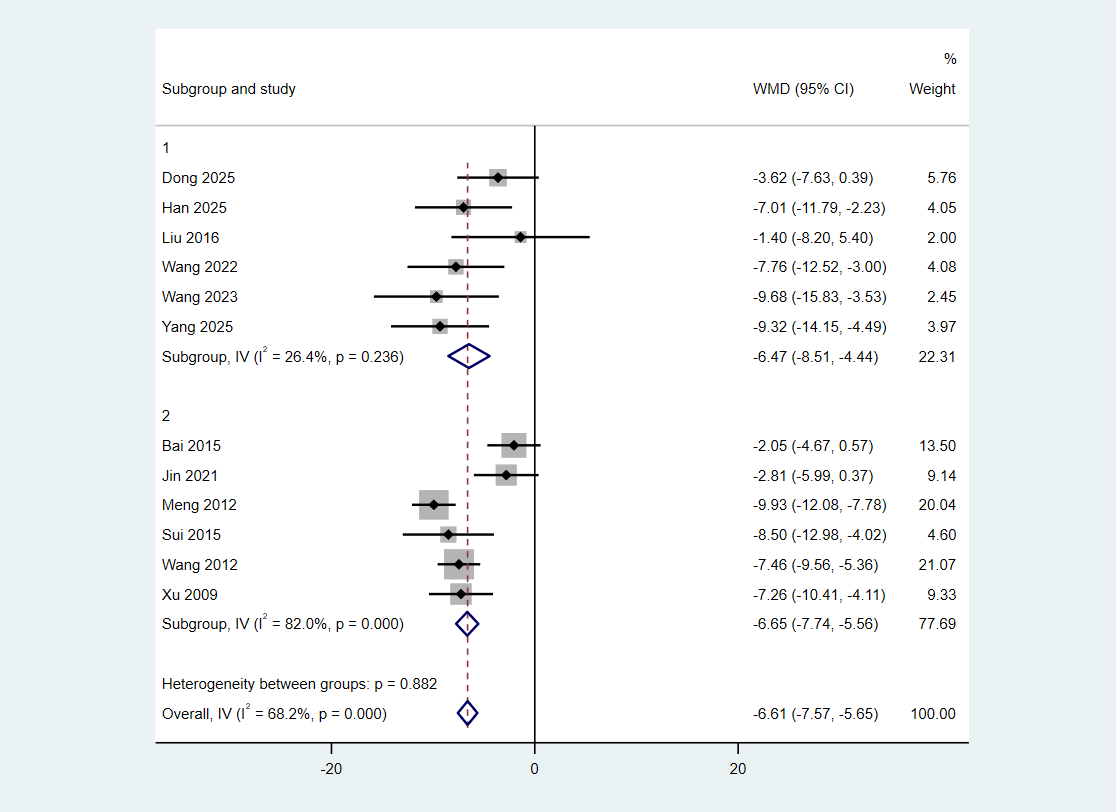


S3.2 Number


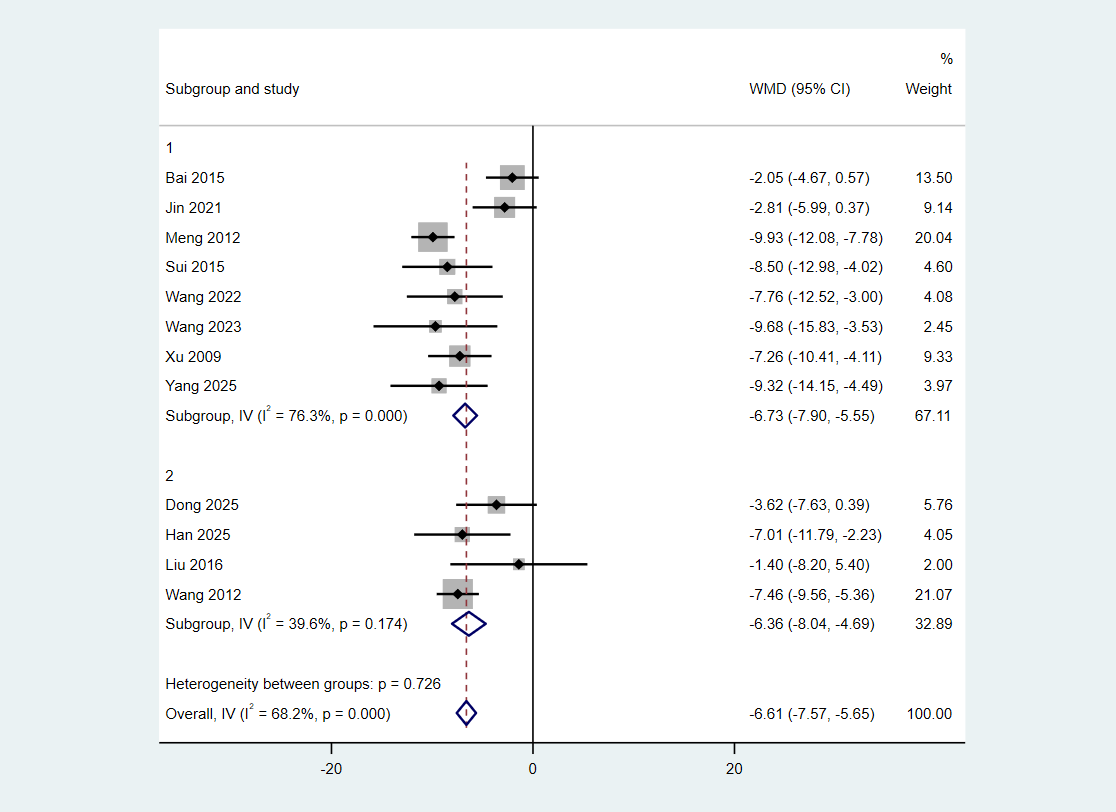


S3.3 Age


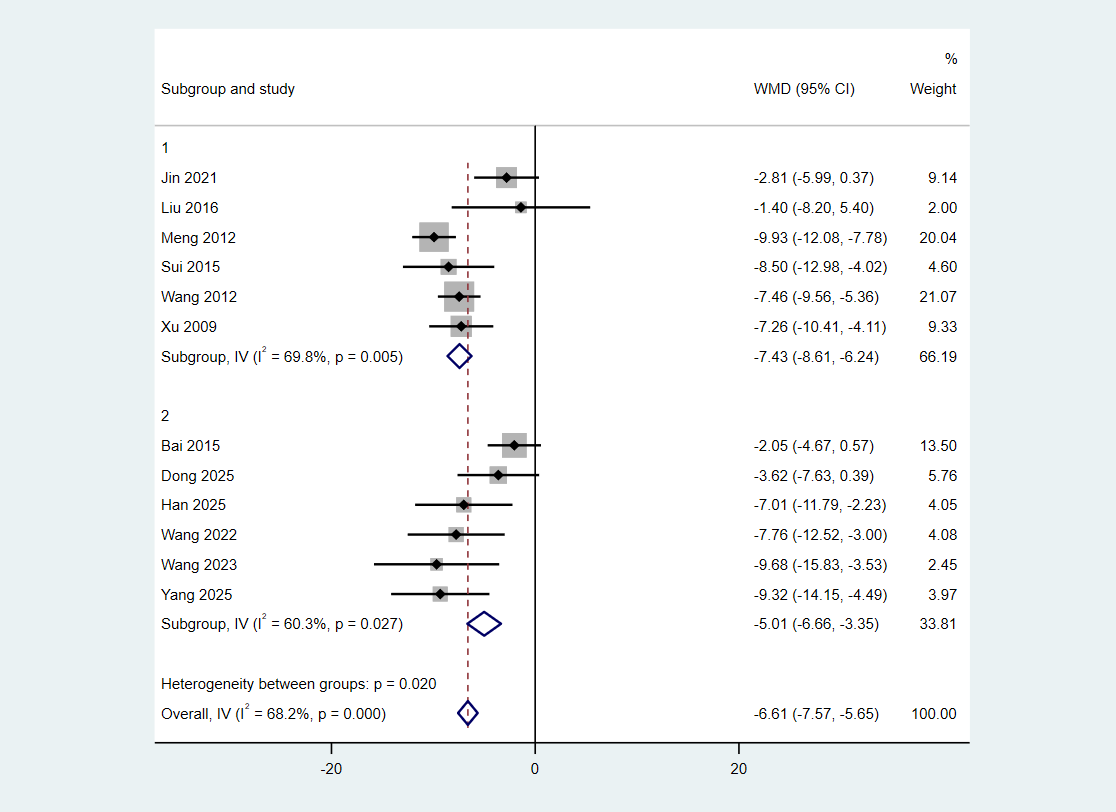


S3.4 Duration


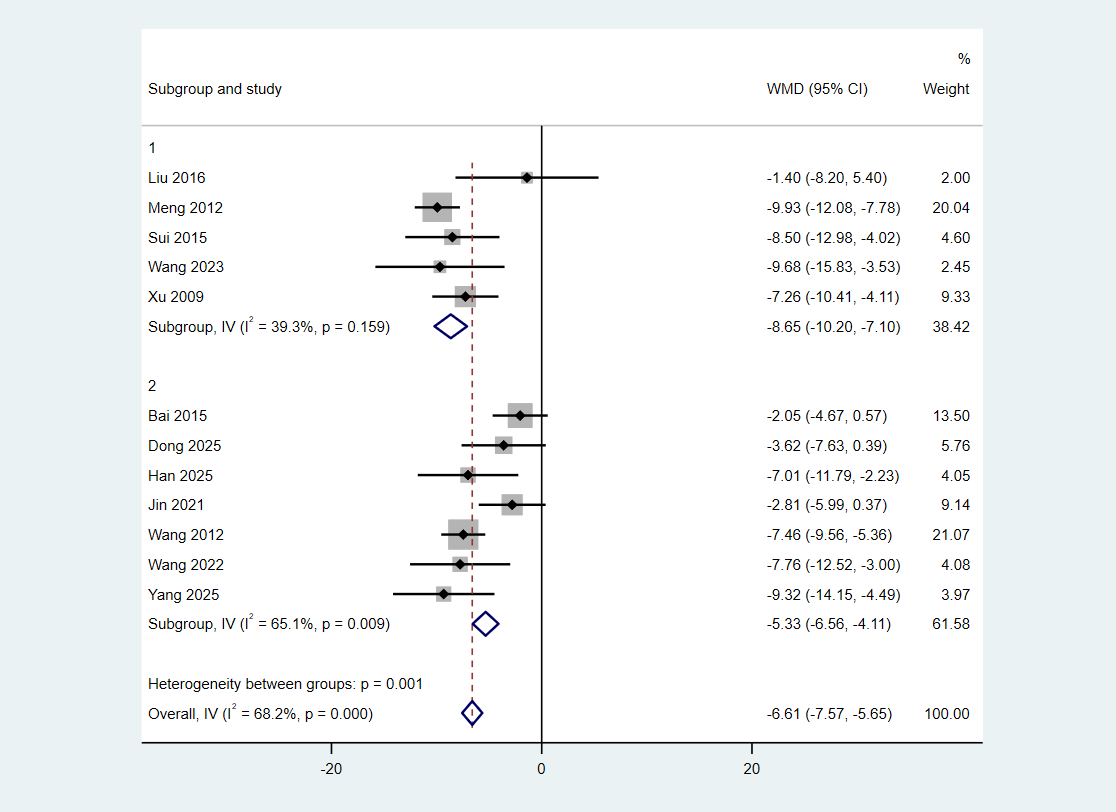


**S4. Subgroup analysis of DBP**

S4.1 Random sequence generation


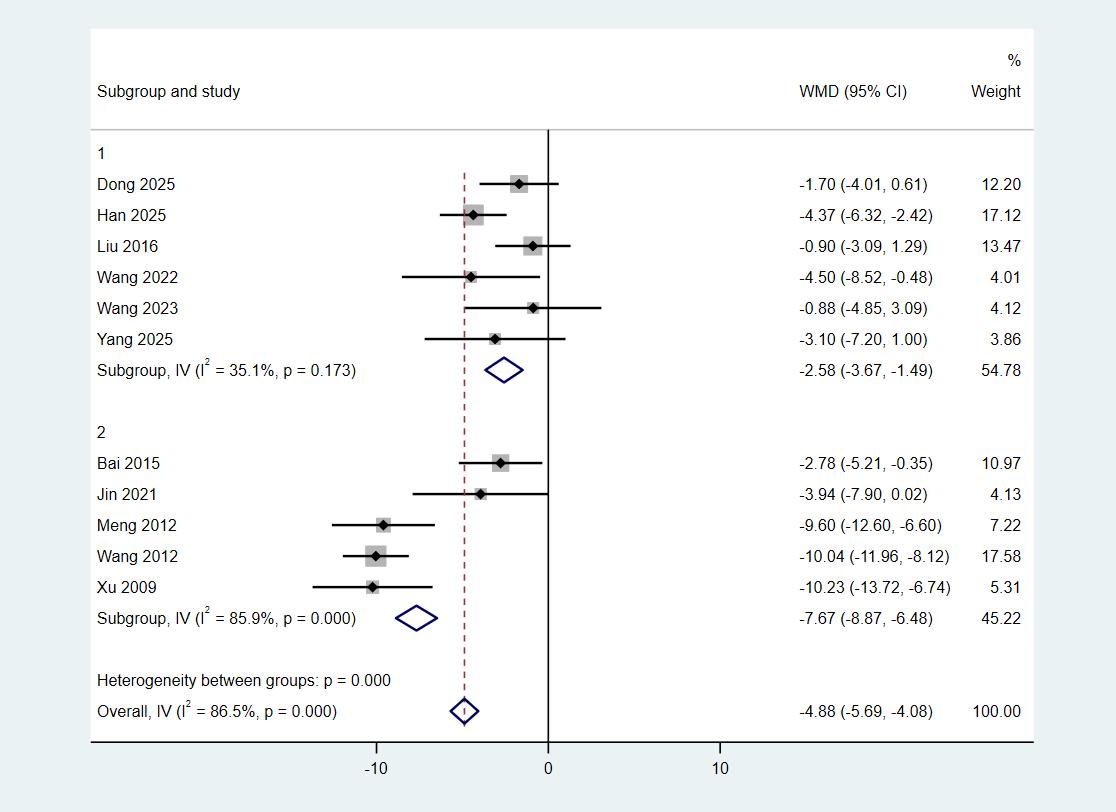


S4.2 Number


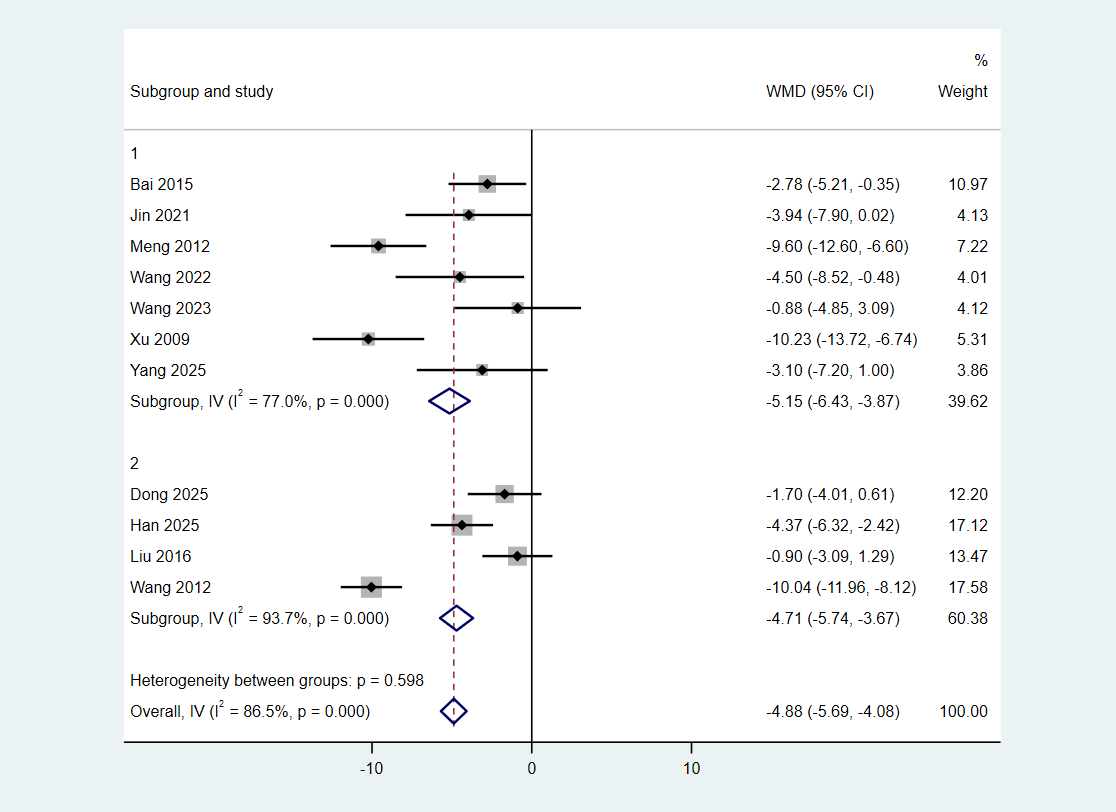


S4.3 Age


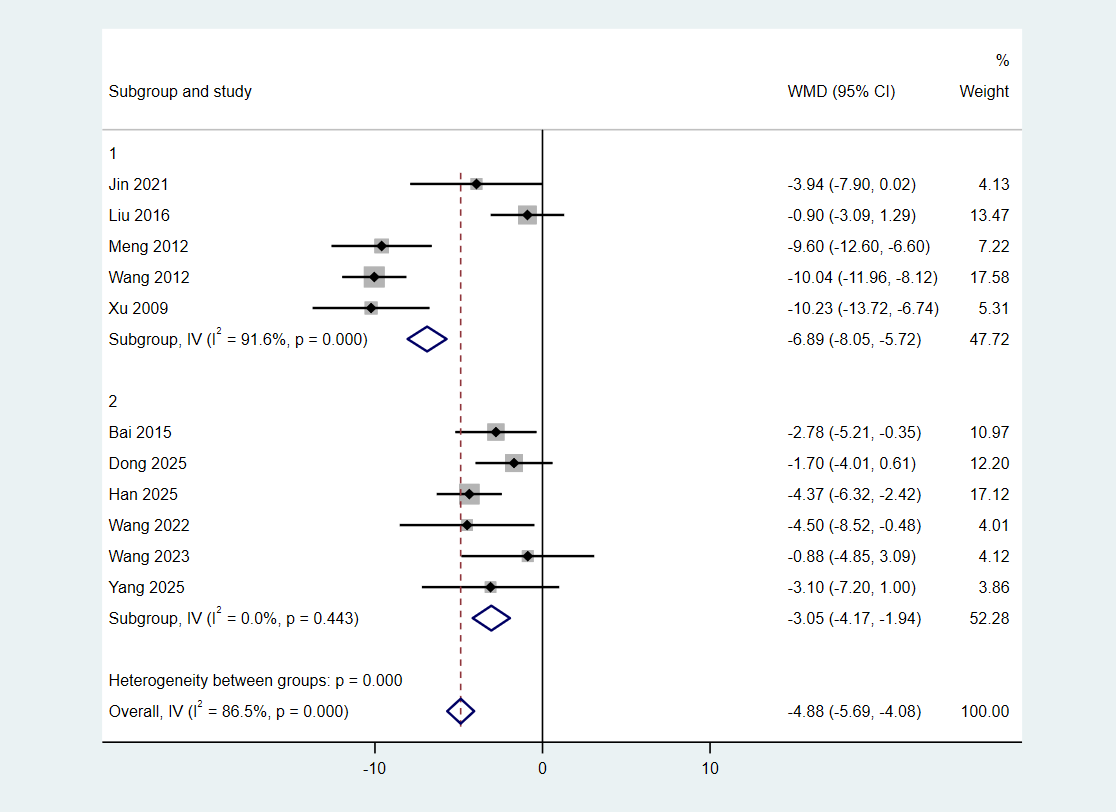


S4.4 Duration


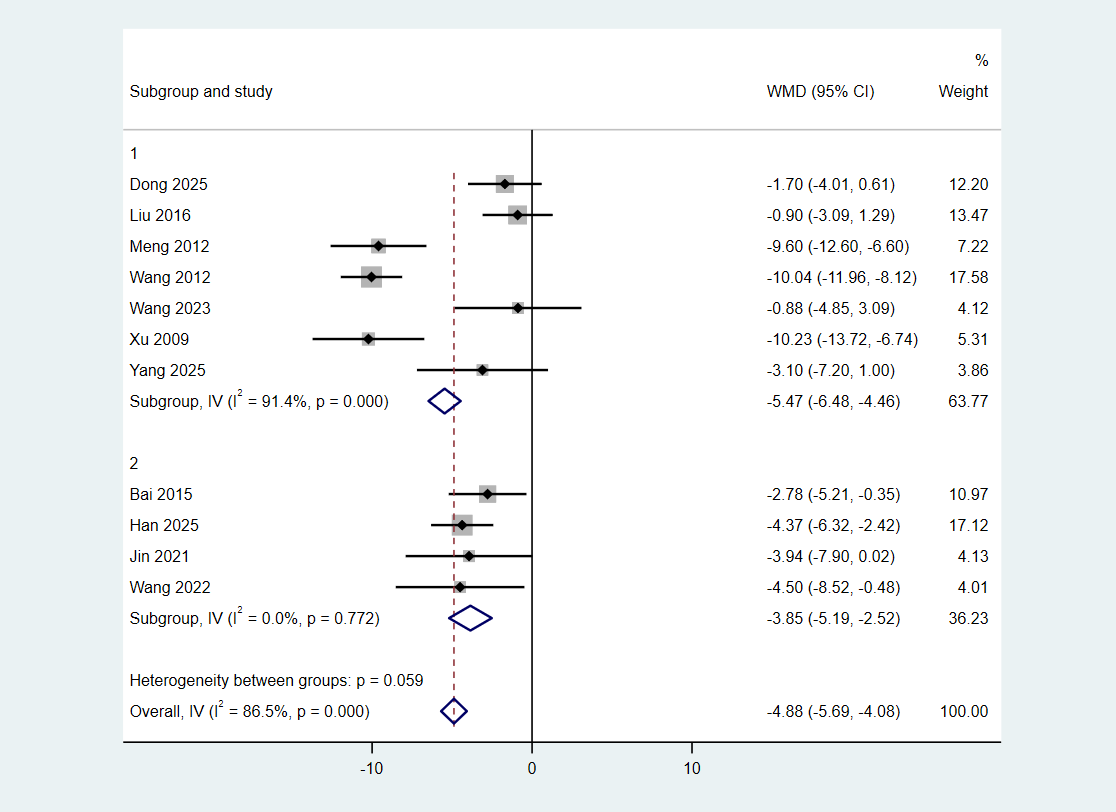


**S5. Subgroup analysis of FPG**

S5.1 Random sequence generation


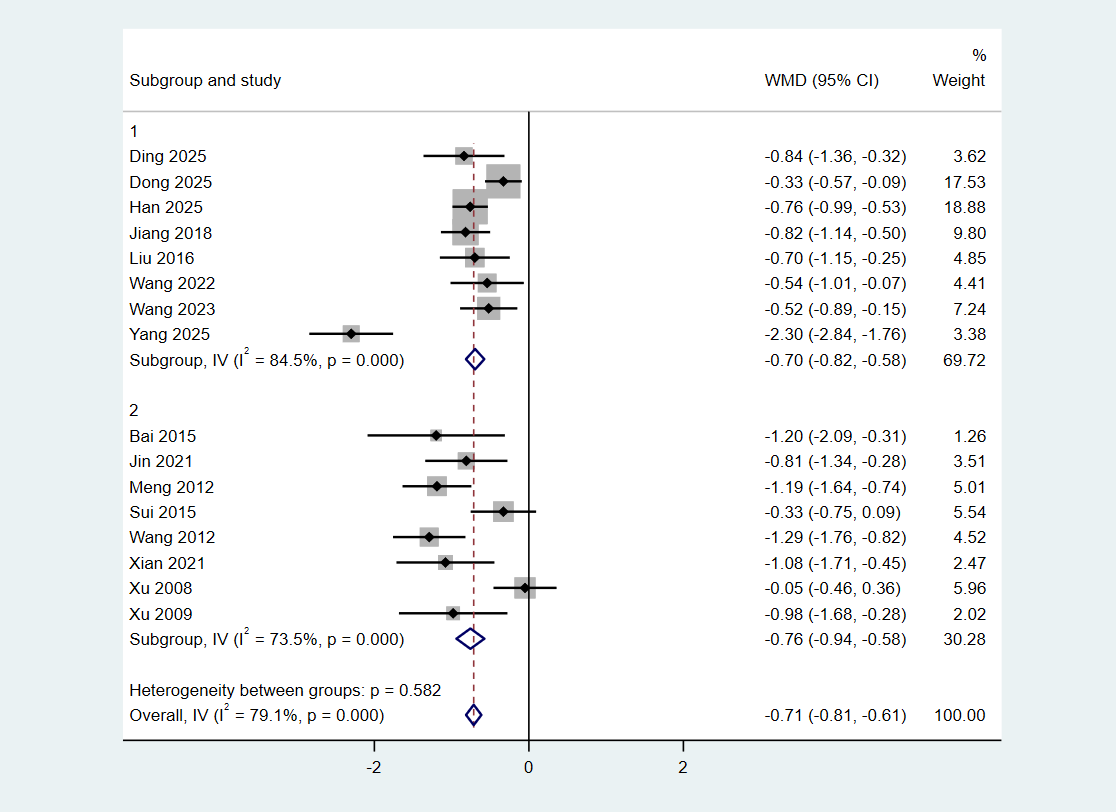


S5.2 Number


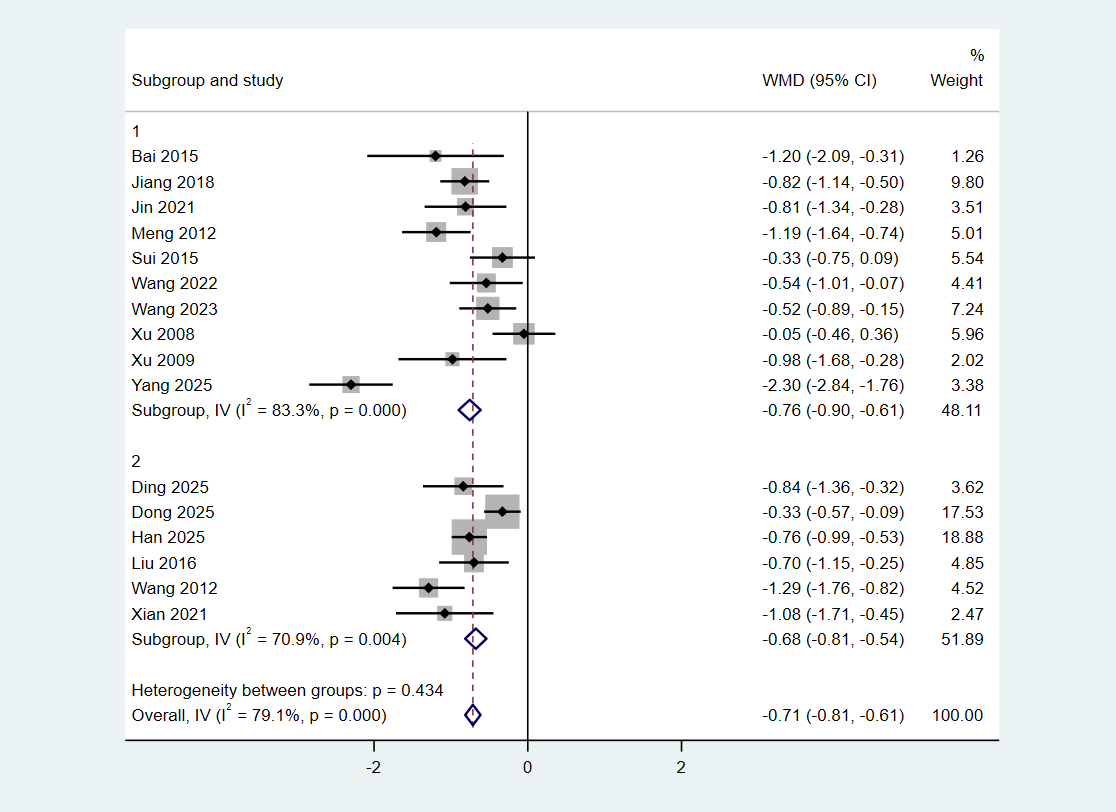


S5.3 Age


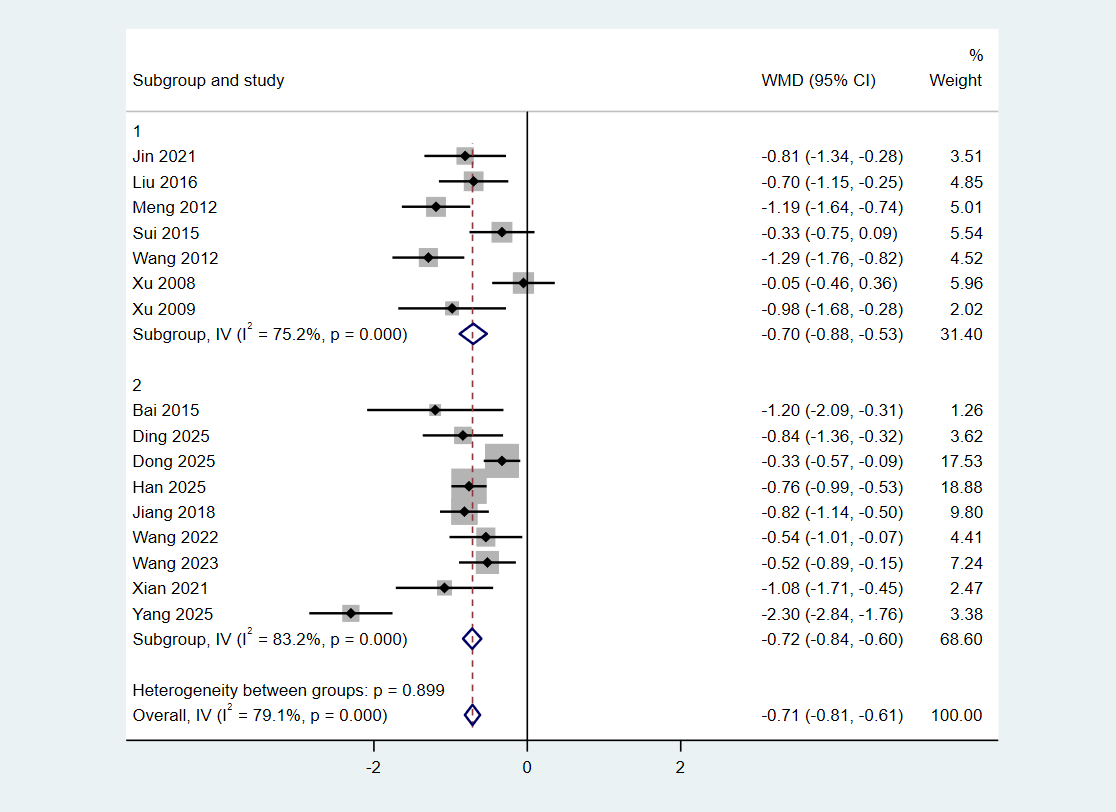


S5.4 Duration


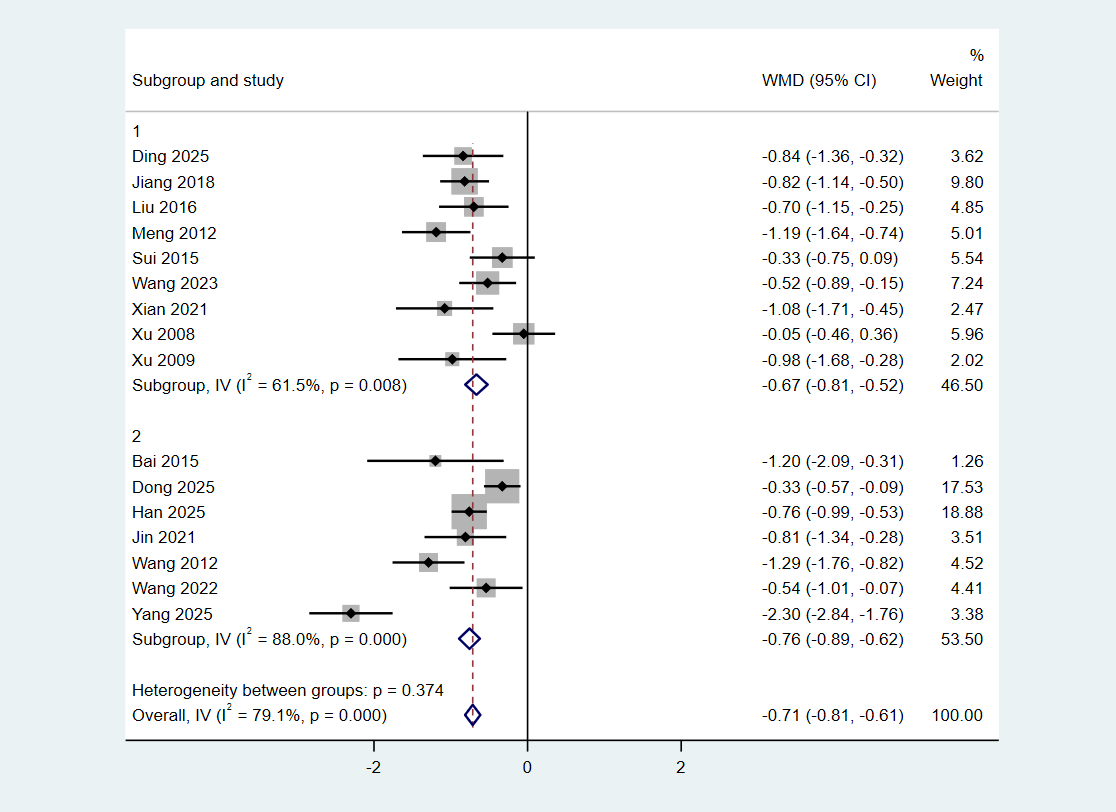


**S6. Subgroup analysis of 2hPG**

S6.1 Random sequence generation


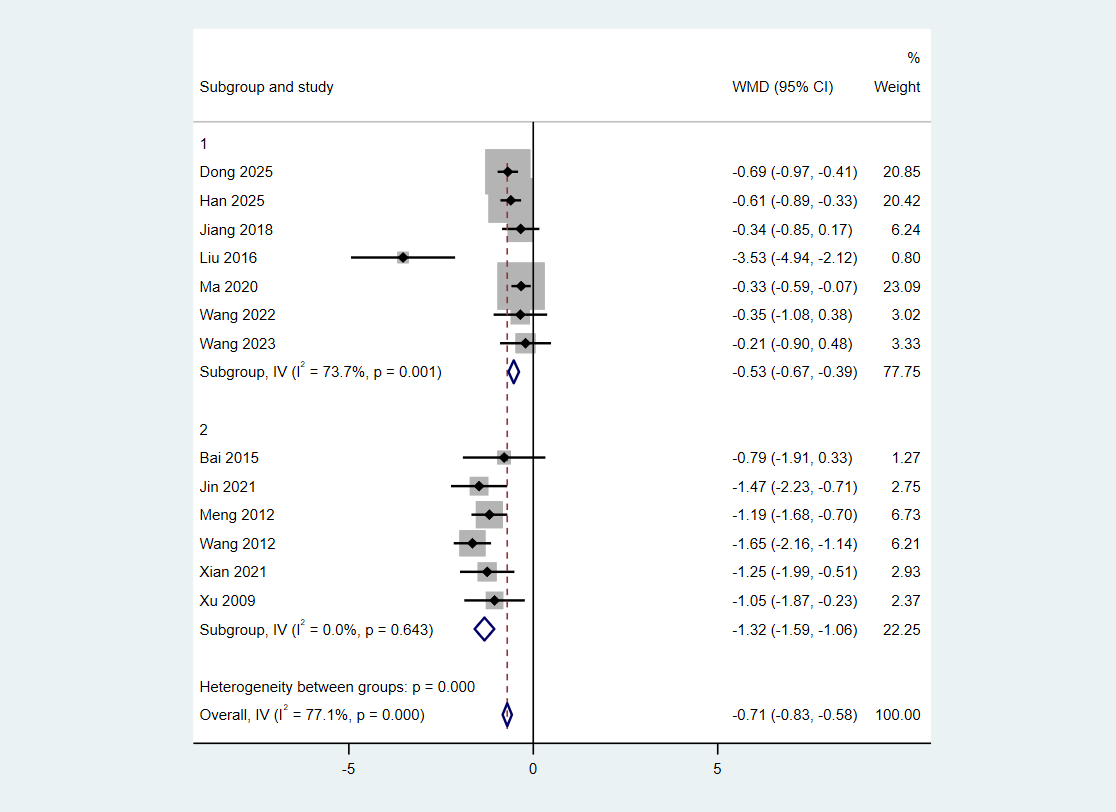


S6.2 Number


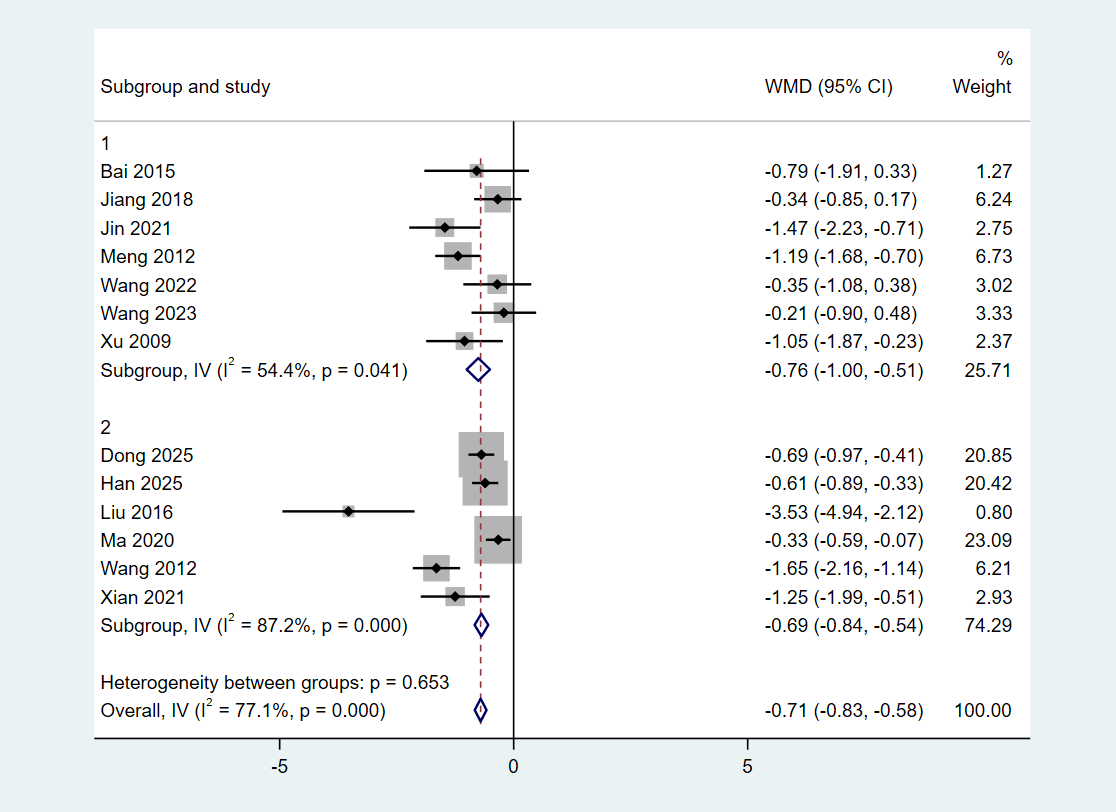


S6.3 Age


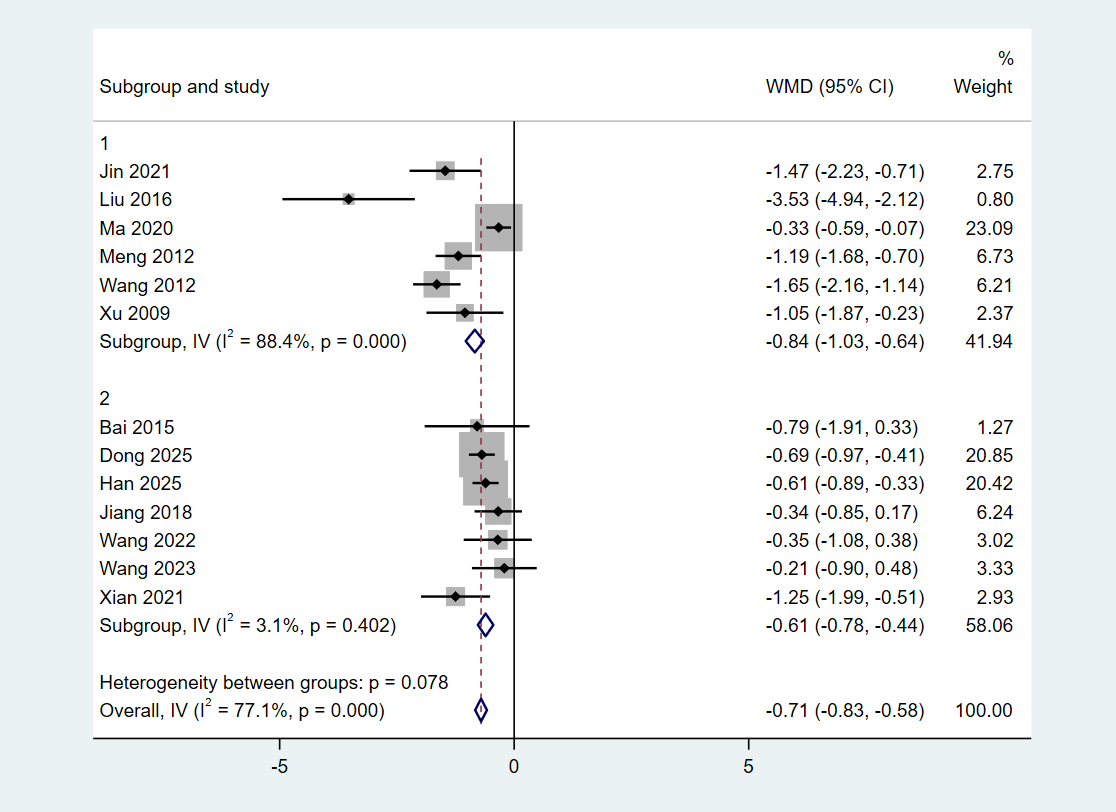


S6.4 Duration


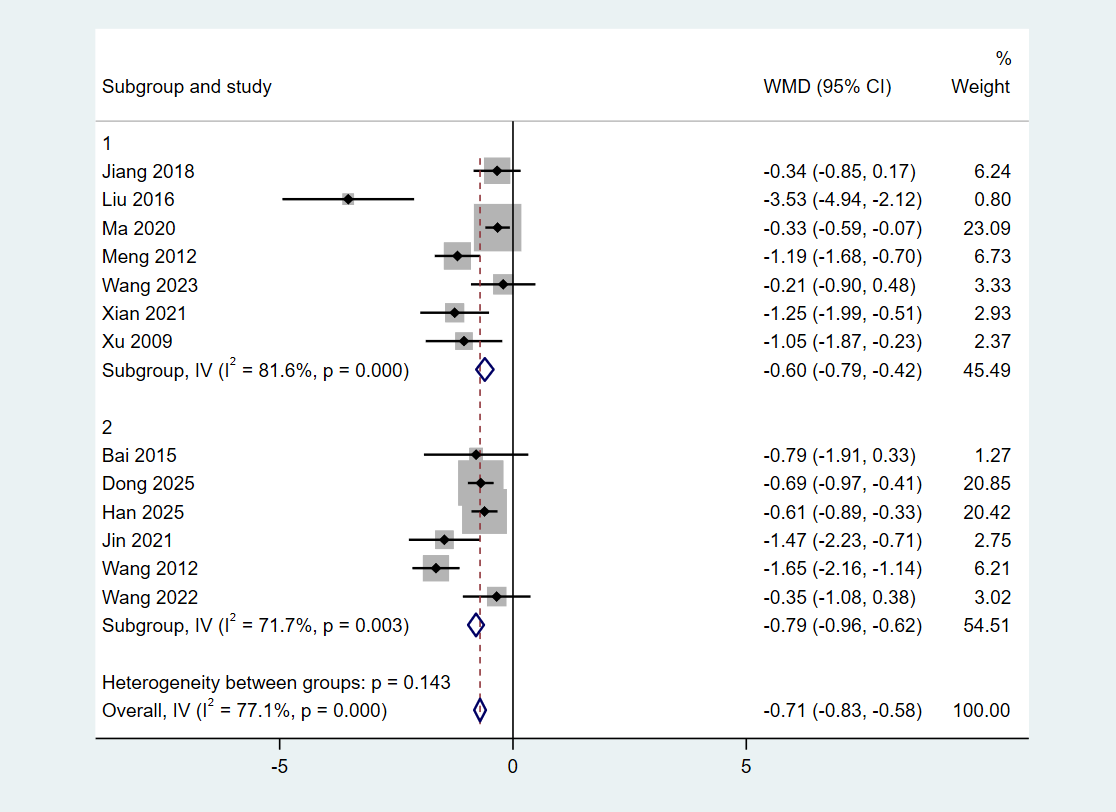


**S7. Subgroup analysis of LDL-C**

S7.1 Random sequence generation


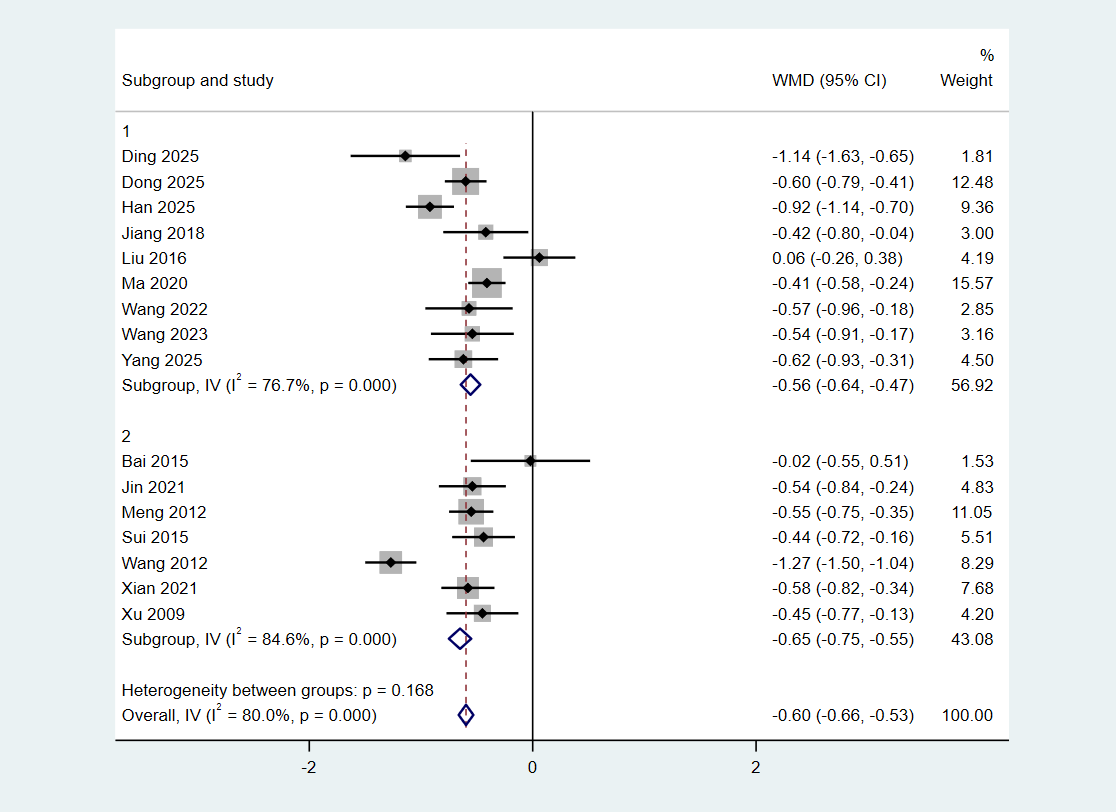


S7.2 Number


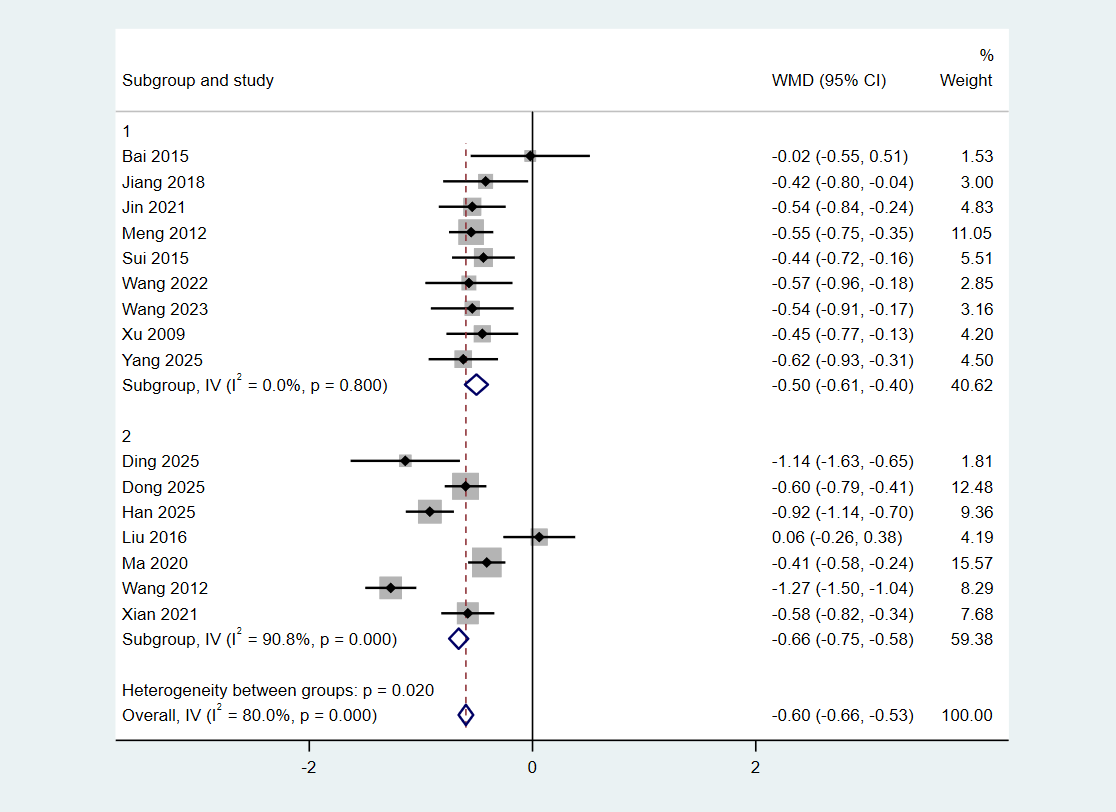


S7.3 Age


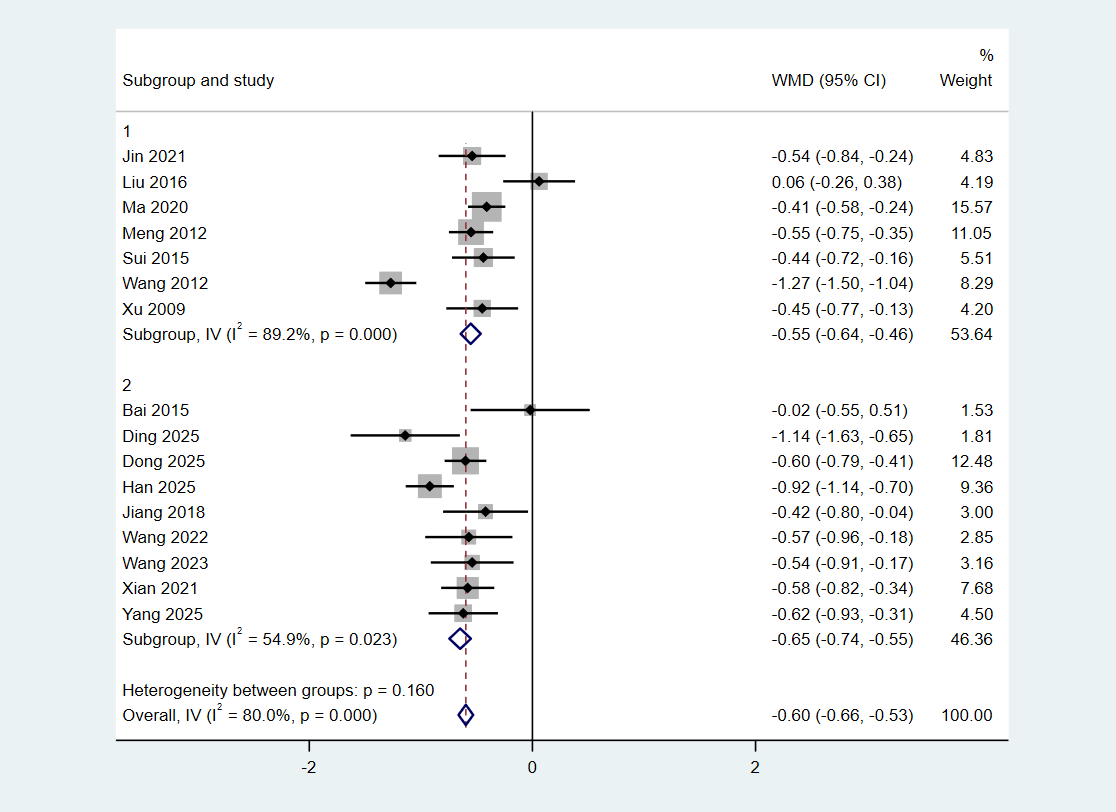


S7.4 Duration


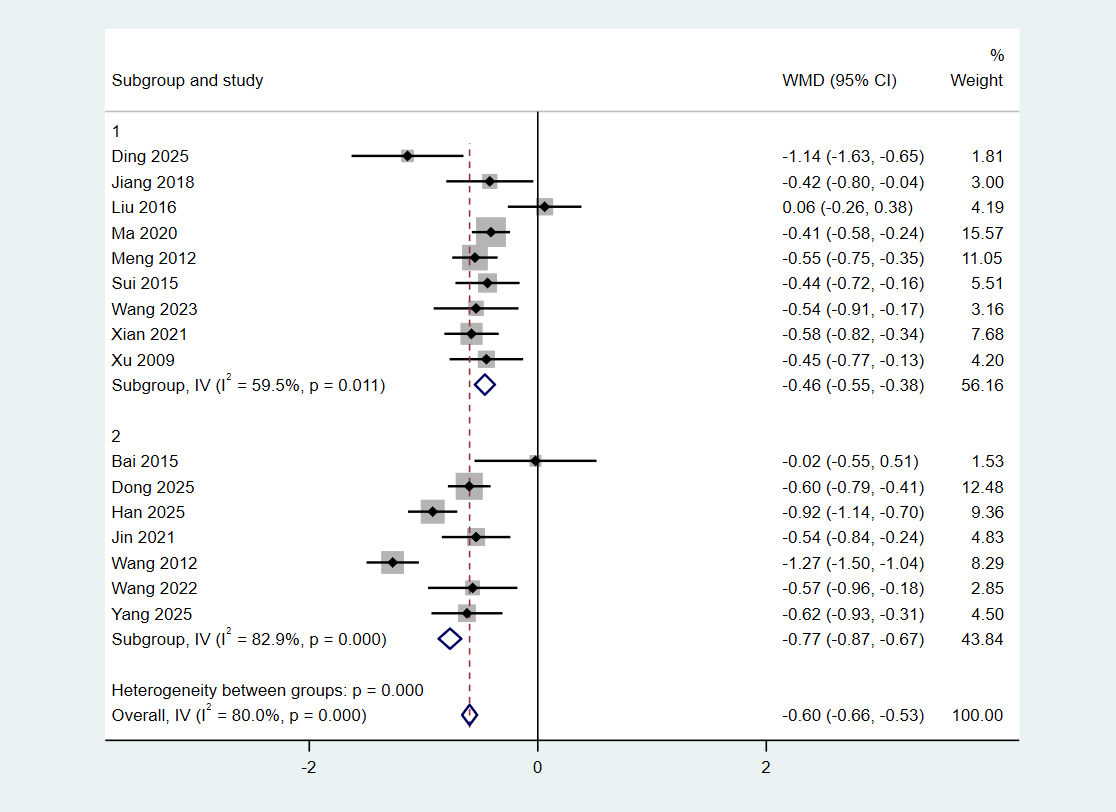


**S8. Subgroup analysis of HDL-C**

S8.1 Random sequence generation


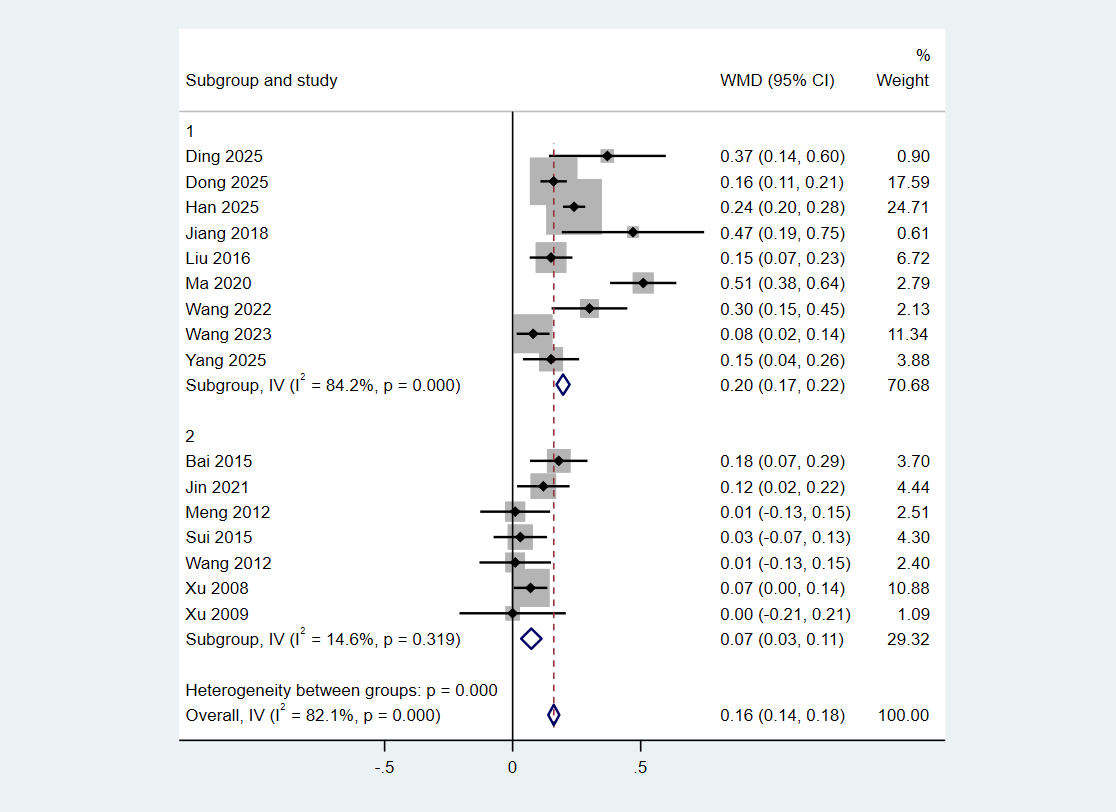


S8.2 Number


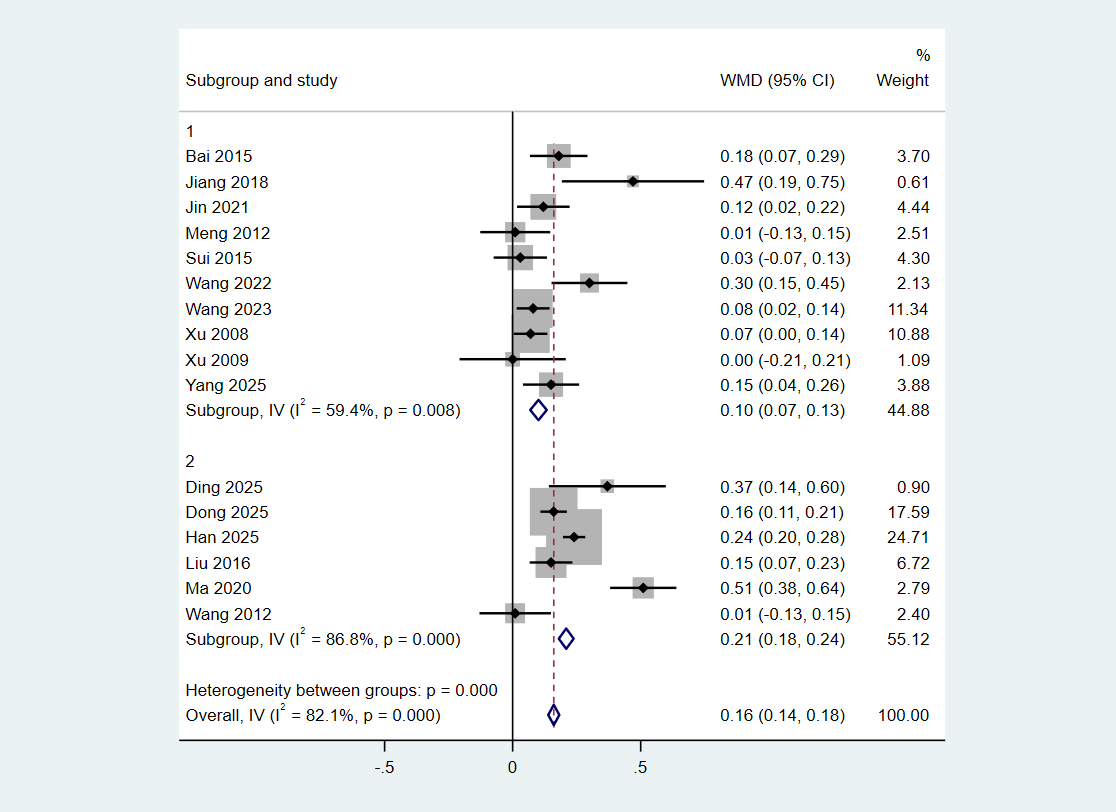


S8.3 Age


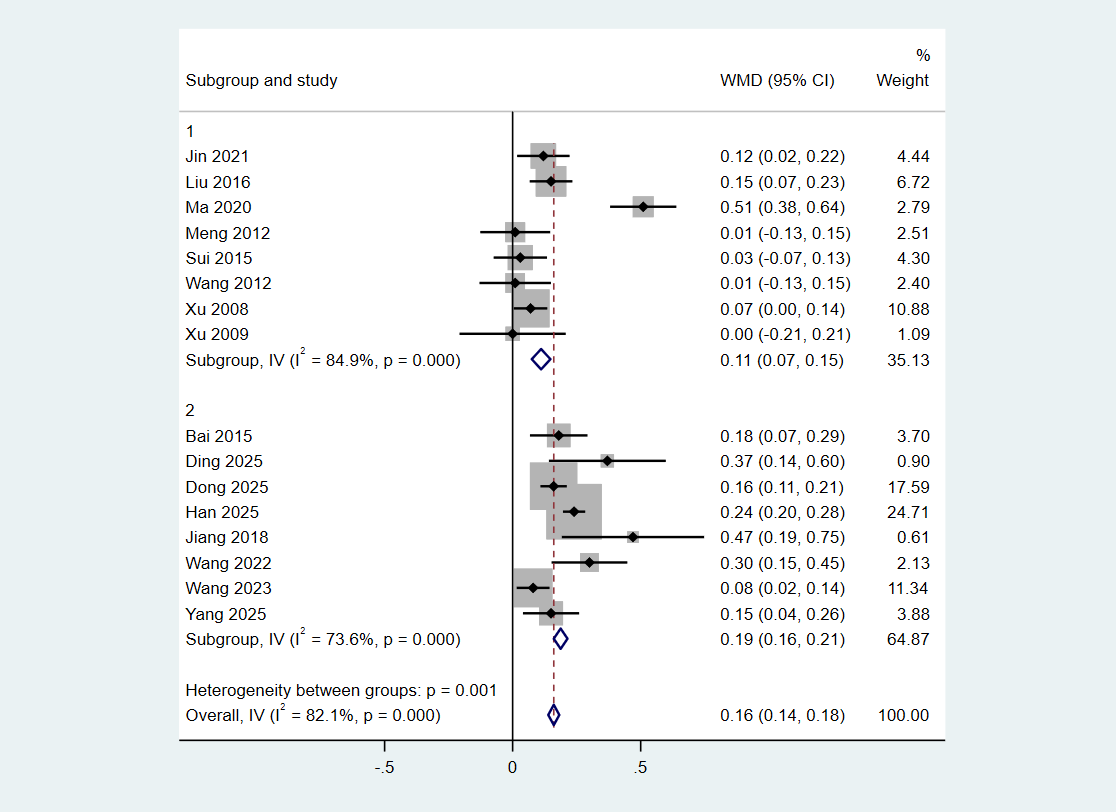


S8.4 Duration


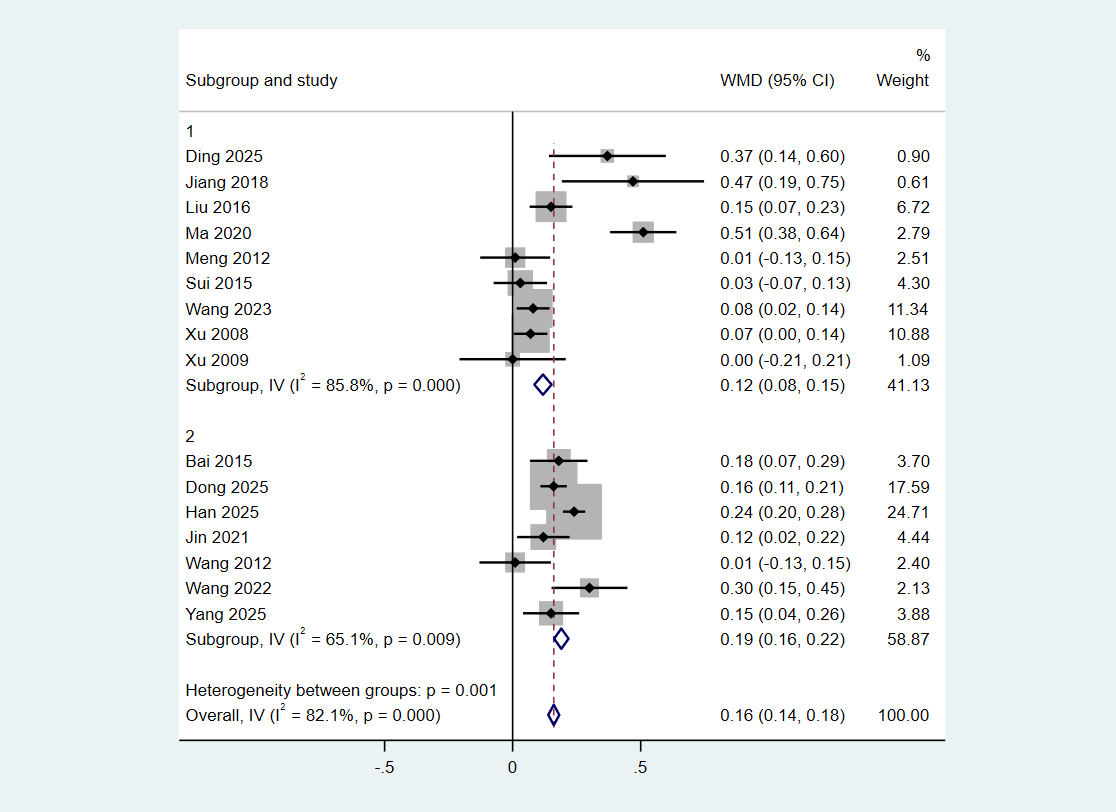


**S9. Meta-regression analysis of SBP**

S9.1 Random sequence generation


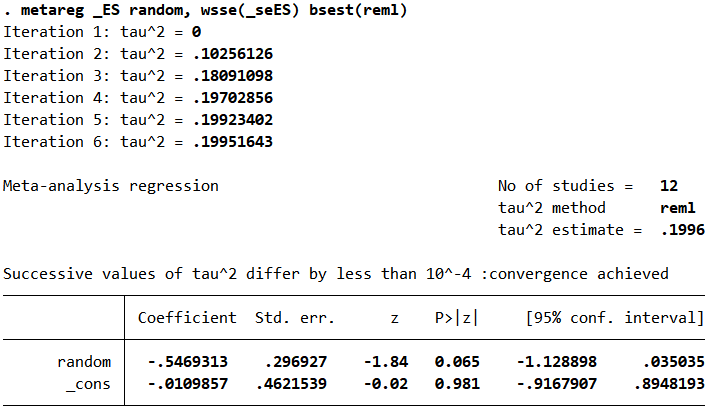


S9.2 Number


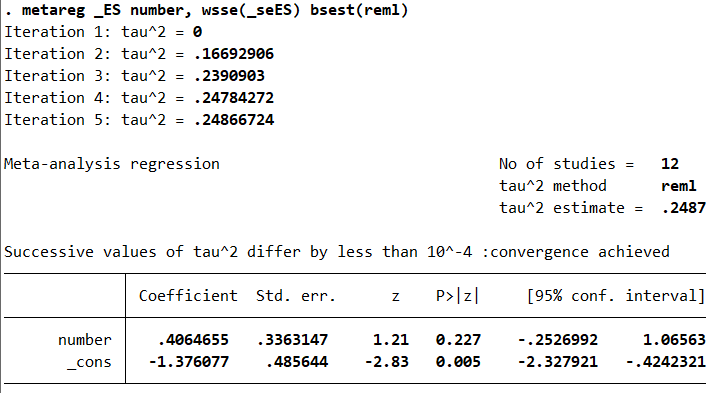


S9.3 Age


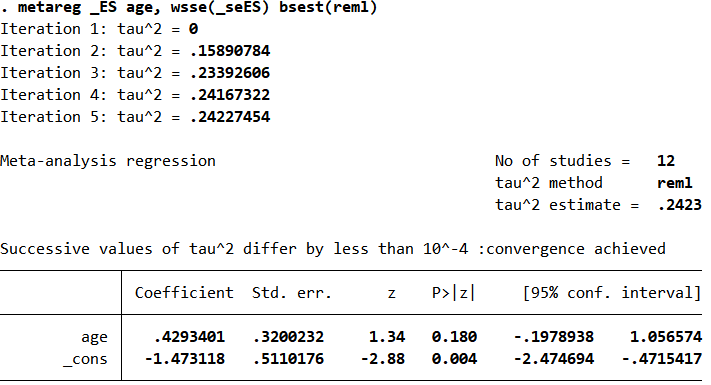


.4 Duration


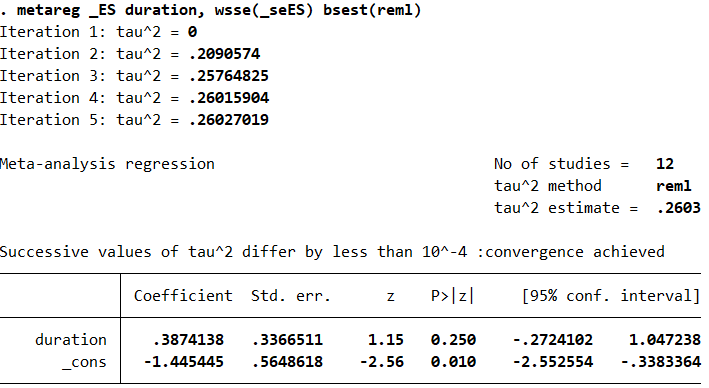


**S20. Meta-regression analysis of DBP**

S20.1 Random sequence generation


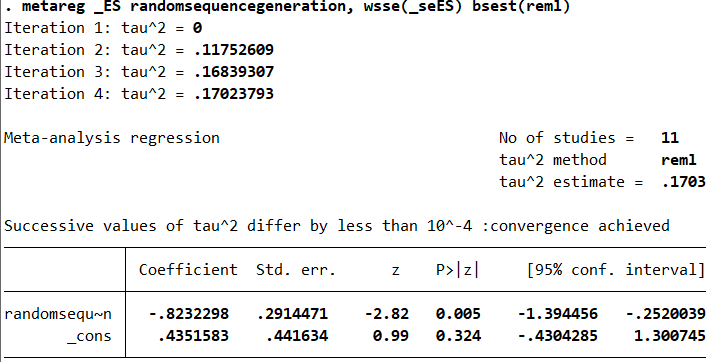


S20.2 Number


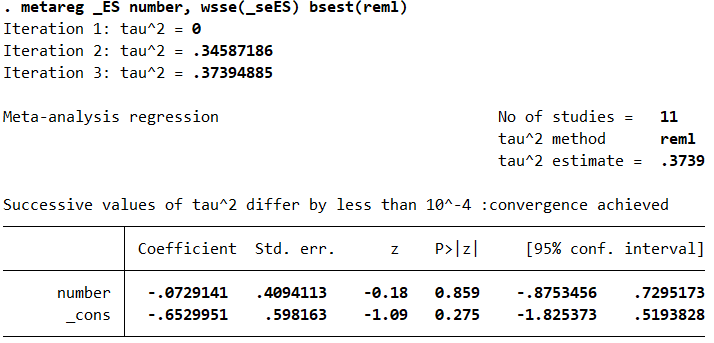


S20.3 Age


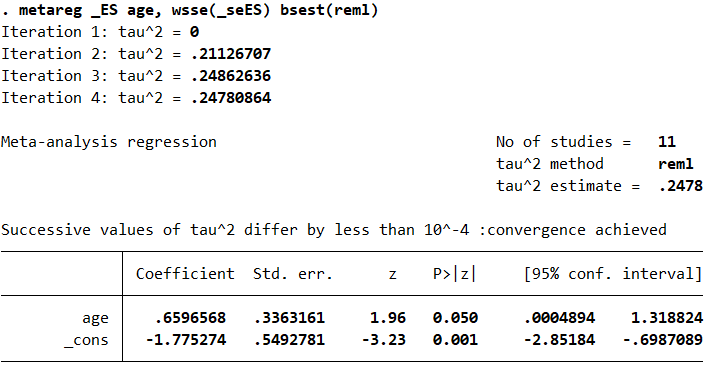


S20.4 Duration


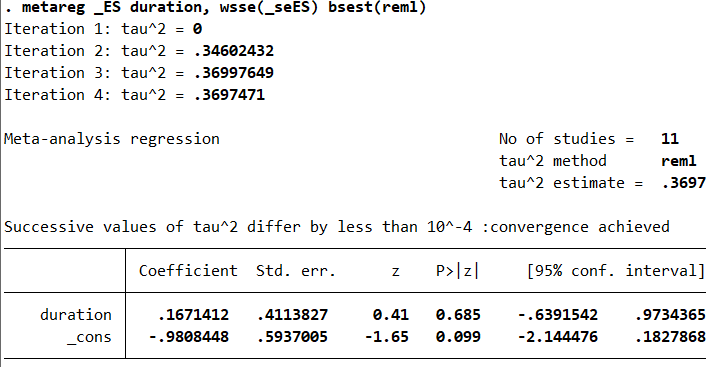


**S21. Meta-regression analysis of FPG**

S21.1 Random sequence generation


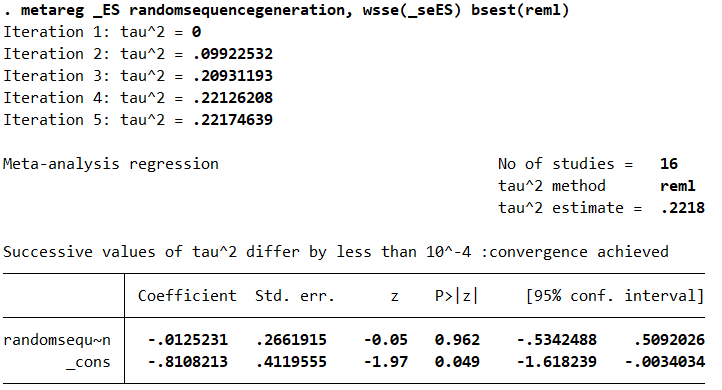


S21.2 Number


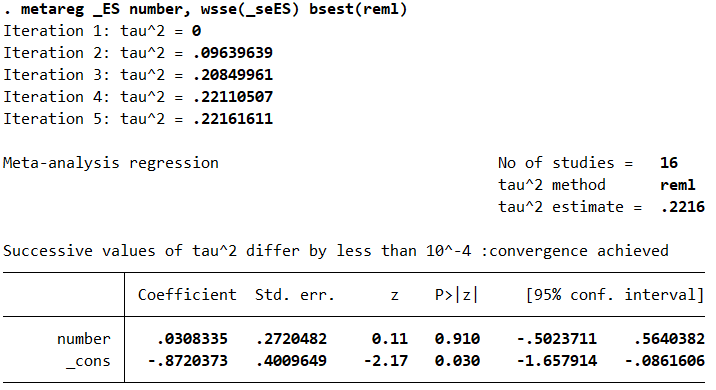


S21.3 Age


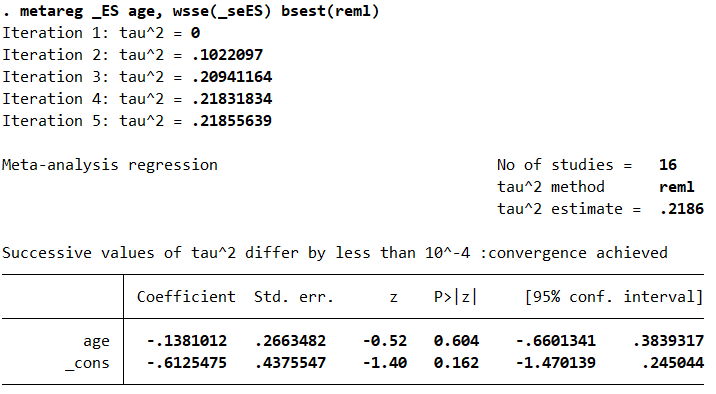


S21.4 Duration


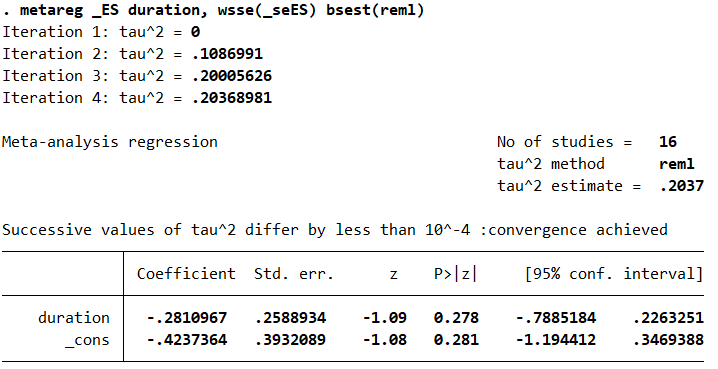


**S22. Meta-regression analysis of 2hPG**

S22.1 Random sequence generation


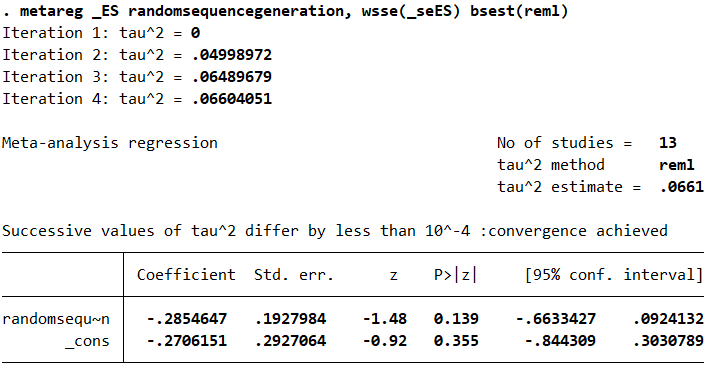


S22.2 Number


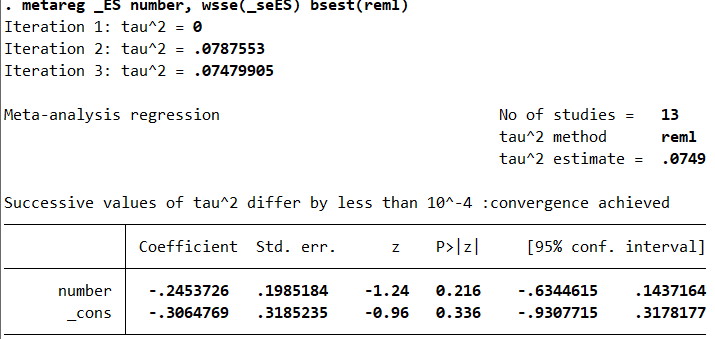


S22.3 Age


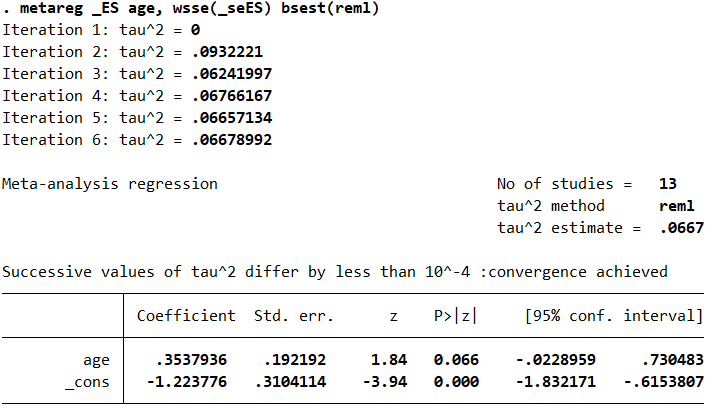


S22.4 Duration


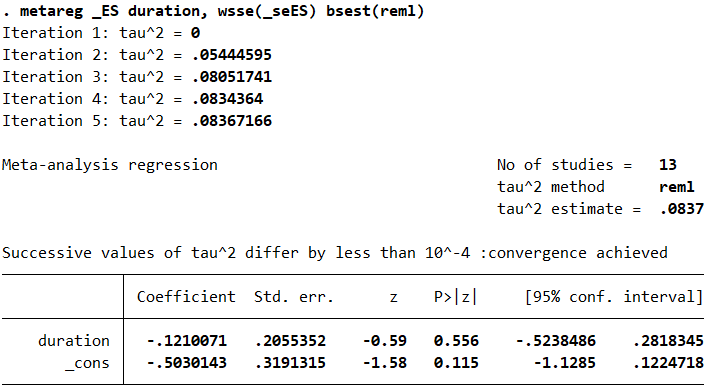


**S23. Meta-regression analysis of LDL-C**

S23.1 Random sequence generation


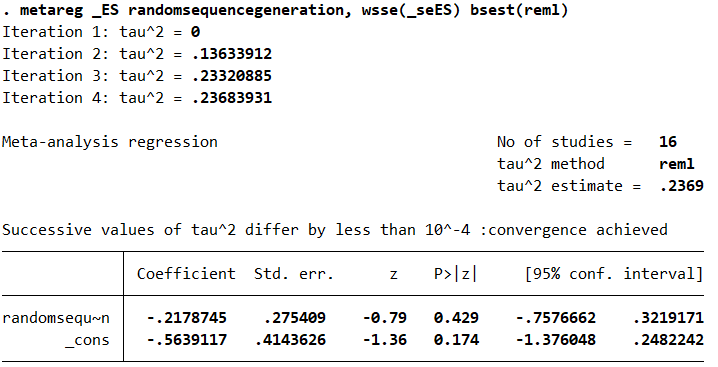


S23.2 Number


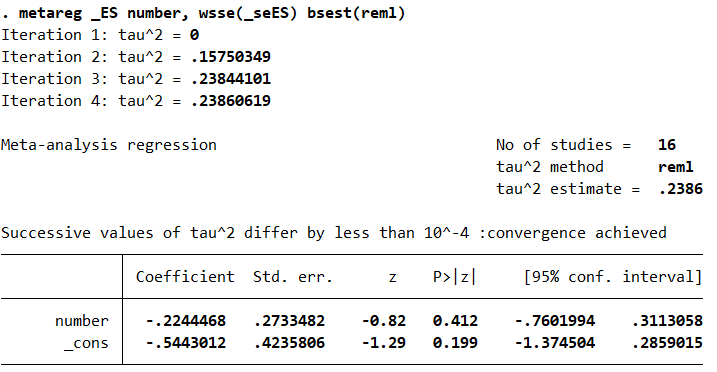


S23.3 Age


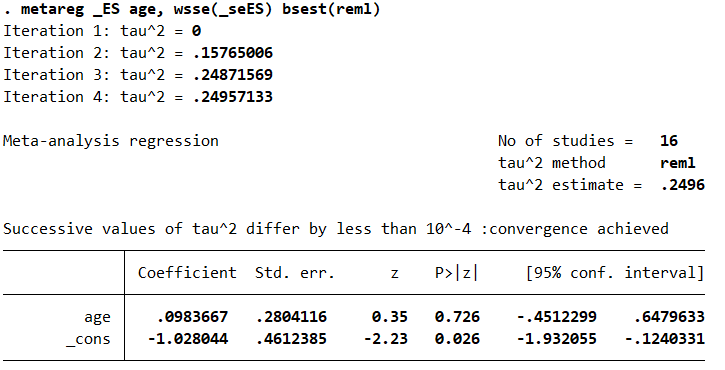


S23.4 Duration


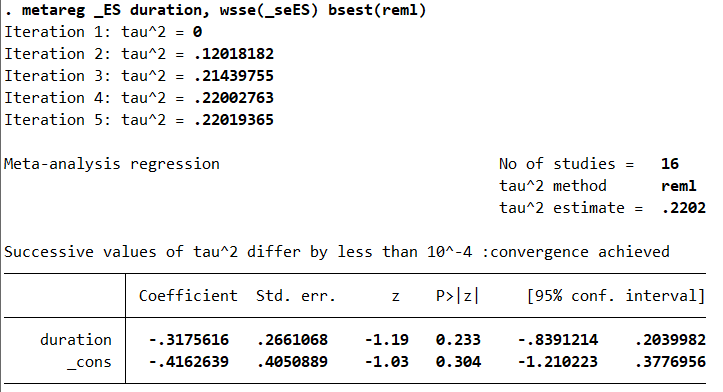


**S24. Meta-regression analysis of HDL-C**

S24.1 Random sequence generation


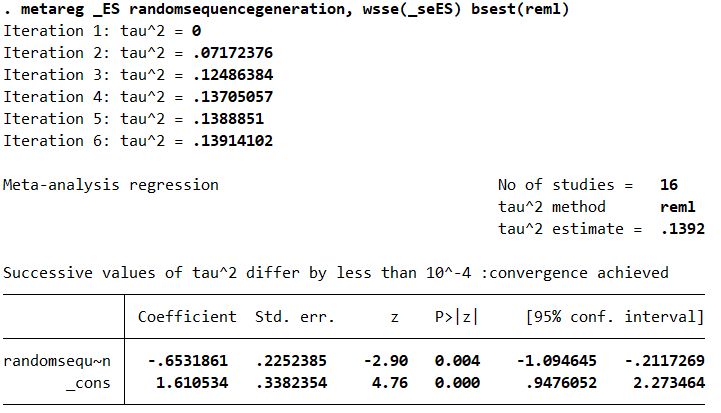


S24.2 Number


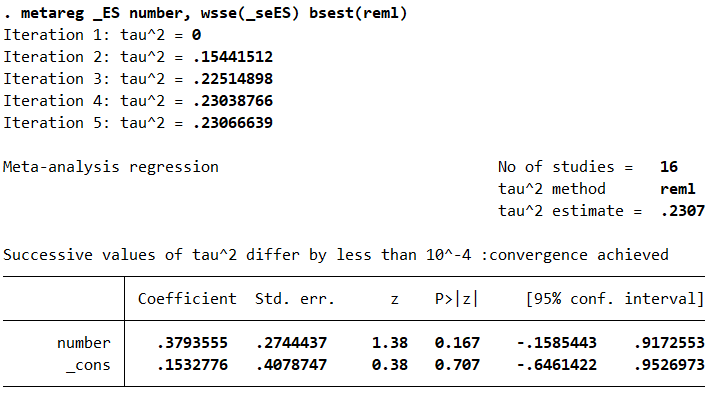


S24.3 Age


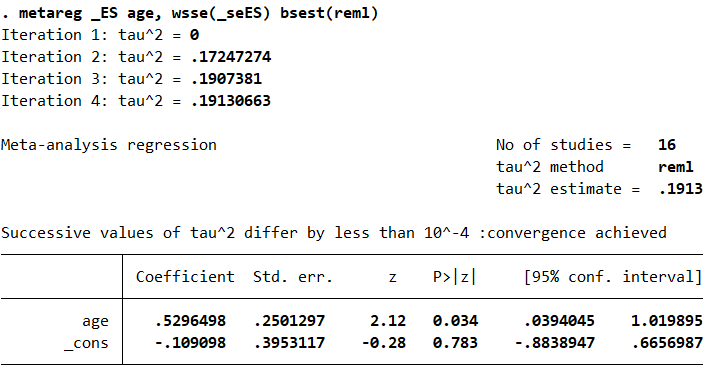


S24.4 Duration


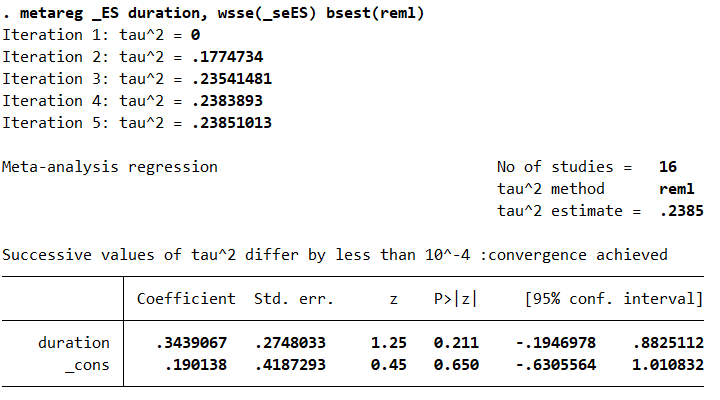


**S25. Publication bias funnel plot of BMI, SBP, DBP, FPG, 2hPG, TG, LDL-C, and HDL-C**

S25.1 BMI


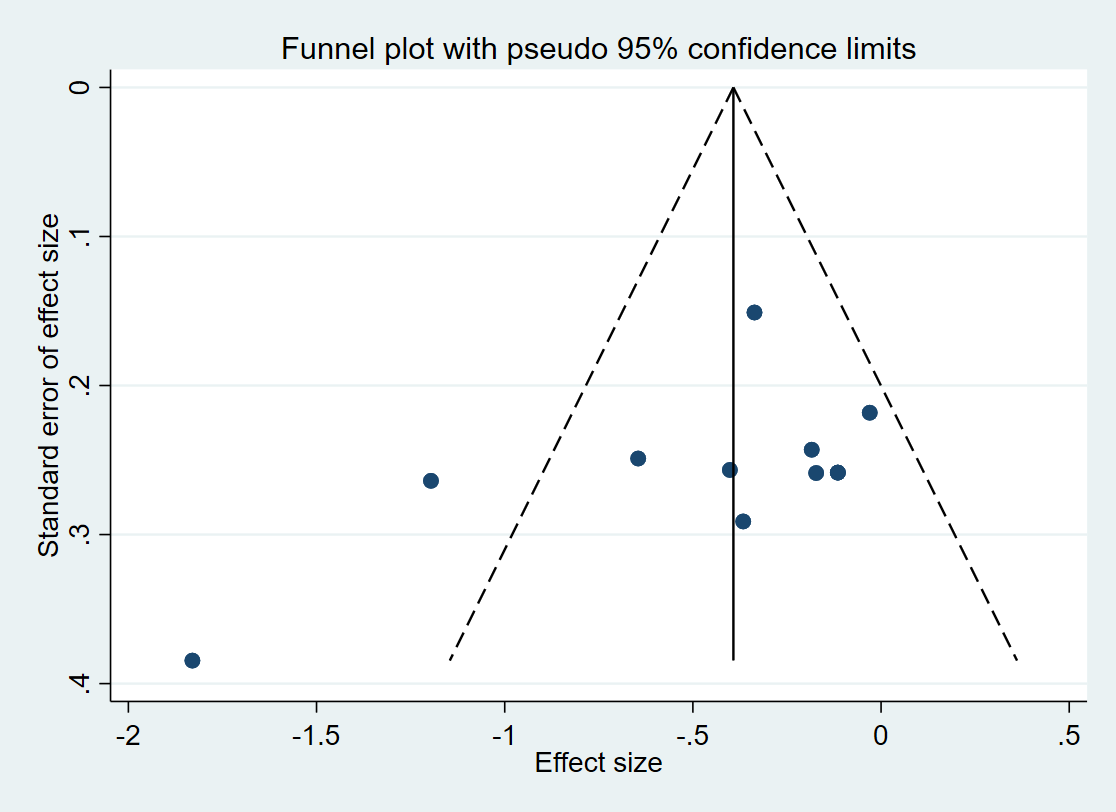


S25.2 SBP


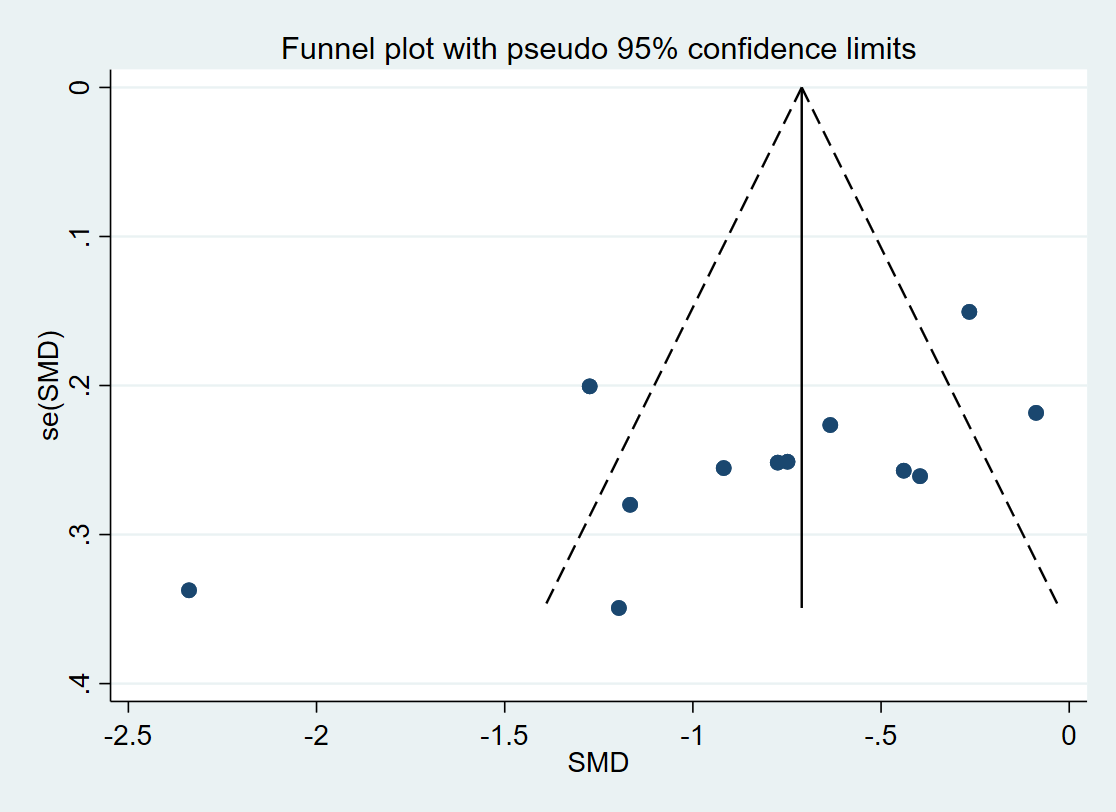


S25.3 DBP


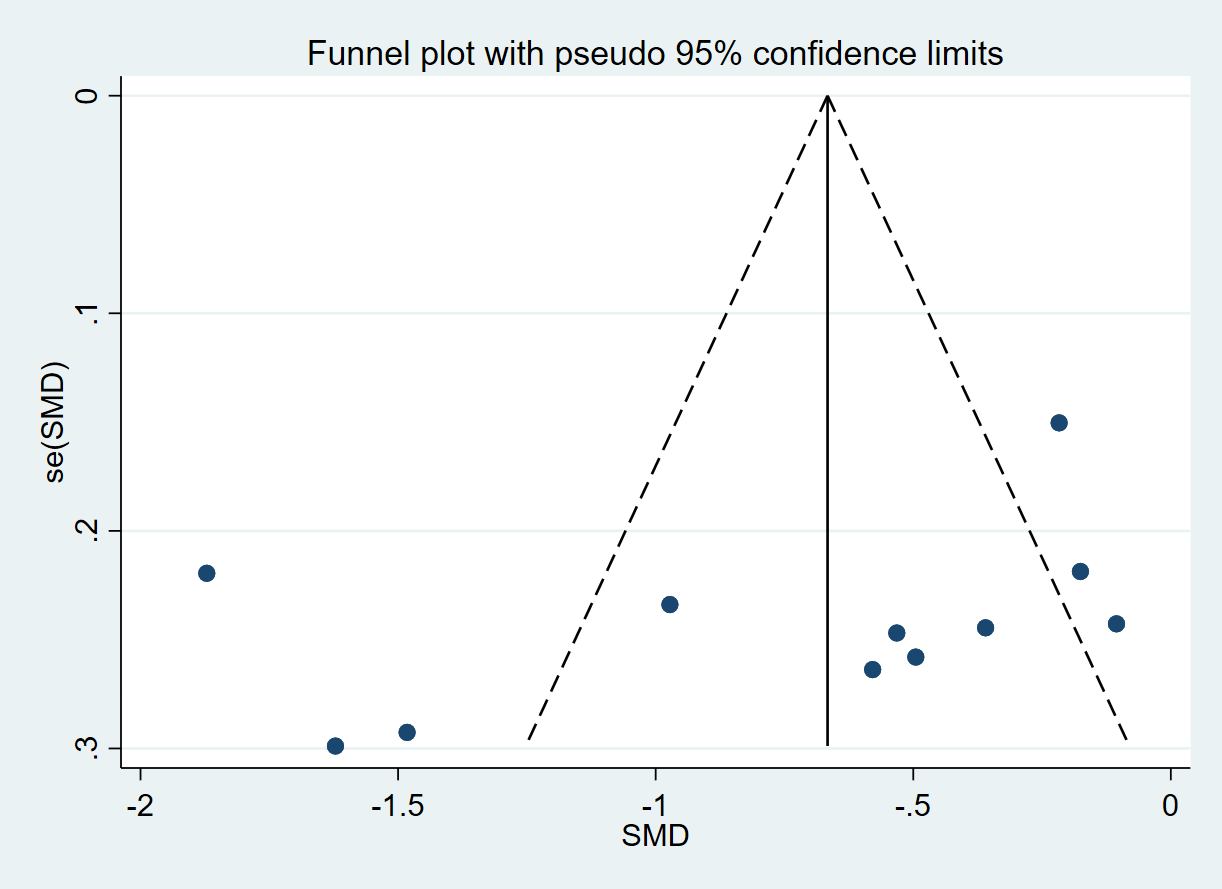


S25.4 FPG


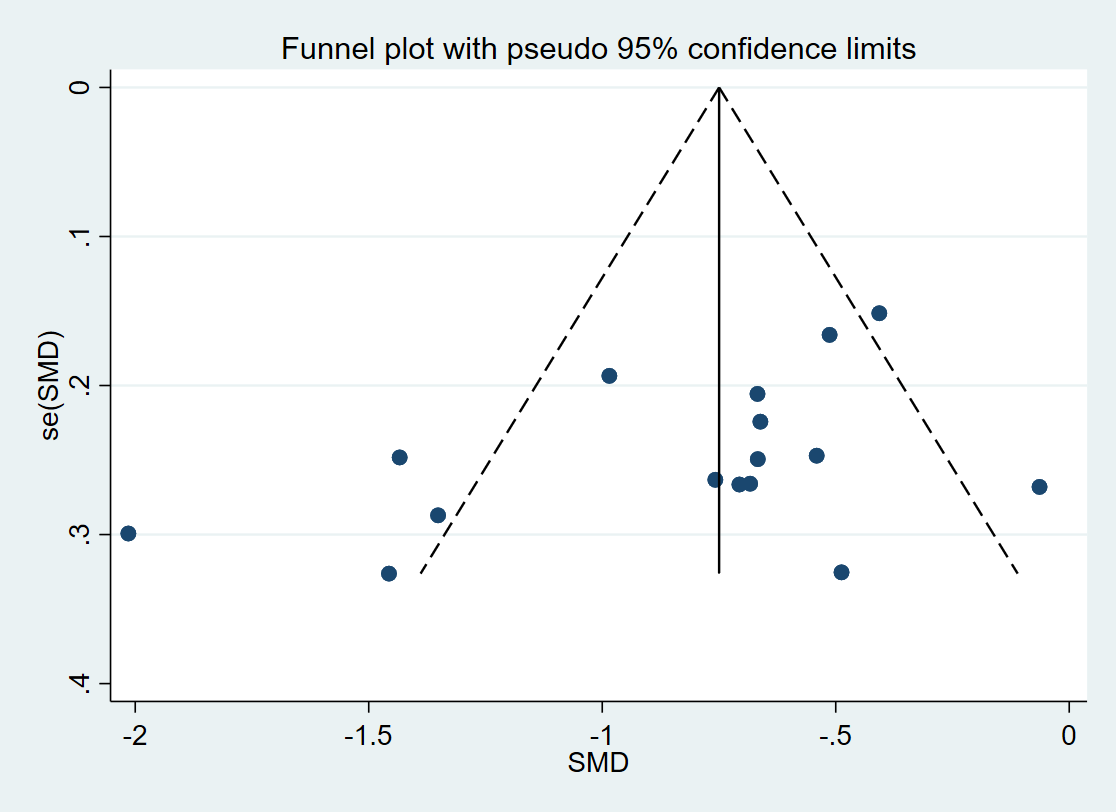


S25.5 2hPG


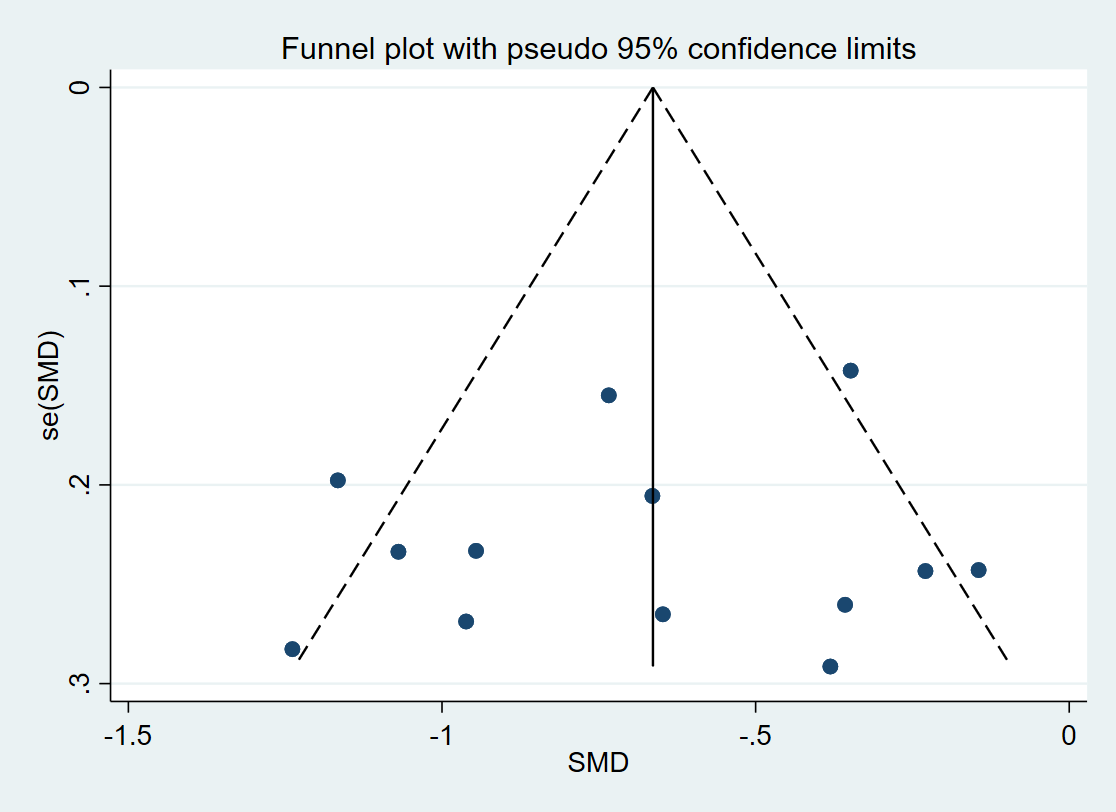


S25.6 TG


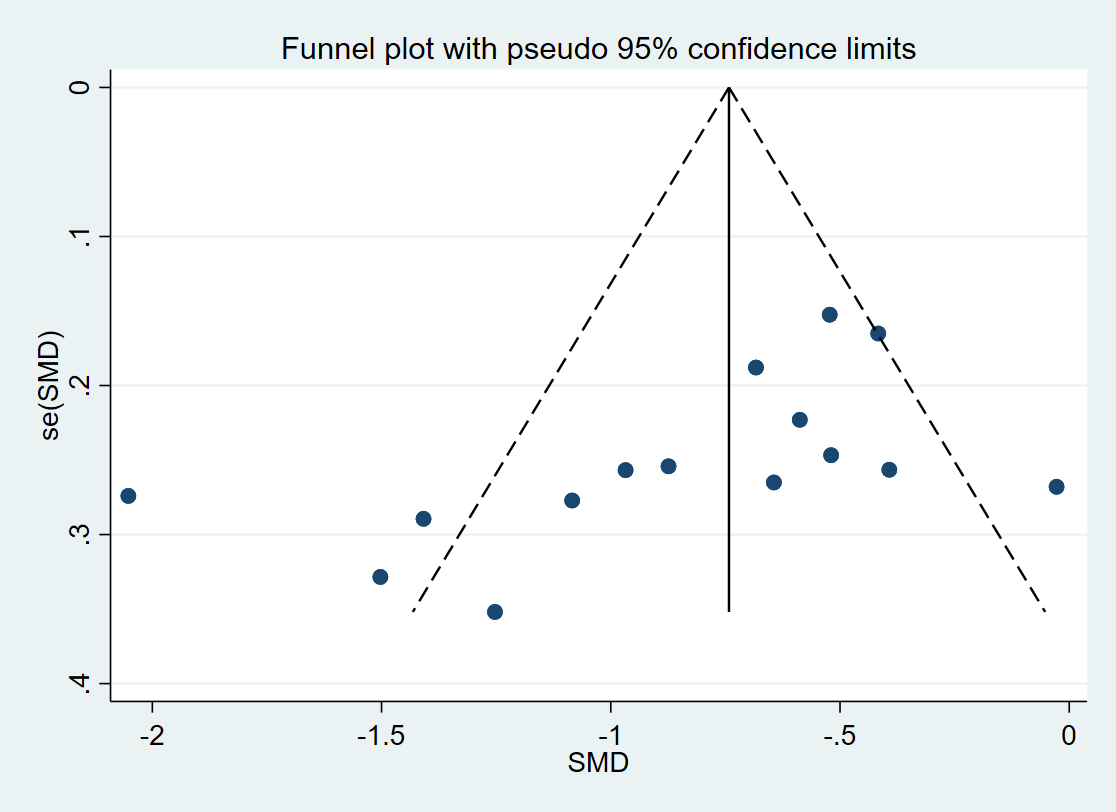


S25.7 LDL-C


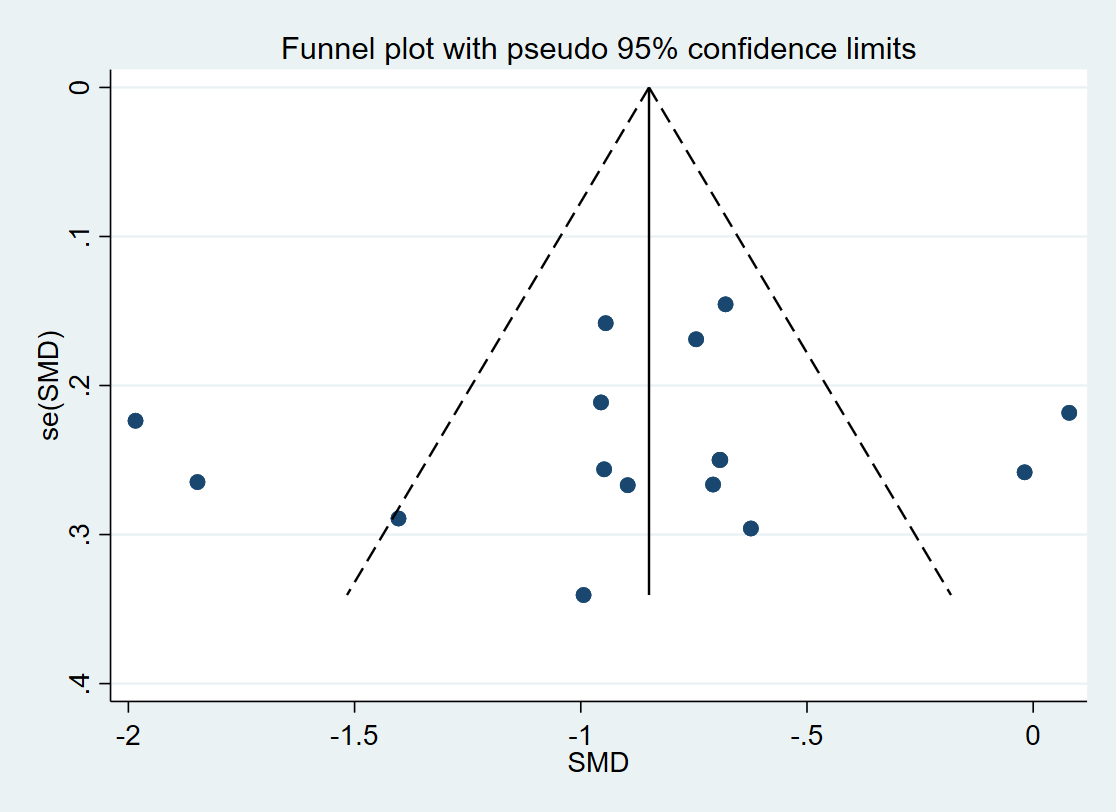


S25.8 HDL-C


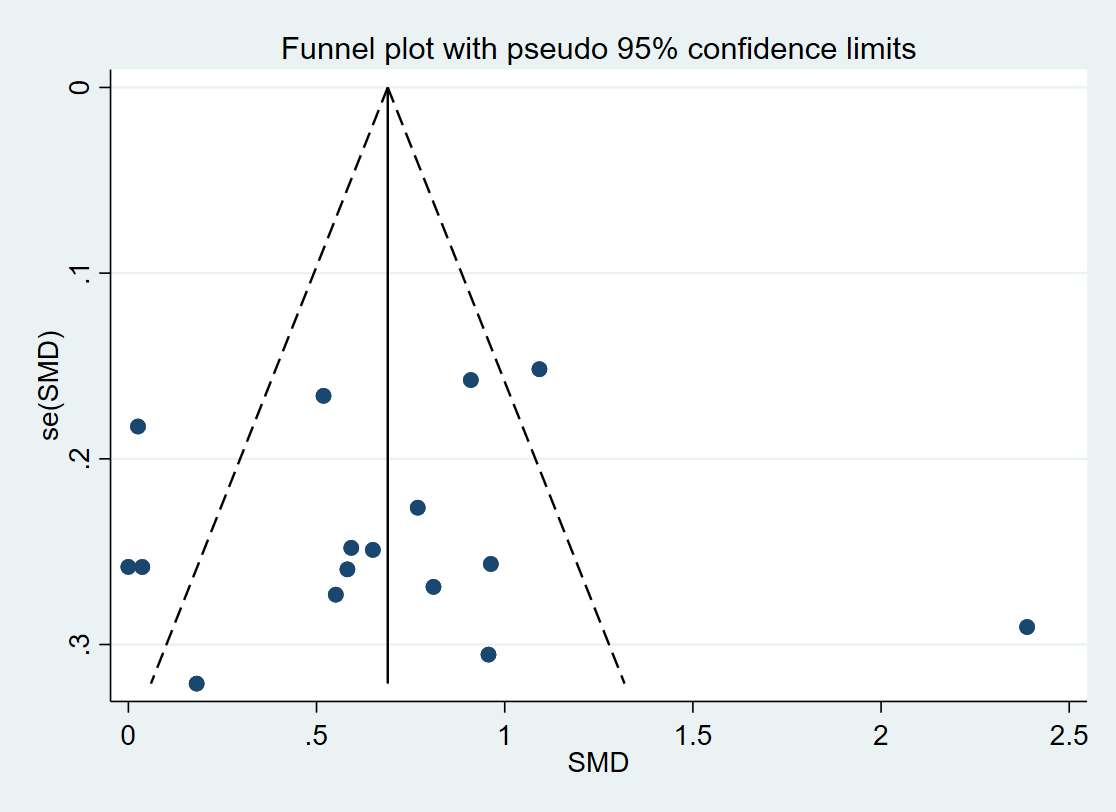


**S26. Egger’s test of BMI, SBP, DBP, FPG, 2hPG, TG, LDL-C, and HDL-C**

S26.1 BMI


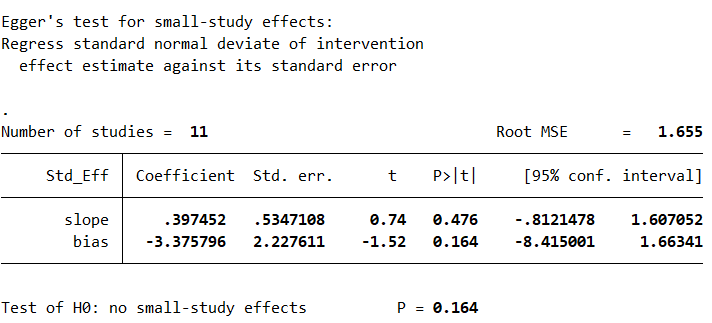


S26.2 SBP


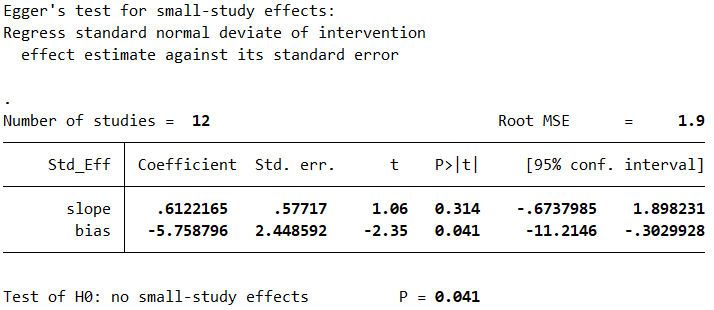


S26.3 DBP


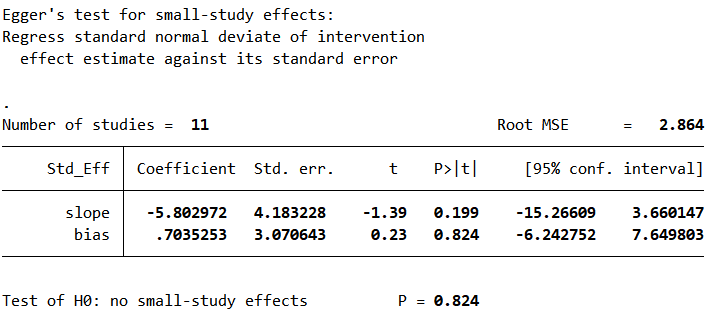


S26.4 FPG


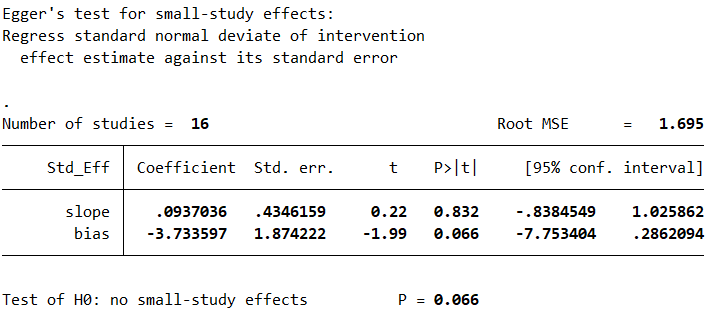


S26.5 2hPG


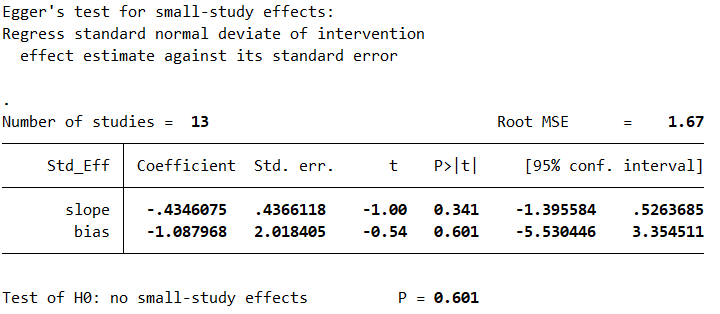


S26.6 TG


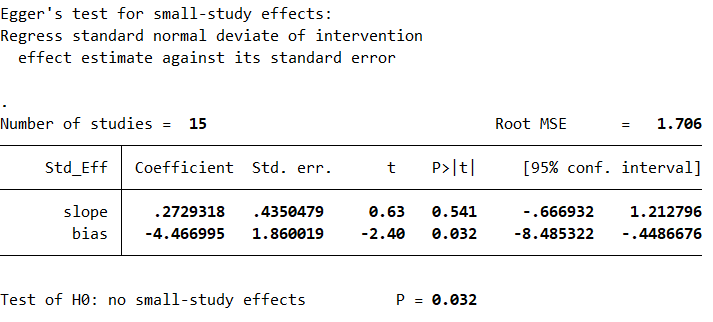


S26.7 LDL-C


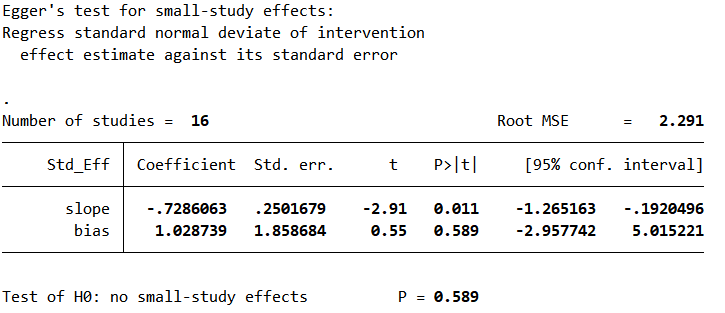


S26.8 HDL-C


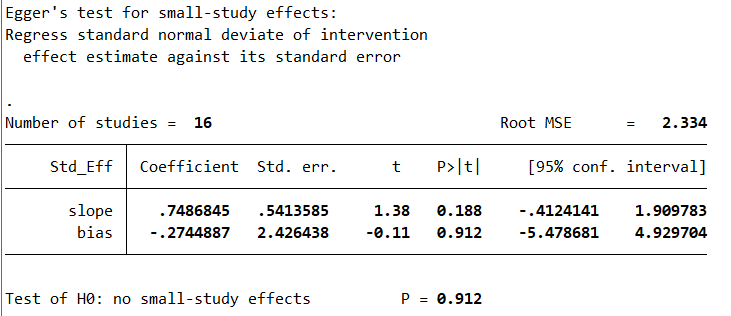


**S27. Trimming and filling method of SBP and TG**

S27.1 SBP


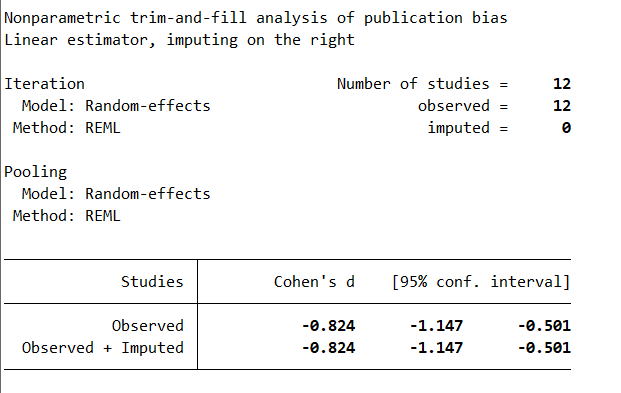


S27.2 TG


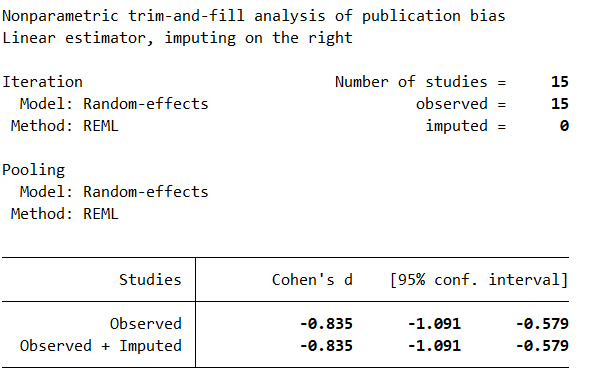

Supplement: Supplementary file 1 [file Supplementaryfile1.docx]
